# Oncogenic signaling pathway-related long non-coding RNAs for predicting prognosis and immunotherapy response in breast cancer

**DOI:** 10.3389/fimmu.2022.891175

**Published:** 2022-08-04

**Authors:** Huamei Li, Hongjia Liu, Qiongyu Hao, Xianglin Liu, Yongzhong Yao, Meng Cao

**Affiliations:** ^1^ Department of General Surgery, Nanjing Drum Tower Hospital, the Affiliated Hospital of Nanjing University Medical School, Nanjing, China; ^2^ State Key Laboratory of Bioelectronics, School of Biological Science & Medical Engineering, Southeast University, Nanjing, China; ^3^ Division of Cancer Research and Training, Charles R. Drew University of Medicine and Science, Los Angeles, CA, United States

**Keywords:** breast cancer, oncogenic signaling pathway, long non-coding RNA, immune infiltration, risk score, prognosis, immunotherapy

## Abstract

**Background:**

The clinical outcomes of breast cancer (BC) are unpredictable due to the high level of heterogeneity and complex immune status of the tumor microenvironment (TME). When set up, multiple long non-coding RNA (lncRNA) signatures tended to be employed to appraise the prognosis of BC. Nevertheless, predicting immunotherapy responses in BC is still essential. LncRNAs play pivotal roles in cancer development through diverse oncogenic signal pathways. Hence, we attempted to construct an oncogenic signal pathway–based lncRNA signature for forecasting prognosis and immunotherapy response by providing reliable signatures.

**Methods:**

We preliminarily retrieved RNA sequencing (RNA-seq) data from The Cancer Genome Atlas (TCGA) database and extracted lncRNA profiles by matching them with GENCODE. Following this, Gene Set Variation Analysis (GSVA) was used to identify the lncRNAs closely associated with 10 oncogenic signaling pathways from the TCGA-BRCA (breast-invasive carcinoma) cohort and was further screened by the least absolute shrinkage and selection operator Cox regression model. Next, an lncRNA signature (OncoSig) was established through the expression level of the final 29 selected lncRNAs. To examine survival differences in the stratification described by the OncoSig, the Kaplan–Meier (KM) survival curve with the log-rank test was operated on four independent cohorts (n = 936). Subsequently, multiple Cox regression was used to investigate the independence of the OncoSig as a prognostic factor. With the concordance index (C-index), the time-dependent receiver operating characteristic was employed to assess the performance of the OncoSig compared to other publicly available lncRNA signatures for BC. In addition, biological differences between the high- and low-risk groups, as portrayed by the OncoSig, were analyzed on the basis of statistical tests. Immune cell infiltration was investigated using gene set enrichment analysis (GSEA) and deconvolution tools (including CIBERSORT and ESTIMATE). The combined effect of the Oncosig and immune checkpoint genes on prognosis and immunotherapy was elucidated through the KM survival curve. Ultimately, a pan-cancer analysis was conducted to attest to the prevalence of the OncoSig.

**Results:**

The OncoSig score stratified BC patients into high- and low-risk groups, where the latter manifested a significantly higher survival rate and immune cell infiltration when compared to the former. A multivariate analysis suggested that OncoSig is an independent prognosis predictor for BC patients. In addition, compared to the other four publicly available lncRNA signatures, OncoSig exhibited superior predictive performance (AUC = 0.787, mean C-index = 0.714). The analyses of the OncoSig and immune checkpoint genes clarified that a lower OncoSig score meant significantly longer survival and improved response to immunotherapy. In addition to BC, a high OncoSig score in several other cancers was negatively correlated with survival and immune cell infiltration.

**Conclusions:**

Our study established a trustworthy and discriminable prognostic signature for BC patients with similar clinical profiles, thus providing a new perspective in the evaluation of immunotherapy responses. More importantly, this finding can be generalized to be applicable to the vast majority of human cancers.

## Introduction

Breast cancer (BC) is one of the most common cancers worldwide and is the major cause of cancer-related death in women (1). Based on the differences in historical and molecular levels, BC can be classified into five subtypes: HER2-positive (HER2), triple-negative/basal (Basal), normal-like (Normal), and luminal-A and B (LumA and LumB) (2, 3). So far, a combination of surgery, chemotherapy, hormone therapy, radiation therapy, and targeted therapy has been administered in the treatment of BC (4). Unfortunately, the considerable functional heterogeneity of diverse immune cell types in BC intrinsic subtypes has contributed to variations in the prognosis of BC patients (5, 6). With progress in research, the immune system has been found to play a vital role in tumorigenesis and cancer development (6). Previous reviews have summarized that protumorigenic and pro-inflammatory immune cells in the tumor microenvironment (TME) in BC consist of myeloid-derived suppressor cells (MDSCs), M2 macrophages, neutrophils, Th2 CD4+, Th17 CD4+, and FoxP3+ CD4+ T cells (T-regs) as well as T helper cells of type 1 (Th1) CD4+, CD8+ cytotoxic T lymphocytes (CTLs), M1 macrophages, dendritic cells (DCs), and natural killer (NK) cells, respectively (7, 8). Tumor-infiltrating lymphocytes (TILs) have been considered as predictive (9, 10) and prognostic (11, 12) biomarkers of immunotherapy in patients undergoing TNBC and HER2 BC treatment. However, to our current knowledge, there will be challenges to putting TIL assessment into clinical practice, mainly due to the lack of best TILs, upskilled clinicians, and prospective clinical trials (12). Thus, to obtain precise evidence for setting up an appropriate individual treatment strategy, there still remains an urgent need for credible signatures that provide trustworthy evidence for evaluating prognosis and response to immunotherapy in BC.

Long non-coding RNA (lncRNA) is an abundant type of RNA in the human transcriptome, with a transcript length of over 200 nucleotides, which lacks the capability to code protein (13). LncRNAs participate in 70% of gene expressions by enhancing or inhibiting the effects of DNA, RNA, and protein (14) and are strongly associated with cancer development, progression, and prognosis (15). Furthermore, lncRNAs play essential roles in many oncogenic signal pathways (16). Studies have confirmed that Linc00514 has the ability to regulate tumorigenicity and promote metastasis through the Jagged1-mediated Notch signal pathway in BC (17). While lncRNA *AU021063* promotes BC metastasis by activating the Mek/Erk signaling pathway (18), the lncRNA *NIKLA* could inhibit NF-κB activation. Effectively, low levels of *NIKLA* in BC may be the underlying mechanism behind BC metastasis and poor prognosis, whilst a rising *NIKLA* level can be stimulated by Nuclear factor kappa-B (NF-κB), thus generating negative feedback (16, 19).

Furthermore, the results from previous research that built an oncogenic lncRNA landscape for BC identified 55 lncRNAs that are primarily involved in the regulation of immune system activation, TGF*β*, and Jak-STAT signal pathways (20). Previous studies have affirmed that the prediction of cancer prognosis can be achieved by establishing lncRNA signatures. Hong et al. constructed a predictive landscape for human hepatocellular carcinoma by examining 36 pairs of immune-related lncRNAs (21). An 11-lncRNA prognosis signature that correlates with immune cell infiltration in BC has also been set up (22). Furthermore, a novel upregulated-lncRNA GATA3-AS1 contributing to tumor development and immune evasion by degrading *GATA3* and stabilizing *PD-L1* has been found in TNBC (23), suggesting that lncRNA has a strong association with cancer progression as it affects the immune checkpoint. Currently, the signatures of oncogenic signaling pathways and tumor immune infiltration–associated lncRNAs have been preliminarily explored but without sufficient description. The purpose of our study is to build a novel signature that displays tumor immune infiltration–related lncRNA identification through the analysis of oncogenic signal pathways for implementation in evaluating the immunotherapy responses and clinical outcomes of BC subtypes.

In this study, we identified lncRNAs that are highly related to BC prognosis through 10 critical tumor- signaling pathways, after which we established a novel prognostic signature entitled OncoSig. We demonstrated the OncoSig as a predictor of prognosis and immunotherapy response, which is composed of 29 lncRNAs that are highly correlated with biological characteristics, immune features, overall survival (OS), gene mutation, and so on. Additionally, the pan-cancer analysis revealed that OncoSig is significantly related to the prognosis of 21 cancer types, indicating its reliability and clinical value.

## Materials and methods

### Data source and preprocessing

The basic clinical and RNA sequencing (RNA-seq) data (RNA SeqV2) of 33 cancers were retrieved from The Cancer Genome Atlas (TCGA) database using the Bioconductor package *TCGAbiolinks* (version: 2.20.0) (24), where expression data were normalized by FPKM (fragments per kilobase of exon model per million mapped fragments) and then transformed using log_2_(FPKM + 1). Mutation data for breast-invasive carcinoma (BRCA, also noted as TCGA-BRCA) patients were also obtained from the TCGA using *TCGAbiolinks*. In addition, four microarray datasets of BC were available from the Gene Expression Omnibus [accession numbers: GSE21653 (25), GSE20685 (26), GSE31448 (27), and GSE103091 (28, 29)], and their expression was corrected and normalized using the robust multiarray averaging (*RMA*) procedure (30).

The TCGA-BRCA dataset was used to establish the OncoSig prognostic signature for BC patients, and four independent microarray datasets were employed to assess the performance of this signature. Datasets from TCGA for the other 32 cancers were used to explore the broader prognostic performance of the OncoSig in pan-cancer. The details of these datasets mentioned above can also be found in **Supplementary Table S1**.

### Identification of oncogenic signaling pathway-related long non-coding RNAs

**Figure 1:**
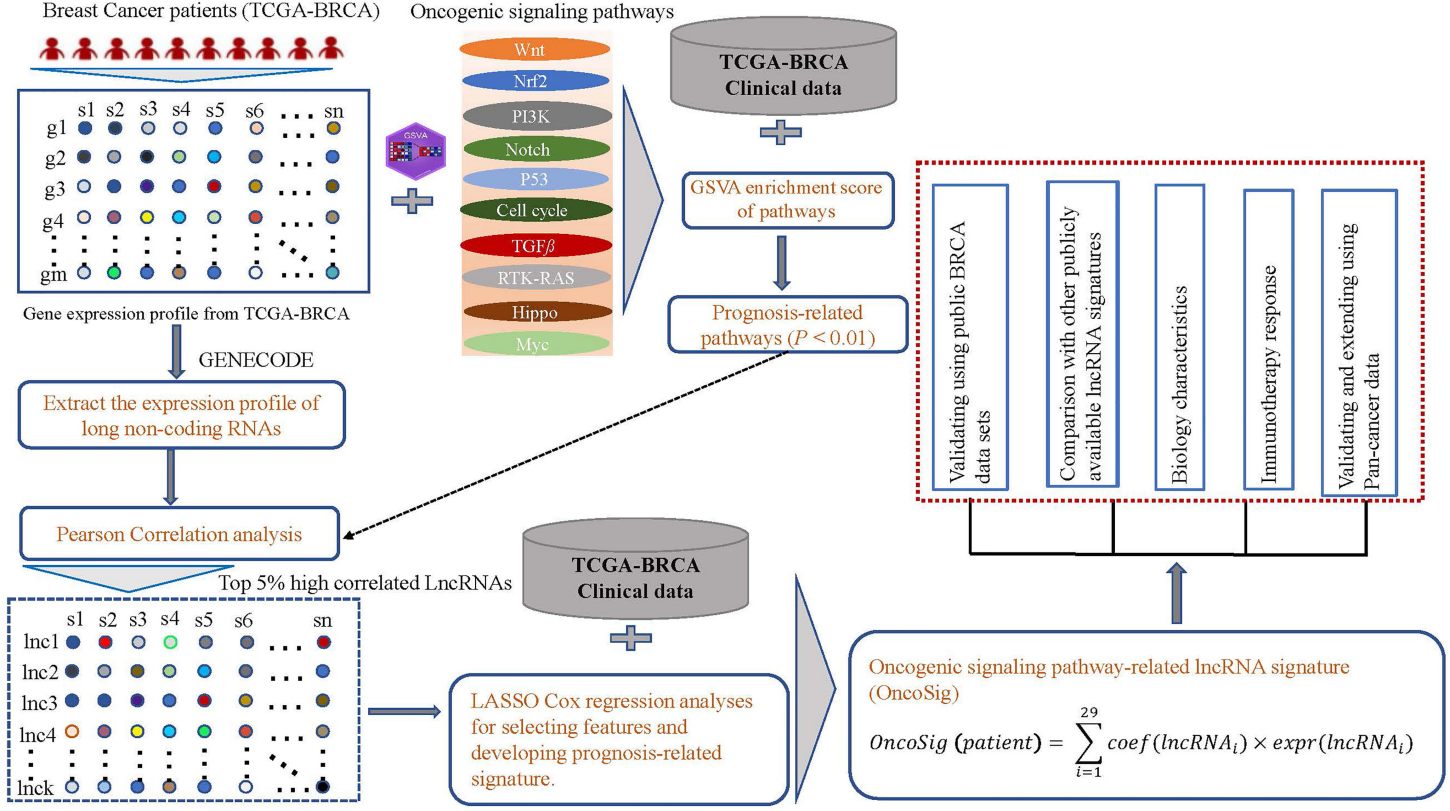
Analysis overview. Transcriptomic, mutational, and clinical data for breast cancer (BC) patients were collected from The Cancer Genome Atlas (TCGA) database. The GSVA tool was used to assess the enrichment scores (ESs) of 10 oncogenic signaling pathways in each patient. Long non-coding RNAs (lncRNAs) were extracted based on the annotation information in GENECODE, and the correlation coefficients between the lncRNA levels and ESs of each prognostically relevant pathway were calculated. Only the top 5% of relevant lncRNAs were retained for each oncogenic signaling pathway and further combined with clinical data to screen for lncRNAs significantly associated with prognosis, resulting in the retention of 29 lncRNAs. The weighting coefficients for these lncRNAs were estimated using multivariate Cox regression to generate the OncoSig signature. Lastly, BC and pan-cancer cohorts were used to assess the potential biological characteristics of the signatures as well as their broader clinical value.

By matching the RNA-seq expression profiles of genes and the annotation file GENCODE (version 25), we extracted genes annotated as “long non-coding RNAs” from the GENCODE project. The lncRNAs starting with “MT-” and “RP” were filtered out, and 3,006 unique lncRNAs were retained. To identify oncogenic signaling pathway–related lncRNAs, we first exclusively wielded the microarray model of the R package *GSVA* (version 3.48.1) (31) to assess the activity [also called the enrichment score (ES)] of 10 oncogenic signaling pathways for each patient in the TCGA-BRCA cohort. These 10 pathways are Receptor Tyrosine Kinase (RTK)-RAS, Notch, Hippo, *β*-catenin/Wnt (Wnt), PI-3-Kinase/Akt (PI3K), Cell cycle, TGFb Transforning growth factor beta (TGFβ), Myc, P53, and Nrf2; their corresponding genes can be collected from a previous study (32). Then, for each pathway, the correlation between lncRNA expression and the ES for each pathway was calculated, and the top 5% was retained. Finally, 498 unique lncRNAs were obtained (see **Figure 1**).

### Development of oncogenic signaling pathway–related long non-coding RNA signatures

A total of 1,109 BC patients (only tumor samples) were randomly allocated to the training group (n = 554) and testing group (n = 555). For the training group, lncRNAs significantly associated with OS were filtered out from 498 unique lncRNAs through the univariate Cox regression analysis in combination with clinical data. Further, relying on the least absolute shrinkage and selection operator (LASSO) Cox regression model (R package, *glmnet*), resulted in the retention of 29 lncRNAs (see **Table S2**). Finally, a prognostic signature was proposed as a linear combination between the retained lncRNA expression values and their weights, which were derived using multivariate Cox regression (see **Figure 1** and the following equation). The testing group was initially used to assess the prognostic performance of the signatures generated from these 29 lncRNAs.


$$\text{OncoSig }\left(\text{patient}\right)=\;\sum_{i=1}^{29}coef\left(lncRNA_{i}\right)\times expr\left(lncRNA_{i}\right)$$

Additionally, to improve the robustness of the signature, the training and testing groups (i.e., the TCGA-BRCA cohort) were merged to produce the final coefficients of these lncRNAs, as listed in **Table S2**.

### Kaplan–Meier survival curve

KM survival curves were combined with the log-rank tests to assess whether the different risk groups (e.g., high- and low-risk groups determined by the OncoSig) demonstrated significantly different patterns of survival (*surv cutpoint* function, R package *survminer*, version 0.4.2), with their survival curves being considered as having a significant statistical difference when the *p*-value ≤0.05.

### Differentially expressed genes associated with groups depicted by the OncoSig

The identification of DEGs between the groups (i.e., high- and low-risk groups) depicted by the OncoSig involved two steps: 1) assessing differences between the groups using the *limma* package (version, 3.48.1) (33) and 2) with |log2FC| ≥ 1 and adjusted *p*-value ≤ 0.01 [Benjamini–Hochberg method (34)] as the filtering conditions. The genes that met both these conditions were considered significantly different.

### Functional enrichment analysis and gene set enrichment analysis

The gene annotation enrichment analysis of DEGS between the groups, as depicted by the OncoSig, was used to derive statistically different gene ontology (GO) terms. It was performed using the R Bioconductor package *clusterProfiler* (version 4.0.2) (35), where GO terms with the Benjamini–Hochberg (27) adjusted at *p*-value ≤0.01 were considered significantly different.

To evaluate the infiltration of immune cells, a compendium of 782 marker genes related to 28 tumor-infiltrating immune cell types was obtained from the study by Charoentong P et al. (36). Again, gene set enrichment analysis (GSEA) was performed on the marker gene sets of these immune cells using the Bioconductor package *clusterProfiler* (version 4.0.2).

### Cellular infiltration estimation

The stromal, immune, and tumor purity scores were assessed for each patient using the ESTIMATE algorithm (R package *estimate*, version 1.0.13) (37).

The relative fraction of 22 immune cell types for each patient was estimated using CIBERSORT (https://cibersort.stanford.edu/), where the signature gene expression profile (also called the base matrix) was LM22 (38).

### Immunophenoscore calculation

The immunophenoscore (IPS) quantifies four different immune phenotypes, including antigen presentation, effector cells, suppressor cells, and checkpoint markers, using several immune responses or immune toleration markers provided in a previous study (36). A higher z-score of IPS summarizing these four categories indicates a more immunogenic sample (36, 39).

### Performance comparison among different breast cancer prognostic signatures

To compare the performance of the prognostic signatures, a concordance index (C-index)—which reflects the probability of agreement between the predicted results and the actual observed value—was employed. Three steps were adopted: (1) 200 patients were randomly chosen without replacement from the TCGA-BRCA cohort; (2) the C-index corresponding to each BC signature was calculated separately using the *coxph* function (R package, *survival*, version: 3.3-1); and (3) the above steps were repeated 100 times and, subsequently, the distribution of the C-index for each signature was summarized.

### Statistical analysis

Hierarchical cluster analyses were performed using Euclidean distances and the complete linkage method, while correlations between the gene expression of lncRNAs were calculated using the *Pearson* method. The significance of differences between the two groups was obtained using the Wilcoxon’s test function. Additionally, a linear regression model was used to meet the trend of the scattered points. The time-dependent receiver operating characteristic (ROC) curve evaluated the performance of the OncoSig. Furthermore, a multivariate Cox regression was carried out to assess the independence of the OncoSig from other key clinical factors, including age, PAM50 subtype, and tumor grade. Since the PAM50 typing of BC was not available in the testing microarray datasets (see **Table S1**), it was predicted using the *genefu* package (40) (version: 2.24.2). To systematically understand the prognostic value of the OncoSig in the different BC cohorts, a prognostic meta-analysis was performed by deploying a fixed effects model that used the R package *meta* (version 5.2-0). All statistical analyses were implemented using the tool R project for statistical computing (version 4.1.0). The *P*-values (Wilcoxon’s test, Fisher’s exact test, and Student’s *t*-test) when comparing the groups were two sided, with ∗ *p ≤*0.05 considered as statistically significant.

## Results

### Identification of Oncogenic Signaling Pathway– and Prognosis–Associated Long Non-Coding RNAs

**Figure 2:**
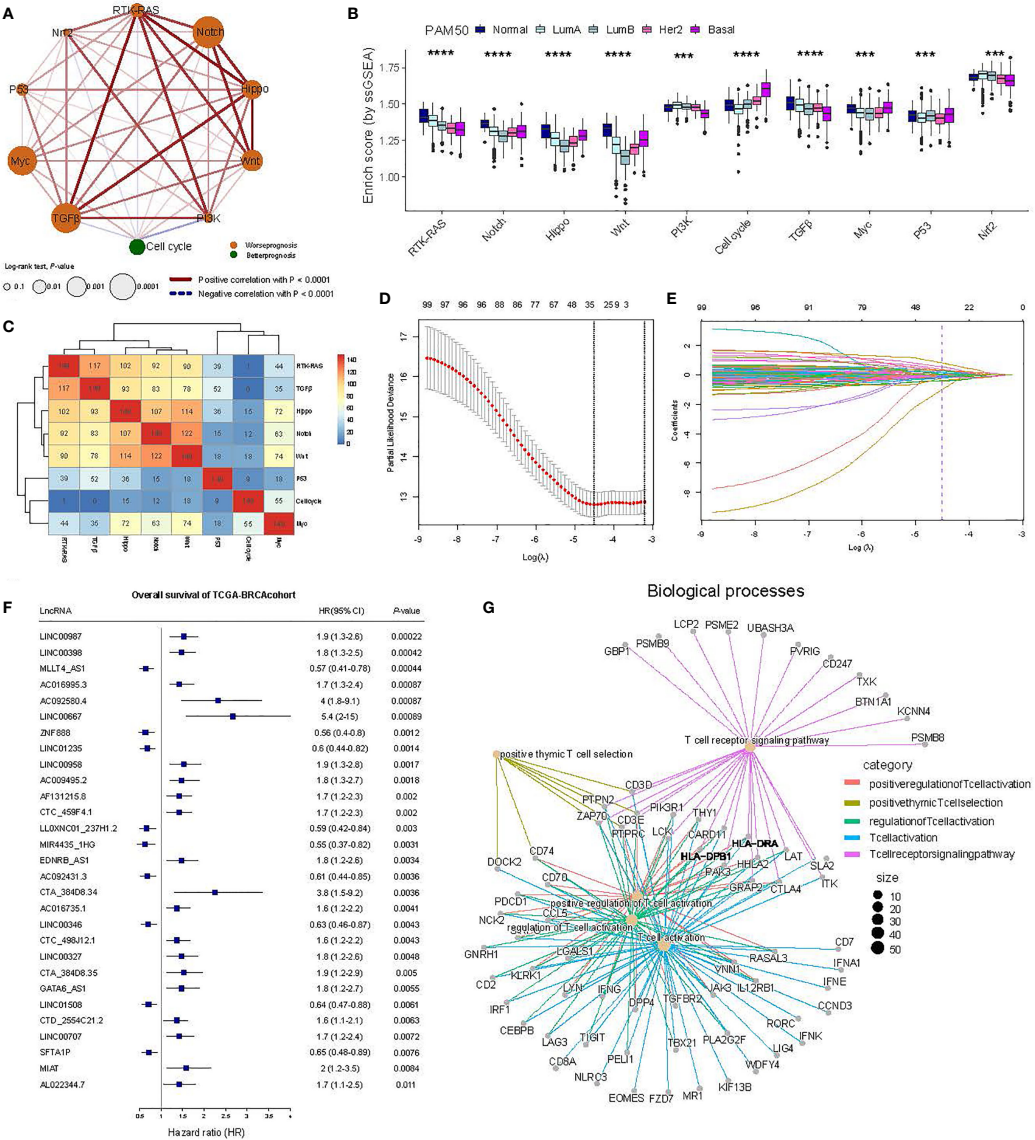
Identification of oncogenic signaling pathway-associated lncRNAs using TCGA-BRCA transcriptome data. **(A)** Interaction of 10 oncogenic signaling pathways. The size of the circles represents the prognostic effect of each cell type, while the color of the fill is scaled by the P-value. Red and blue colors indicate positive and negative correlations, respectively. **(B)** A comparison of the ESs of the oncogenic signaling pathways in the PAM50 subtypes. The Wilcoxon rank-sum test was used for statistical analysis. ***p ≤ 0.001, ****p ≤ 0.0001. **(C)** Heat map showing the number of overlaps between lncRNAs highly associated with specific oncogenic signaling pathways and other pathways using hierarchical clustering. The top 5% of the lncRNAs associated with specific oncogenic signaling pathways were selected. The upper bound of the color bar is 150. **(D)** The least absolute shrinkage and selection operator (LASSO) regression model revealed partial likelihood deviance in the 10-fold cross-validation. **(E)** The LASSO coefficient profiles of prognosis-related lncRNAs in 10-fold cross-validation. **(F)** The forest plot of 29 candidate prognosis-related lncRNAs associated with OS in the TCGA-BRCA cohort. **(G)** Gene ontology (GO) functional enrichment analysis for mRNAs with coexpressed lncRNAs.

Ten crucial signaling pathways have been identified as influencing cancer progression—Cell cycle, Hippo, Myc, Notch, Nrf2, PI3K, RTK-RAS, TGF*β*, P53, and *β*-catenin/Wnt (Wnt) (32). To characterize the impact of these pathways on BC, we first fetched BC samples from the TCGA database and, subsequently, calculated the activity of each pathway in each BC patient using the single-sample GSEA method encapsulated in the GSVA package (**Figure 1**, see Materials and Methods) (31). Based on the activity scores and activities of different tumor pathways in the BC cohort, we set up interaction networks between 10 cancer pathways and analyzed their relationship with the survival prognosis. The results showed RKT-RAS, Notch, Hippo, Wnt, and TGFβ as having strong positive correlations that were significantly associated with survival prognosis in BC patients (**Figures 1**, **2A**). Although the MYC pathway was significantly associated with patient prognosis, the positive correlation with the other pathways was weaker (**Figure 2A**). Interestingly, the Cell cycle pathway was negatively correlated with TGFβ, Nrf2, RKT_RAS, and PI3K, and it served as a protective factor for the prognosis of BC patients (**Figure 2A**). At the same time, no significant association was observed between Nrf2, PI3K, and the prognosis of BC patients (**Figure 2A**). Further, we compared the distribution of the activity scores (also called ESs) of each of the 10 pathways across the PAM50 subtypes to identify significant differences in their activities in the different subtypes. On combining the clinical prognosis of PAM50 patients—normal-like over LumA over LumB over HER2 over basal—we observed that the ESs of the RTK-RAS and Cell cycle pathways were most consistent with the PAM50 clinical trend. The RTK-RAS ES was the highest for Normal-like and lowest for Basal. For the Cell cycle pathway, the ESs for LumA activity and Basal were the lowest and the highest, respectively (**Figure 2B**). These results depict the synergistic or antagonistic relationships between 10 tumor-related pathways and their impact on BC patients’ prognoses, revealing that different pathways have different abilities to portray PAM50 subtypes (**Figures 2A, B**). Since the crosstalk between the signaling pathways and lncRNAs impacts cancer progression (17–19), we annotated the genes from the TCGA-BRCA cohort using GENECODE and correlated the expressions of the annotated lncRNAs (3006) with the ESs of eight pathways that exhibited significant association with the prognosis in BC patients, selecting only the top 5% of correlated lncRNAs in each pathway (149 lncRNAs for each pathway). On analyzing the lncRNAs shared among the different pathways, we found a number of them present in RTK-RAS, TGFβ, Hippo, Notch, and Wnt, whereas P53, Cell cycle, and Myc shared fewer lncRNAs with the other pathways, indicating solid specificity. Following this, the hierarchical clustering also showed that there are two different functional pathway modules, implying that their status and role in tumor progression may be quite different (**Figure 2C**).

To identify the pivotal lncRNAs that were highly associated with the prognosis of BC patients, we combined the clinical data and screened the lncRNAs mentioned above using LASSO-Cox regression (**Figures 1**, **2D, E**), which yielded 29 lncRNAs (**Figure 2F**). Furthermore, to analyze the biological processes in which these lncRNAs may be involved, we conducted a coexpression analysis of the lncRNAs with mRNAs. For each lncRNA, only the top 50 mRNAs with the highest coexpression correlation were retained. Annotating the GO of these mRNAs using the R package *clusterProfiler* (35) revealed that they are mainly involved in T-cell activation, regulation, and differentiation processes (**Figure 2G**). These results suggest that 29 lncRNAs are involved in tumor-associated pathways. Their altered expression may affect normal gene damage repair pathways by disrupting the balance of lncRNA-associated regulatory networks, thereby affecting the stable regulation of important pathways.

### Proposing a Novel Long Non-Coding RNA Signature for the Clinical Stratification of Breast Cancer Patients

**Figure 3:**
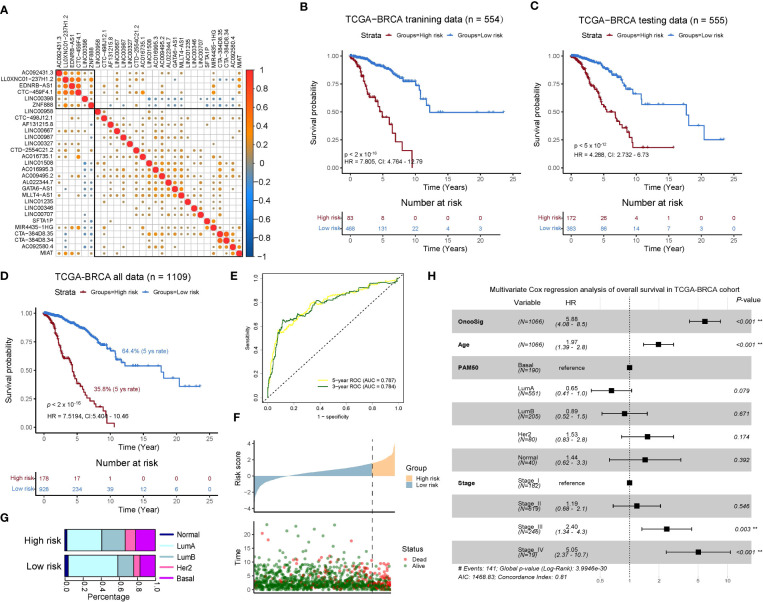
Development and validation of an oncogenic signaling pathway–derived lncRNA signature for outcome prediction in the TCGA-BRCA cohort. **(A)** Pearson correlation analysis for 29 lncRNA expressions divided into two submodules based on hierarchical clustering. **(B–D)** Kaplan–Meier (KM) survival analysis for the overall survival (OS) curves of BC patients in training **(B)**, testing **(C)**, and the total set. **(E)** Time-dependent receiver operating characteristic (*ROC*) curve at 3 and 5 years of OS. **(F)** High- and low-risk groups depicted by the OncoSig, ordered according to their scores, presenting the corresponding survival status for the BC patients. **(G)** Composition of patients with the PAM50 subtype in the two groups. **(H)** A multivariate analysis of the OncoSig, age, PAM50, and stage with OS in the TCGA-BRCA cohort.

To explore the potential prognostic roles of the selected 29 lncRNAs associated with oncogenic signaling pathways in clinical diagnosis, we first measured the correlations between their expressions, which showed that only a few of the lncRNAs had a relatively strong positive/negative correlation with each other. This finding indicated that these 29 lncRNAs had a low degree of colinearity and good independence that could help to provide a comprehensive picture of their impact on the prognosis of BC patients (**Figure 3A**). Next, we divided the TCGA-BRCA cohort into a training set (n = 554) and testing set (n = 555) using random sampling without replacement. For the training set, we coupled the expression data of the lncRNAs with clinical characteristics and used multivariate Cox regression to obtain 29 coefficients that characterize the degree of prognostic impact of the lncRNAs. The linear sum of these coefficients multiplied by the expression of the 29 lncRNAs was indicated as OncoSig. The training and testing sets were split according to their higher and lower OncoSig scores, respectively, using optimal cut points determined by the *surv_cutpoint* method (see Materials and Methods), whereby significant differences (*p*-value ≤ 0.01) in OS were noted for both groups (**Figures 3B, C**), indicating that the novel OncoSig signature successfully portrayed the prognosis of BC patients. To adjust for the coefficients obtained previously, we merged the training and testing sets and reused the multivariate Cox regression to acquire a new OncoSig that better described the prognosis of BC patients. The 5-year survival rate for the low-risk score group of BC patients was 64.4%, which is significantly higher than the high-risk group (35.8%) (**Figure 3D**). For this reason, we used the adjusted OncoSig as the final signature of the 29 lncRNAs (see **Table S2**).

The area under the curve (AUC) of the signature was used to further analyze the prognostic performance of the OncoSig, presenting 0.787 and 0.784 at 5 and 3 years of OS, respectively (**Figure 3E**). We also found that a higher-scoring OncoSig group meant shorter survival time and signified more deaths (**Figure 3F**). In addition, we investigated the composition of the PAM50 patients in both groups stratified by the OncoSig to find that Basal and HER2 patients dominated the high-risk group, while Normal-like and LumA patients were in the low-risk group (**Figure 3G**). Subsequently, a new question that arises is whether OncoSig can be applied as a valid and independent prognostic indicator in the BC cohort. For this purpose, multivariate Cox regression analysis was employed using covariates such as OncoSig, age, PAM50, and stage. The results demonstrated that OncoSig, age, and stage III and IV were significantly associated with prognosis in BC patients, with OncoSig exhibiting the worst HR value, hinting that it could certainly be the most crucial risk factor compared to these key clinical characteristics (**Figure 3H**). In summary, this evidence indicates that the higher the OncoSig score, the worse the prognosis and vice versa. This result also establishes OncoSig as an independent prognostic factor that contributes to clinical diagnosis and research.

### Evaluating and comparing the prognostic performance of the OncoSig

**Figure 4:**
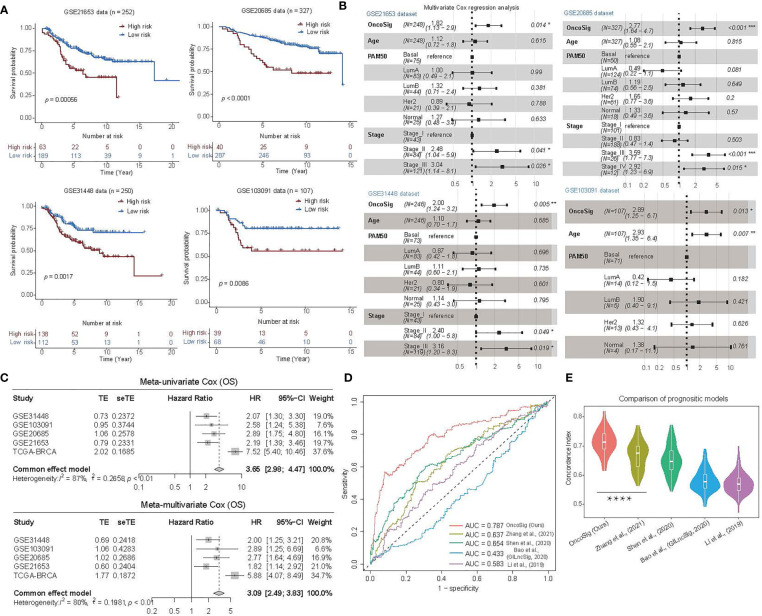
Prognostic performance of the OncoSig on four independent BC testing cohorts. **(A)** KM survival analysis for the OS curves of BC patients in GSE21653, GSE20685, GSE31448, and GSE103091, respectively. **(B)** Multivariate analysis of the OncoSig, age, PAM50, and stage with OS using the four testing cohorts, respectively. **(C)** Meta-analysis performed on the prognostic value of the OncoSig for patients in the five cohorts, using a fixed effects model to calculate pooled HR values (top: meta-univariate; bottom: meta-multivariate). **(D)** The ROC analysis of 5-year OS of the OncoSig versus other published prognostic signatures for BC. **(E)** Comparison of the performance of five BC signatures using the concordance index (C-index), randomly permuted 100 times, with 200 patients randomly selected from TCGA-BRCA each time, and the C-index calculated separately for each signature. The Wilcoxon rank-sum test was used for the statistical analysis. ****p ≤ 0.0001.

To estimate the prognostic value of the OncoSig more broadly, we obtained four independent gene expression datasets of BC from the GEO database —GSE21653 (25), GSE20685 (26), GSE31448 (27), and GSE103091 (28, 29)—each with its gene expression signal backgrounds corrected and quantiles normalized using the *RMA* (30) package in R (see Materials and Methods) and then calculated their OncoSig scores. The results demonstrated that both the high- and low-risk groups portrayed by the OncoSig were significantly different in each of these independent test sets (*p* ≤ 0.05), consistent with the trend reflected in the TCGA-BRCA cohort (**Figure 4A**). In addition, we needed to explore whether the OncoSig remained available as an independent prognostic factor in the four testing cohorts. For this purpose, a multiple Cox regression strategy was applied, where the included covariates were age (i.e., ≥ 60 or not), PAM50 typing, and tumor grade. It should be noted that, as the clinical data in the GSE20685 and GSE103091 datasets did not contain the PAM50 subtypes, the *genefu* (40) package in R was used to make separate predictions for each sample in these two datasets. In addition, as the clinical data for GSE103091 did not include the tumor grade, this covariate was omitted in the multiple Cox regression analysis. The results revealed that OncoSig was statistically significant in all the testing cohorts (*p* ≤ 0.05) (**Figure 4B**; **Table S1**), further suggesting that the signature can be regarded as an independent prognostic factor in BC. Moreover, a prognostic meta-analysis was conducted to examine the comprehensive prognostic value of all five BC cohorts, signifying that a high OncoSig score indicated a significant risk factor for the OS of BC patients (**Figure 4C**). As a whole, these results denoted that the OncoSig could be a potential attribute of BC patients and may be of great value for clinical prognostic assessment.

To our knowledge, several prognostic signatures for BC constructed using lncRNAs have been reported. These include the five-lncRNA signature proposed by Li et al. (41), the signature GILncSig using lncRNAs highly associated with genomic instability constructed by Bao et al. (42), a signature consisting of 11 lncRNAs identified by Shen et al. (22) on comparing differences between high and low immune infiltration groups in BC cohorts, and a signature constructed on 9 lncRNAs derived from autophagy-associated lncRNAs by Zhang et al. (43). To compare the prognostic performance of the OncoSig with the other lncRNA signatures for BC, we calculated the risk score for each sample in the TCGA-BRCA cohort, utilizing each of the four signatures mentioned above. As shown in **Figure 4D**, the OncoSig predictions attributed the highest AUC to the five-year OS, followed by the nine-lncRNA signature constructed by Zhang et al., while the lowest AUC was adjudged to the five-lncRNA signature constructed by Li et al. (**Figure 4D**). Since the C-index responds to the probability that the predicted outcome is consistent with the actual observed, we further randomly sampled the TCGA-BRCA cohort to calculate the C-index corresponding to the optimal stratification of each signature risk score and repeated this activity 100 times (see Materials and Methods). The results showed that the OncoSig has the highest average C-index and is significantly different from Zhang et al. (**Figure 4E**). Indeed, these results suggest that the OncoSig has a better prognostic performance compared to the other lncRNA signatures for BC.

### Clinical and biological landscape of the two groups, as described by the OncoSig

**Figure 5:**
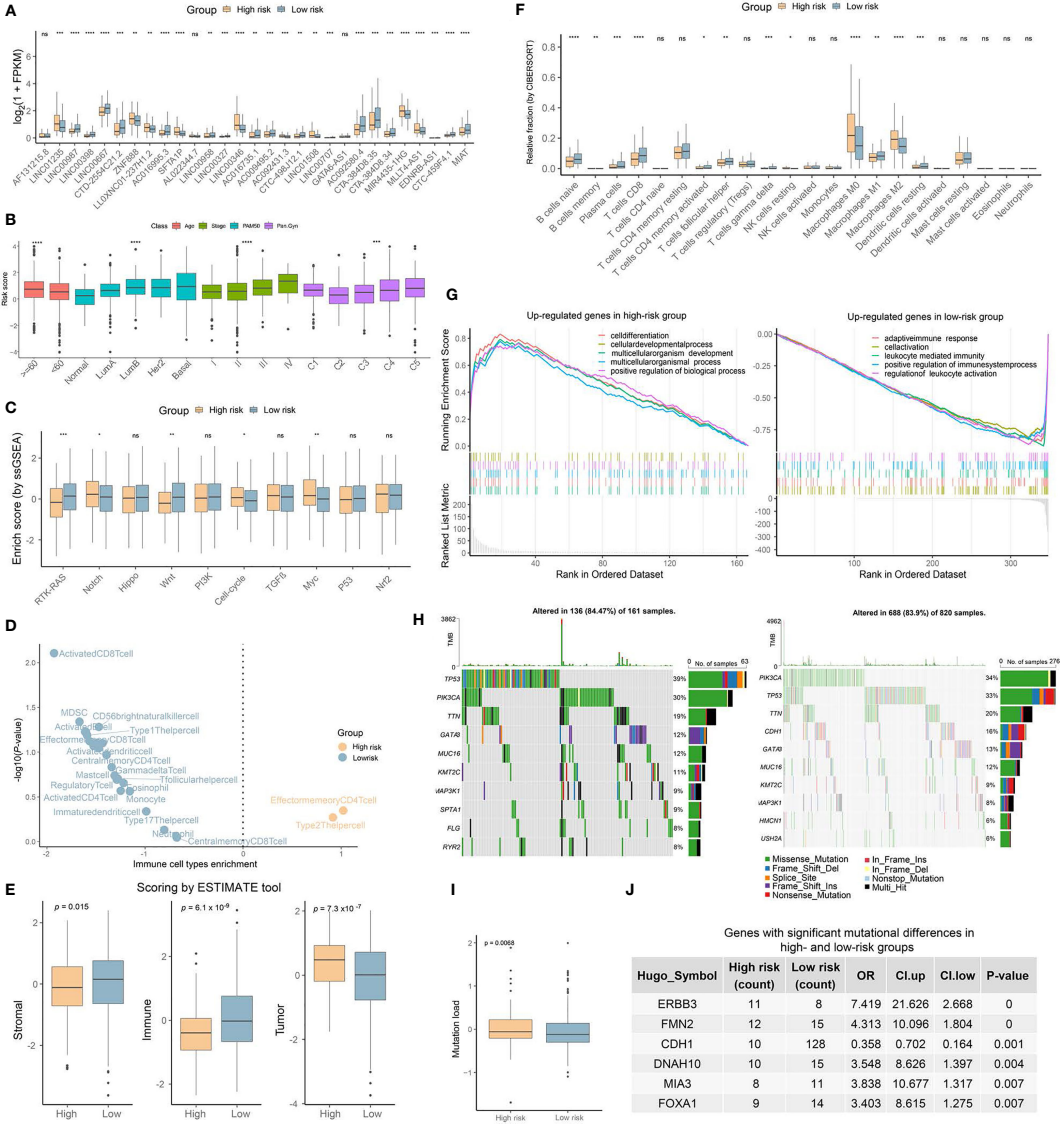
Clinical and biological characteristics of stratification results of the OncoSig. **(A)** Box plot showing the statistical difference of the 29 lncRNA expressions between high- and low-risk groups. **(B)** Clinical characteristics of the risk score obtained on a stratified analysis of the TCGA-BRCA cohort. **(C)** Box plot showing the statistical difference in the ES of the oncogenic signaling pathways between high- and low-risk groups. **(D)** Volcano plot for the enrichment of immune cell types in tumors with high and low OncoSig scores, calculated based on the normalized enrichment score from the gene set enrichment analysis (GSEA). **(E)** Stromal, immune, and tumor scores from the ESTIMATE tool for the high- and low-risk groups. **(F)** CIBERSORT predictions of the infiltration levels of 22 immune cells in the low- and high-risk groups. The dots represent the immune cell–scaled expression values. **(G)** GSEA showing significantly enriched GO terms in low- and high-risk groups, respectively. **(H)** The tumor mutational burden difference between the low- and high-risk groups. **(I)** Comparison of the relative distribution of the mutation load between the low- and high-risk groups in the TCGA-BRCA cohort. **(J)** Table showing the significantly mutated genes between the low- and high-risk groups. Only genes with more than 10 mutations were included in the Fisher’s exact test analysis. Notably, ns: not significant, *p ≤ 0.05, **p ≤ 0.01, ***p ≤ 0.001, ****p ≤ 0.0001. The Wilcoxon rank-sum test was used for the statistical analysis.

By comparing the expression levels of 29 lncRNAs in the high- and low-risk groups, we found that all but three—*AF131215*.8, *AL022344.7*, and *GATA6-AS1—*exhibited significant differences (Wilcoxon test, *p*-value ≤ 0.01) (**Figure 5A**). The relationship between the OncoSig scores and clinical characteristics, which included age (≥60 or not), stage, PAM50, and Pan-Gyn clusters, were further examined in the TCGA-BRCA cohort to reveal significant differences (**Figure 5B**). Specifically, Lumina A, the least- aggressive subtype of the PAM50, presented the lowest risk score while the HER2 and Basal types were the most aggressive with the highest risk scores (**Figure 5B**). We also observed a positive concordance between pathological staging and the OncoSig score in BC patients, with a similar trend in the Pan-Gyn C1 to C5 clusters (**Figure 5B**). In addition, differences in the ES scores of 10 oncogenic pathways obtained using the *GSVA* (31) tool for the two groups depicted by the OncoSig were investigated, where RTK-RAS, Wnt, and Myc showed significant differences, while the other pathways showed none (Wilcoxon’s test, *p*-value ≤ 0.01) (**Figure 5C**).

Next, the infiltration of 28 immune cell types gathered from the previous study (36) of the two groups depicted by the OncoSig for BC patients was assessed using the GSEA toolkit. It was observed that these groups revealed distinct patterns of immune infiltration, with patients in the low-risk group enriched by an absolute predominance of immune subpopulations (**Figure 5D**, see Materials and Methods). ESTIMATE (37) was performed to estimate and compare the stromal, immune, and tumor scores in both risk groups, all of whom showed significant differences, with immune showing the most significant difference (**Figure 5E**). To refine the differences between immune infiltrations in the high- and low-risk groups, we estimated the relative fraction of 22 immune cells for each patient in both groups by using CIBERSORT coupled with the base matrix “LM22,” wherein “B-cell naïve,” “T-cell CD8,” “macrophage M0,” and “macrophage M2” demonstrated the most significant differences (**Figure 5F**; see Materials and Methods). Further, differentially expressed genes (DEGs) in the high- and low-risk groups were identified. The GO annotation of these DEGs indicated that the high-risk group was enriched in biological processes, such as cell development and differentiation, while the low-risk group was more involved in biological processes, such as immune response and regulation (**Figure 5G**). Overall, these results adequately revealed a profound association between OncoSig and immune infiltration, with lower OncoSig scores associated with higher levels of immune infiltration and vice versa (**Figures 5D–G**).

To further investigate the heterogeneity of single-nucleotide polymorphisms in the risk groups depicted by the OncoSig, we retrieved a dataset of mutations corresponding to BC patients from the TCGA database. As revealed in **Figure 5H**, the top 10 genes with the highest mutation frequencies in each group were presented separately, with alterations occurring in 136 of the 161 samples (84.47%) in the high-risk group and in 688 of the 802 samples (83.9%) in the low-risk group. Notably, *TP53*, *SPTA1*, *FLG*, and *RYR2* accounted for 39%, 9%, 8%, and 8% of the mutation frequency in the high-risk group, respectively, while *PIK3CA* (34%) and *CDH1* (16%) were more prominent in the low-risk group (**Figure 5H**). The mutational burden was also investigated, showing significant differences between the groups portrayed by the OncoSig (**Figure 5I**). Lastly, genes with significant mutational differences between the two groups were examined, with *ERBB3*, *FMN2*, *DNAH10*, *MIA3*, and *FOXA1* observed to be the frequently mutating genes in the high-risk group, while *CDH1* was found to be enriched in the low-risk group (**Figure 5J**). Effectively, the high- and low-risk groups depicted by the OncoSig revealed significant heterogeneity in their clinical characteristics and biological mechanisms, suggesting the potential value of the OncoSig as a clinical signature to predict the prognosis of BC patients.

### Potential of the OncoSig as an Indicator of Immunotherapy Response in Breast Cancer

**Figure 6:**
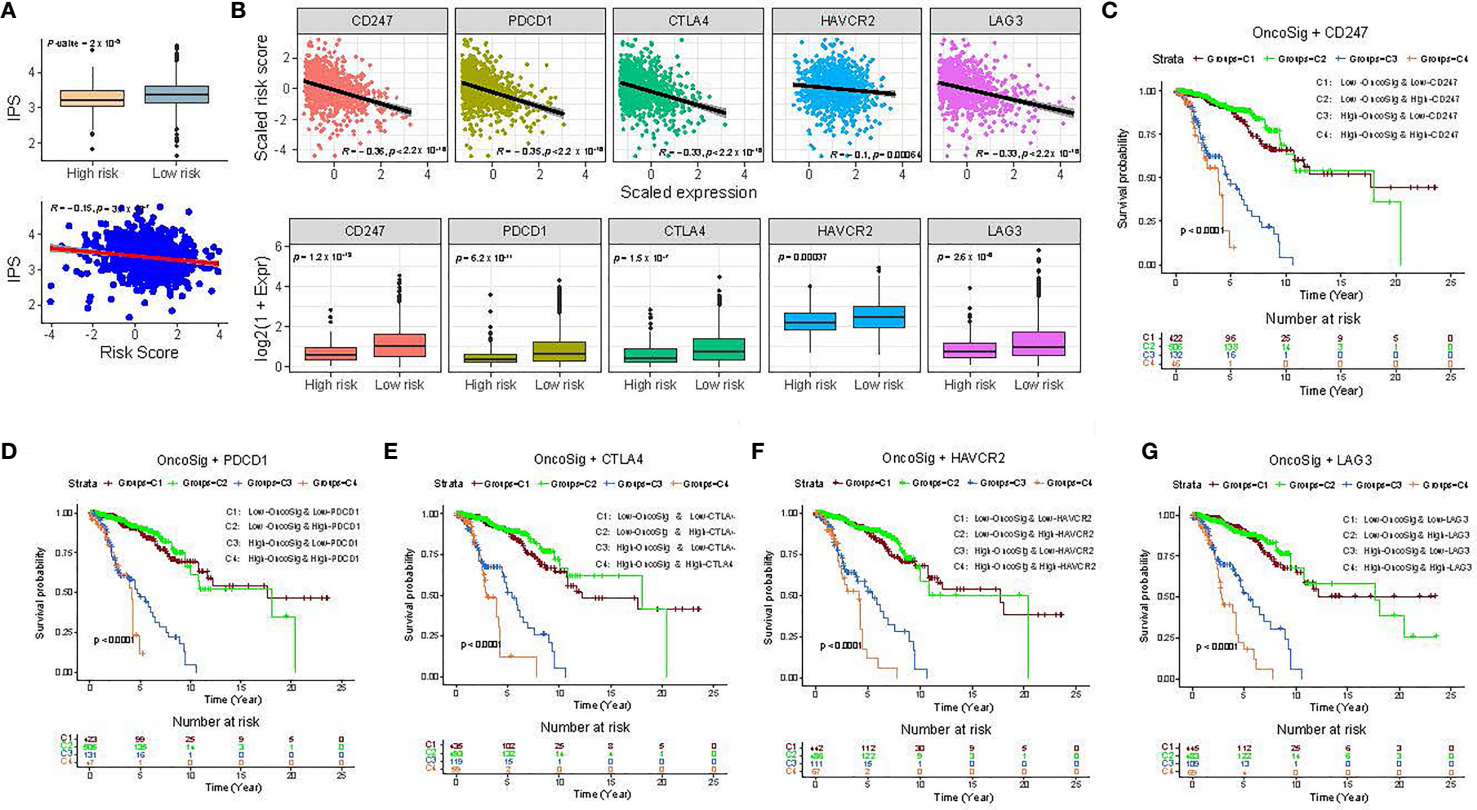
Impact of immune checkpoint gene expression and OncoSig on clinical outcome. **(A)** Comparison of the relative distribution of immunophenoscores (IPSs) between low- and high-risk groups in the TCGA-BRCA cohort (top). The scatter plot shows the correlation between the correlated IPS score and the OncoSig score, while *R* indicates the Pearson correlation coefficient (bottom). **(B)** Comparison of the expression pattern of immune checkpoint **(ICI)** genes between patients with higher and lower OncoSig scores in the TCGA-BRCA cohort. **(C-G)** KM OS curves for the four groups stratified by the OncoSig and *CD247* **(C)**, *PDCD1* **(D)**, *CTLA4* **(E)**, *HAVCR2* **(F)**, and *LAG3* **(G)**. The Wilcoxon rank-sum test was used for the statistical analysis.

To explore the potential of the OncoSig as an indicator of response to immunotherapy in BC patients, the IPS, which refers to an arbitrary 0–10 score based on the sum of weighted average Z-scores for antigen presentation, effector cell, suppressor cell, and checkpoint markers (see Materials and Methods), was calculated. The results displayed that the IPS was negatively connected with the OncoSig scores and also revealed a substantial difference between the high- and low-risk groups, with the latter being more immunogenic (**Figure 6A**). A recent study (44) has already elaborated that immune checkpoint inhibitor (ICI) genes are of significant value in depicting tumor progression. To further inquire into the complicated interactions between OncoSig and ICI genes, the expression patterns of ICI genes—including *CD247* (*PD-L1*), *PDCD1*, *CTLA4*, *HAVCR2*, and *LAG3*—in the different patient groups stratified by the OncoSig were analyzed. The results revealed that the OncoSig had a significant negative correlation with the expression levels of ICI genes (**Figure 6B**). Additionally, in the TCGA-BRCA cohort, patients with low OncoSig exhibited high levels of ICI gene expression compared to those with high OncoSig (**Figure 6B**). This trend is consistent with previous observations specifying that a high expression of immune checkpoint genes is associated with a good outcome (45). It is still unclear whether the OncoSig can reflect clinical results more sensitively, with similar expression levels of ICI genes as above. To clarify this confusion, the BC cohort was divided into four groups using the stratification depicted by the OncoSig into high/low (median values as cutoff points) expression levels of each ICI gene. After this, the survival patterns of these four groups were compared. The results demonstrated that patients with low OncoSig and high expression of ICI genes had the best prognostic performance, while those with high OncoSig and low expression of ICI genes had the worst prognosis (log-rank test, *p*-value ≤ 0.001) (**Figures 6C–G**). In particular, for high OncoSig patients, stratified ICI gene expressions, that is, C3 and C4, resulted in significant differences in survival. However, on stratifying the cohort using ICI gene expression based on the low OncoSig patients, that is, C1 and C2, no significant survival differences were observed (**Figures 6E–G**). Accordingly, these results implied that the OncoSig was closely associated with ICI immunotherapy response and, thus, could be a potential predictive signature for BC patients.

### Extending the effectiveness and clinical value of the OncoSig using Pan-Cancer RNA-Seq Data

**Figure 7:**
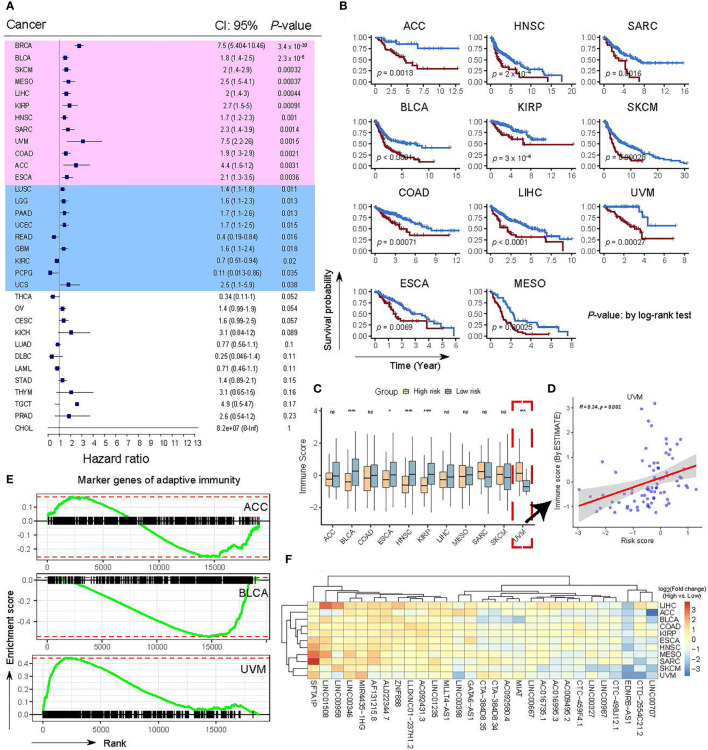
Confirmation of the OncoSig using TCGA pan-cancer datasets. **(A)** Validation of the impact of the OncoSig on survival using TCGA pan-cancer datasets. Background colors in pink indicate cancer types with *p*-values less than 0.01, while light blue indicates cancer types with *p*-values less than 0.05. *P*-values were obtained using a log-rank test. **(B)** KM OS curves between patients with a higher and a lower OncoSig score in 11 cancer types. **(C)** Comparison of the relative distribution of immune scores derived from ESTIMATE between high- and low-risk groups in 11 cancer types. **(D)** Scatter plot showing the correlation between the correlated immune score and the OncoSig score. *R* indicates the Pearson correlation coefficient. **(E)** GSEA for 431 marker genes of adaptive immunity reveals the relationship between low- and high-risk score groups in adrenocortical carcinoma, bladder urothelial carcinoma, and uveal melanoma cancer types. **(F)** Heat map with hierarchical clustering showing the expression of 29 lncRNAs between low- and high-risk score groups. Red and blue represent high and low expressions, respectively. The Wilcoxon rank-sum test was used for the statistical analysis. ns: not significant, *p ≤ 0.05, ***p ≤ 0.001, ****p ≤ 0.0001.

To investigate the effectiveness and clinical values of the OncoSig on other cancers, we obtained transcriptome expression datasets coupled with clinical data from the TCGA database for 32 other cancer types (the full names and abbreviations of these cancers can be found in **Table S1**) and scored each sample using the OncoSig formula (see Materials and Methods). To assess the prognostic ability of the OncoSig in other cancers, optimal cut points and survival models were used to investigate the survival patterns of the risk groups, with the results showing significant differences between the stratifications depicted by the OncoSig in 21 cancer types (*p*-value ≤ 0.05), wherein 11 cancer types with a *p*-value ≤ 0.01 were identified (excluding BRCA, **Figures 7A, B**). On comparing the immune infiltration, estimated by *ESTIMATE* (37), significant differences were observed between the high- and low-risk groups for each of the 12 cancer types, including bladder urothelial carcinoma (BLCA), head and neck squamous cell carcinoma (HNSC), and kidney renal papillary cell carcinoma (KIRP), with lower immune infiltration in the high-risk group. This is consistent with the results observed in the BRCA (**Figure 7C**). Interestingly, there was also a significant difference between the two groups in terms of uterine uveal melanoma (UVM). However, the high-risk group still displayed higher immune infiltration and vice versa. In contrast to the OncoSig in BRCA, a significant positive correlation was observed when comparing the intrinsic association between the OncoSig score and immune infiltration (**Figure 7D**). The underlying reason for this may be that UVM belongs to the immune “cold” tumor, that is, a low-density immune infiltrate present both inside and outside the tumor (**Figures 7C, D**) (46, 47). In addition, no significant difference was observed in the other tumor types (**Figure 7C**).

Next, to confirm the above results, we shifted our strategy and adopted the GSEA to assess the activity of adaptive immune genes, available from a prior study (36) (see Materials and Methods), in the high- and low-risk groups denoted by the OncoSig. As shown in **Figure 7E**, no significant enrichment was found between the two groups in adrenocortical carcinoma (ACC); however, considerable enrichment was discovered in the low-risk group in BLCA and in the high-risk group in UVM (**>Figure 7E**), which corroborates the observations in **Figures 7C, D**. The above results suggest that the OncoSig still has prognostic potential in a wide range of cancers, but whether this signature is a risk or a protective factor may be profoundly influenced by the immune-hot and -cold feature of the cancer itself. Finally, we examined the panorama of expressions of these 29 lncRNAs in the high- and low-risk groups of 11 cancer types; the hierarchical clustering result revealed that they could be classified into three major categories (**Figure 7F**)—the comparisons of the low-risk group’s highly expressed lncRNAs (such as *SFTA1P* and *LINC00346*), median highly expressed lncRNAs (such as *LINC00398* and *AC009495.2*), and lowly expressed lncRNAs (*EDNRB−AS1* and *LINC00707*) (**Figure 7F**). Despite this, the expression of these lncRNAs still exhibited heterogeneity across cancers (**Figure 7F**). Overall, these results demonstrated that the OncoSig can serve as a potential predictive signature of response to BC treatment and has the potential for pan-cancer application.

## Discussion

BC is a highly prevalent and heterogeneous malignant disease that occurs almost exclusively in women (1, 3). Immunotherapy for BC has been considered an emerging treatment approach in clinical circumstances. However, an evaluation criterion is extremely necessary due to the uncertainty of the response to immunotherapy and prognosis in BC (6). Li et al. discovered that a new prognostic signature consisting of 24 pairs of differentially expressed immune-related lncRNA (DEirlncRNA) had close connection with tumor-infiltrating immune cells and drug susceptibility (48). An analogous study by Shen et al. identified 36 pairs of DEirlncRNA for appraising the response to immunotherapy and predicting the survival status of invasive BC (49). Along the same lines, our observations highlight the importance of lncRNA signatures pertaining to oncogenic signaling pathways. Previous studies have shown that complex interactions and crosstalk among different signaling pathways are highly relevant to the progression of the disease, making a proper understanding of oncogenic alterations, its detailed mechanisms, and the co-occurrence of these pathways essential for the development of new therapeutic approaches to improve patient care (16, 39). Nonetheless, directly and accurately delineating the roles and alterations among crucial signaling pathways in a clinical setting remains a difficult task. LncRNAs have recently been identified to play fundamental roles in regulating the activation of oncogenic signaling pathways and, thus, can be utilized as specific molecular markers to depict alterations in signaling pathways (16). The current study demonstrated a new 29-lncRNA signature (OncoSig) that is applied as an indicator of immunotherapy response and prognosis in BC from the perspective of interactions between genes and related lncRNAs in oncogene signal pathways.

We originally established interaction networks between 10 cancer pathways and analyzed their relationships with the survival prognosis of BC patients in TCGA. The results showed that the RKT-RAS-, Notch-, Hippo-, Wnt-, and TGFβ- signaling pathways were strongly associated with worse survival prognosis, while the Cell cycle pathway was significantly associated with a better survival prognosis. Surprisingly, there was a contradictory result. As Wang et al. noted, an immune signature named immune-related prognostic score (IRPS), which acted as a tumor suppressor, was appraised by the normalized ES (NES). The results of the IRPS subtypes in the NES values of 10 common oncogenic pathways showed that the Hippo-, Notch-, TGF*β*-, and Wnt-related and RAS pathways exhibited pronounced lower NES values in the low IRPS group, while it had a higher value in the high IRPS group, whereas the Cell cycle and PI3K pathways had significantly lower NES in the high IRPS group (45). We speculated on the major reason behind these conflicting findings to conclude that our results reflected the entire TCGA-BRCA data instead of partial data. In the process of data collection, similar to the lncRNA signature of tumor-infiltrating B lymphocytes developed by Zhou et al. in bladder cancer (50), we initially sought RNA-seq data from the TCGA and extracted 3,006 lncRNA expression profiles by matching their lncRNA annotations in GENCODE. Then, based on the critical role of lncRNAs in regulating oncogenic signaling pathways in human cancers (51, 52), we applied association analysis and LASSO feature–selecting strategies to identify 29 lncRNAs that exhibited high correlation with the signaling pathways, thus influencing prognosis considerably. It brought a hepatocellular carcinoma immune-related lncRNA signature into correspondence, which was also screened by deploying LASSO regression analysis (21). Afterward, we equally and randomly allocated the BC patients from the TCGA into the training and testing sets. The high- and low-risk groups were defined according to the optimal cutoff value of the OncoSig, as determined by the *ROC* curve. Next, the KM survival curve was plotted using the log-rank test, which disclosed that the OS of the high-risk group was worse than that of the low-risk group in both the sets as well as the combined set.

Subsequently, we chose four independent GEO datasets and classified them into two groups according to the best threshold value of the OncoSig. To our astonishment, their KM survival curves wholly displayed a higher OncoSig score and worse OS, similar to the trend in the TCGA-BRCA cohort, implying that it could be developed as a prognostic factor. Additionally, the OncoSig remained an independent risk factor in the multivariate Cox regression model, notwithstanding inequivalently variable factors in five separate datasets and in the prognostic meta-analysis. Above all, we concluded that the newly emerging signature (OncoSig) could be widely applicable. Concurrently, according to our calculations, the OncoSig predicted a five-year OS with the highest AUC for the TCGA-BRCA dataset among the five prognostic signatures. At the same time, it also exhibited the highest average C-index, which estimated whether the predicted probability was consistent with the actual observed value in the above signatures. Evidently, there is no doubt that the OncoSig has better prognostic merit than the other lncRNA signatures for BC.

Similar to our approach, Zhang et al. demonstrated a nine-autophagy-related lncRNA signature for evaluating BC prognosis (43). By using the GSEA, patients in the low-risk group were enriched with 19 immune cell subpopulations; however, only 2 were enriched in patients with high risk. This exhibits the same trend as an existing signature of tumor immune infiltration–related lncRNA in non-small cell lung cancer, which showed 4 immune cell subtypes in the high-risk group and 10 in the low-risk groups (44). Oddly, Zhou et al. obtained the opposite result in the NES, with 11 immune subpopulations mainly concentrated in the high-risk patient group, while only 2 were enriched in the low-risk group (50). GSEA, immune infiltration, and gene function enrichment analysis” need to be corrected as “Immune cells infiltration, immune score and IPS score the high- and low-risk groups stratified by the OncoSig. The TME of diverse cancers comprises three main patterns: immune-desert, immune-excluded, and immune-infiltrated/inflamed (53). We further concluded that the high-risk group appeared to have more immune “cool” tumors with less immune cell infiltration, whereas the low-risk group contained more immune “hot” tumors with stronger immune cell infiltration. This outcome aligns with existing studies, which state that TCGA-BRCA patients have two distinct immune landscapes (54).

Following this, to further ensure the reliability of the OncoSig, we compared the tumor mutation burden (TMB) of the two different risk groups identified by the OncoSig. Our study suggested that *ERBB3*, *FMN2*, *DNAH10*, *MIA3*, and *FOXA1* were the frequently mutating genes in the high-risk group, while *CDH1* predominated in the low-risk group, clearly indicating a higher TMB in the former group when compared to the latter. Karn et al. concluded that lower TMB and less genomic heterogeneity are positively connected with better survival of TIL-rich TNBC (55). Their reviews are consistent with our results, albeit with distinct classification criteria. Our study also disclosed that the Basel, LumB, and HER2 subtypes accounted for larger proportions in the high-risk group than the low-risk group; meanwhile, the LumA subtype showed the opposite trend. Furthermore, Li et al. reported that an altered *CDH1* group involving mutations in the LumA subtype group displayed better survival than a non-altered group (56). This could explain our current result, indicating that a higher *CDH1* mutation rate in the low-risk group is associated with better prognosis in comparison to the high-risk group.

In this study, we focused on how a selected lncRNA affects an oncogenic signaling pathway by altering the expression of related genes and further evaluated the relationships of the PAM50 subtypes. In particular, ICI-based immunotherapy has made tremendous progress in the clinical management of BC patients recently. However, the heterogeneity of tumors has compelled the beneficiary group of this therapeutic strategy to remain a minority. Consequently, it is crucial to select patients who are most likely to profit from ICI by preassessing the predictive signatures of their responses to ICI. Although immune checkpoint genes, such as PD1/PD-L1 and *CTLA4*, are currently available biomarkers used in clinical work, they are insufficient independent predictors of ICI response (57, 58). Meanwhile, despite high expression levels of PD-1/PD-L1, response rates to immune checkpoint blockade therapy have remained variable among BC patients. By comparing the survival distribution of BC patients screened by the OncoSig and immune checkpoint gene expression, we demonstrated that the OncoSig enabled better discrimination between patients with similar levels of gene expression, indicating that patients with low OncoSig and high-level immune checkpoint gene expression may experience greater ICI treatment response. Furthermore, the prognosis of patients with similar OncoSig is, to some extent, influenced by the differential expression of immune checkpoint genes. Therefore, we can conclude that the Oncosig, resembling the immunotherapeutic benefit score developed by Wang et al. (59), has a distinctive characteristic of predicting response to immunotherapy.

To further certify the dependability of the OncoSig, we also obtained transcriptome expression datasets from the TCGA database for 32 other cancer types. We exploited its formula to score each sample of these cancers. Several cancers, including BLCA, HNSC, and KIRP, except for UVM, showed better survival and more immune infiltration cells in the low-risk cohort than its high-risk counterpart. For UVM, the potential cause of this phenomenon could be attributed to the disparity in the immune context of UVM, since it belongs to the immune desert category, or the heterogeneity of UVM, as it contains the pro-tumorigenic immune cells (MDSCs, M2 macrophages, Th2 CD4+, etc.) of the TME (7, 8, 53).

There are still a few other concepts that can be considered to improve our study. First, the OncoSig requires the conduction of live tissue specimen molecular analysis in the 5 BC subtypes to verify its practicability. Furthermore, our pan-cancer analysis may be a double-edged sword. Although it proved the credibility of the OncoSig to a certain extent by covering multiple types of cancer, it did not cover all the types. In the future, there is certainly a need to design a clinical trial to evaluate the prognosis and immunotherapy response of BC patients by using the OncoSig.

In conclusion, the present study uncovered a robust signature in BC, termed as OncoSig, which acted as an oncogene and relied on the correlations of oncogenic signaling pathways and lncRNAs. We demonstrated a solid implementation of the OncoSig in the evaluation of the prognosis for BC and several other cancers as well as in the detection of immunotherapy responses, proving that it might be helpful in distinguishing clinical outcomes in patients suffering from BC and several other cancers.

## Data availability statement

The datasets presented in this study can be found in online repositories. The names of the repository/repositories and accession number(s) can be found in the article/**Supplementary Material**.

## Author contributions

MC and YY designed the study. HL and HL did data analysis. HML and HJL did data analysis. HML, QH, XL, and MC wrote and revised the manuscript.

## Funding

This work was supported by NIH/NIMHD Accelerating Excellence in Translational Science Pilot Grants G0814C01 (Q. Hao), and also supported by the ‘Jiangsu Province Excellent Postdoctoral Program’.

## Conflict of interest

The authors declare that the research was conducted in the absence of any commercial or financial relationships that could be construed as a potential conflict of interest.

## Publisher’s note

All claims expressed in this article are solely those of the authors and do not necessarily represent those of their affiliated organizations, or those of the publisher, the editors and the reviewers. Any product that may be evaluated in this article, or claim that may be made by its manufacturer, is not guaranteed or endorsed by the publisher.

## Supplementary material

Supplementary Table 1 Cancer type and sample information.

Supplementary Table 2 Coefficients of 29 lncRNAs estimated by multivariate Cox regression model.
