# Supplementary material for: Oncogenic signaling pathway-related long non-coding RNAs for predicting prognosis and immunotherapy response in breast cancer
Source: Front Immunol. 2022 Aug 4;13:891175. doi: 10.3389/fimmu.2022.891175 (PMC9386474; doi:10.3389/fimmu.2022.891175)
Supplement: Supplementary Table 1 — Cancer type and sample information. [file DataSheet_1.zip › Script/run_analysis_pipeline.html]

run\_analysis\_pipeline


In [1]:

```
options(warns = -1)
suppressMessages(library(dplyr))
suppressMessages(library(Seurat))
suppressMessages(library(GSVA))
suppressMessages(library(ggplot2))
suppressMessages(library(metafor))
suppressMessages(library(readxl))
suppressMessages(library(ConsensusClusterPlus))
suppressMessages(library(glmnet))
suppressMessages(library(limma))
suppressMessages(library(survivalROC))
suppressMessages(library(forestplot))
suppressMessages(library(ggpubr))
suppressMessages(library(topGO))
suppressMessages(library(enrichplot))
suppressMessages(library(clusterProfiler))
suppressMessages(library(e1071))
suppressMessages(library(gridExtra))
suppressMessages(library(grid))
suppressMessages(library(org.Hs.eg.db))
```

In [2]:

```
source('modules/OS.R')
source('modules/global_vars.R')
```

In [3]:

```
out.data.dir <- file.path('../2.results', '1.immune.genes/data')
out.figs.dir <- file.path('../2.results', '1.immune.genes/figs')

dir.create(file.path('../2.results', '1.immune.genes'), showWarnings = FALSE)
dir.create(out.data.dir, showWarnings = FALSE)
dir.create(out.figs.dir, showWarnings = FALSE)
```

### Import 782 marker genes¶

In [4]:

```
markers <- read_excel('../0.data/marker_genes.xlsx', skip = 2) %>% as.data.frame
pathways <- read_excel('../0.data/Tumor_Pathway_Geneset.xlsx') %>% apply(., 2, function(x) x[!is.na(x)] )
```

In [5]:

```
head(markers)
message(sprintf('Number of genes: %g', dim(markers)[1]))
```

A data.frame: 6 × 3

|  | Metagene | Cell type | Immunity |
| --- | --- | --- | --- |
|  | <chr> | <chr> | <chr> |
| 1 | ADAM28 | Activated B cell | Adaptive |
| 2 | CD180 | Activated B cell | Adaptive |
| 3 | CD79B | Activated B cell | Adaptive |
| 4 | BLK | Activated B cell | Adaptive |
| 5 | CD19 | Activated B cell | Adaptive |
| 6 | MS4A1 | Activated B cell | Adaptive |

```
Number of genes: 782
```

### Immune inflitration using ssGSEA method¶

In [6]:

```
marker.lst <- split(markers$Metagene, markers$`Cell type`)
```

In [7]:

```
obj.file <- list.files(path = '../0.data', pattern = 'Obj.rds', full.names = T)
obj.lst <- lapply(obj.file, function(x) { obj <- readRDS(x) })
names(obj.lst) <- gsub('.*/|Obj.rds', '', obj.file)
```

In [8]:

```
tcga.obj <- obj.lst[['tcga']]
nes.score <- gsva(GetAssayData(tcga.obj) %>% as.matrix %>% { log2 (.+1) }, pathways, method = 'ssgsea', kcdf = 'Gaussian') %>% t
```

```
Warning message in .filterFeatures(expr, method):
"1300 genes with constant expression values throuhgout the samples."
```

```
Estimating ssGSEA scores for 10 gene sets.
  |======================================================================| 100%
```

In [9]:

```
nes.score %>% head()
```

A matrix: 6 × 10 of type dbl

|  | RTK\_RAS | NOTCH | HIPPO | WNT | PI3K | CELL\_CYCLE | TGF\_Beta | MYC | TP53 | NRF2 |
| --- | --- | --- | --- | --- | --- | --- | --- | --- | --- | --- |
| TCGA-E2-A15G-01A-11R-A12D-07 | 1.336675 | 1.240839 | 1.192835 | 1.186799 | 1.521849 | 1.356145 | 1.478386 | 1.417893 | 1.419021 | 1.737924 |
| TCGA-E2-A1B5-01A-21R-A12P-07 | 1.313803 | 1.267868 | 1.189044 | 1.260562 | 1.453758 | 1.475970 | 1.470507 | 1.449694 | 1.460431 | 1.694040 |
| TCGA-EW-A2FS-01A-11R-A17B-07 | 1.352823 | 1.248907 | 1.171422 | 1.205154 | 1.420731 | 1.500784 | 1.443278 | 1.347984 | 1.438744 | 1.662352 |
| TCGA-EW-A1P7-01A-21R-A144-07 | 1.396801 | 1.311661 | 1.327573 | 1.349573 | 1.428941 | 1.588284 | 1.447075 | 1.425700 | 1.467098 | 1.670157 |
| TCGA-LL-A5YO-01A-21R-A28M-07 | 1.260467 | 1.279847 | 1.182847 | 1.218694 | 1.414804 | 1.546093 | 1.354670 | 1.407807 | 1.400502 | 1.674053 |
| TCGA-BH-A1FN-01A-11R-A13Q-07 | 1.343863 | 1.314693 | 1.287907 | 1.161825 | 1.474510 | 1.539289 | 1.512237 | 1.495506 | 1.476215 | 1.692673 |

In [10]:

```
sub.clinical <- tcga.obj@meta.data[, c('patient', 'vital_status', 'days_to_death', 'days_to_last_follow_up')]
sub.clinical$Sur <- sub.clinical$vital_status == "Dead"
sub.clinical$Time = ifelse(sub.clinical$Sur, sub.clinical$days_to_death, sub.clinical$days_to_last_follow_up)
```

In [11]:

```
cox.pvals <- sapply(colnames(nes.score), function(pth) {
    sub.clinical$Groups <- nes.score[, pth]
    res.cox <- OS.analysis(sub.clinical, c('#8A0E1A', '#3574CE'), T, cutoff = T)
    fmt <- as.formula(paste('Surv(Time, Sur)~', 'Groups'))
    sub.clinical$Groups <- sub.clinical$Groups > res.cox$cut.off
    coefs <- summary(coxph(fmt, data = sub.clinical))$coefficients[c(2, 5)] 
}) %>% as.data.frame
rownames(cox.pvals) <- c('HR', 'Pval')
cox.pvals
```

A data.frame: 2 × 10

|  | RTK\_RAS | NOTCH | HIPPO | WNT | PI3K | CELL\_CYCLE | TGF\_Beta | MYC | TP53 | NRF2 |
| --- | --- | --- | --- | --- | --- | --- | --- | --- | --- | --- |
|  | <dbl> | <dbl> | <dbl> | <dbl> | <dbl> | <dbl> | <dbl> | <dbl> | <dbl> | <dbl> |
| HR | 1.58157436 | 2.098550e+00 | 1.733758199 | 1.7687960315 | 1.36027482 | 0.448851237 | 2.077437e+00 | 1.790873e+00 | 1.40582128 | 1.2779880 |
| Pval | 0.01225081 | 1.867376e-05 | 0.001630176 | 0.0007110813 | 0.05392389 | 0.005554534 | 3.474881e-05 | 3.972189e-05 | 0.02190491 | 0.1478204 |

### Network visualization¶

In [12]:

```
cols <- ifelse(cox.pvals[1, ] > 1, 'chocolate3', 'darkgreen')
mat <- cor(nes.score)
pdf(file.path(out.figs.dir, 'Cor_networks.pdf'), width = 7, height = 7)
Graph_pcor <- qgraph::qgraph(
    mat,
    layout = "circle",
    labels = colnames(mat),
    borders = T,
    vsize = -log10(cox.pvals[2, ]) * 2,
    graph = 'cor',
    color = cols,
    alpha = 0.0001,
    border.color = cols,
    border.width = 2,
    posCol= 'darkred', 
    negCol = 'darkblue',
    esize = 5 * exp(-15 / 90) + 1,
    label.cex = 1.5,
    label.scale = F
)
legend('bottom',legend=c('0.1', '0.01', '0.001', '0.0001'),pt.cex = -log10(c(0.1, 0.01, 0.001, 0.0001)) * 2, col='black',pch = 21, pt.bg='chocolate3', title = 'Log-rank test, P-value', ncol = 4)
legend('top',legend=c('Positive correlation with P < 0.0001', 'Negative correlation with P < 0.0001'), col=c("darkred", "darkblue"), lty=1:2, lwd = 2)
dev.off()
```

```
Warning message in xtfrm.data.frame(x):
"cannot xtfrm data frames"
```

**png:** 2

In [13]:

```
mat
```

A matrix: 10 × 10 of type dbl

|  | RTK\_RAS | NOTCH | HIPPO | WNT | PI3K | CELL\_CYCLE | TGF\_Beta | MYC | TP53 | NRF2 |
| --- | --- | --- | --- | --- | --- | --- | --- | --- | --- | --- |
| RTK\_RAS | 1.0000000 | 0.5416134 | 0.6719890 | 0.5714008 | 0.59829965 | -0.10518729 | 0.6691979 | 0.25282043 | 0.26695579 | 0.4409344 |
| NOTCH | 0.5416134 | 1.0000000 | 0.6126745 | 0.6302701 | 0.28451209 | 0.12566922 | 0.3940292 | 0.31200395 | -0.01487980 | 0.2015549 |
| HIPPO | 0.6719890 | 0.6126745 | 1.0000000 | 0.7396464 | 0.41436318 | 0.12240206 | 0.6221913 | 0.33455495 | 0.33640794 | 0.2598452 |
| WNT | 0.5714008 | 0.6302701 | 0.7396464 | 1.0000000 | 0.16492358 | 0.18203138 | 0.4263248 | 0.35267868 | 0.10585988 | 0.1540826 |
| PI3K | 0.5982996 | 0.2845121 | 0.4143632 | 0.1649236 | 1.00000000 | -0.27664732 | 0.5656996 | 0.03515346 | 0.19609353 | 0.5058408 |
| CELL\_CYCLE | -0.1051873 | 0.1256692 | 0.1224021 | 0.1820314 | -0.27664732 | 1.00000000 | -0.1447554 | 0.21655767 | 0.06330194 | -0.1018356 |
| TGF\_Beta | 0.6691979 | 0.3940292 | 0.6221913 | 0.4263248 | 0.56569959 | -0.14475540 | 1.0000000 | 0.22540907 | 0.39023036 | 0.3527683 |
| MYC | 0.2528204 | 0.3120040 | 0.3345550 | 0.3526787 | 0.03515346 | 0.21655767 | 0.2254091 | 1.00000000 | 0.17347418 | 0.1213137 |
| TP53 | 0.2669558 | -0.0148798 | 0.3364079 | 0.1058599 | 0.19609353 | 0.06330194 | 0.3902304 | 0.17347418 | 1.00000000 | 0.1477754 |
| NRF2 | 0.4409344 | 0.2015549 | 0.2598452 | 0.1540826 | 0.50584080 | -0.10183557 | 0.3527683 | 0.12131365 | 0.14777535 | 1.0000000 |

### Pathway activity of PAM50 in BC patients¶

In [14]:

```
nes.score.df <- cbind.data.frame(nes.score, PAM50 = tcga.obj@meta.data$paper_BRCA_Subtype_PAM50)
```

In [15]:

```
plot.df <- tidyr::gather(nes.score.df, 'Pathway', 'ssGSEA', -PAM50) %>% .[!is.na(.$PAM50), ]
plot.df$PAM50 <- factor(plot.df$PAM50, levels = names(PAM50_COLOR))
plot.df$Pathway <- factor(plot.df$Pathway, levels = names(pathways))
```

In [16]:

```
head(plot.df)
```

A data.frame: 6 × 3

|  | PAM50 | Pathway | ssGSEA |
| --- | --- | --- | --- |
|  | <fct> | <fct> | <dbl> |
| 1 | LumA | RTK\_RAS | 1.336675 |
| 2 | LumA | RTK\_RAS | 1.313803 |
| 3 | LumA | RTK\_RAS | 1.352823 |
| 4 | Normal | RTK\_RAS | 1.396801 |
| 5 | Her2 | RTK\_RAS | 1.260467 |
| 6 | LumB | RTK\_RAS | 1.343863 |

In [17]:

```
options(repr.plot.width = 20, repr.plot.height = 8)
ggplot(data = plot.df, aes(x = Pathway, y = ssGSEA, fill = PAM50)) + geom_boxplot() + theme_classic(base_size = 20) + scale_fill_manual(values = unlist(PAM50_COLOR))
```

In [18]:

```
ggsave(file.path(out.figs.dir, 'pam50_pathways_boxplot.pdf'), width = 16, height = 6)
```

### Association with long non-coding RNA¶

In [19]:

```
nes.score.sub <- nes.score[, cox.pvals[2, ] < 0.05]
dim(nes.score.sub)
```

1. 1222
2. 8

In [20]:

```
lincrna <- read.table('../0.data/gencode.gene.info.v22.tsv', sep = '\t', header = T) %>% subset(., gene_type == 'lincRNA') 
dim(lincrna)
head(lincrna)
```

1. 7656
2. 12

A data.frame: 6 × 12

|  | gene\_id | gene\_name | seqname | start | end | strand | gene\_type | gene\_status | havana\_gene | full\_length | exon\_length | exon\_num |
| --- | --- | --- | --- | --- | --- | --- | --- | --- | --- | --- | --- | --- |
|  | <chr> | <chr> | <chr> | <int> | <int> | <chr> | <chr> | <chr> | <chr> | <int> | <int> | <int> |
| 2 | ENSG00000238009.5 | RP11-34P13.7 | chr1 | 89295 | 133723 | - | lincRNA | NOVEL | OTTHUMG00000001096.2 | 44429 | 3726 | 17 |
| 3 | ENSG00000230415.1 | RP5-902P8.10 | chr1 | 1275223 | 1280420 | + | lincRNA | NOVEL | OTTHUMG00000002234.2 | 5198 | 513 | 5 |
| 4 | ENSG00000236335.1 | RP4-591L5.1 | chr1 | 30409560 | 30411638 | - | lincRNA | NOVEL | OTTHUMG00000003682.1 | 2079 | 507 | 3 |
| 15 | ENSG00000231949.1 | RP4-591L5.2 | chr1 | 30415825 | 30421108 | + | lincRNA | NOVEL | OTTHUMG00000037511.1 | 5284 | 481 | 3 |
| 28 | ENSG00000271324.1 | RP11-10C24.2 | chr3 | 33793644 | 33794145 | - | lincRNA | NOVEL | OTTHUMG00000184931.1 | 502 | 502 | 1 |
| 29 | ENSG00000271643.1 | RP11-10C24.3 | chr3 | 33795688 | 33796950 | - | lincRNA | NOVEL | OTTHUMG00000185053.1 | 1263 | 1263 | 1 |

In [21]:

```
lincrna.ovp <- intersect(lincrna$gene_name, rownames(tcga.obj))
lincrna.ovp <- lincrna.ovp[-grep('^RP', lincrna.ovp)]
lincrna.ovp <- lincrna.ovp[-grep('^MT', lincrna.ovp)]
length(lincrna.ovp)
```

3006

In [22]:

```
options(warn = -1)
expr <- FetchData(tcga.obj, vars = lincrna.ovp) %>% as.matrix %>% { log2(1 + .) }
path2lincrnas <- lapply(colnames(nes.score.sub), function(pth) {
    score.tmp <- nes.score.sub[, pth]   
    cors <- sapply(lincrna.ovp, function(gene) { cor(expr[, gene], score.tmp)})   
    cors <- cors[!is.na(cors)]
    cors[cors > quantile(cors, 0.95)]
})
```

In [23]:

```
names(path2lincrnas) <- colnames(nes.score.sub)
saveRDS(path2lincrnas, file = file.path(out.data.dir, 'path2lincrnas.rds'))
```

In [24]:

```
pdf(file.path(out.figs.dir, 'Corr_pathways2LincRNA.pdf'), width = 12, height = 6)
boxplot(path2lincrnas, col = gg_color_hue(length(path2lincrnas)), lwd = 1.2, ylab = 'Correlation (r)') 
dev.off()
```

**png:** 2

In [25]:

```
ovp.mat <- lapply(path2lincrnas, function(x) {
    ovp <- lapply(path2lincrnas, function(y) {
        length(intersect(names(x), names(y)))
    }) %>% unlist(.)
}) %>% as.data.frame
```

In [26]:

```
ovp.mat
```

A data.frame: 8 × 8

|  | RTK\_RAS | NOTCH | HIPPO | WNT | CELL\_CYCLE | TGF\_Beta | MYC | TP53 |
| --- | --- | --- | --- | --- | --- | --- | --- | --- |
|  | <int> | <int> | <int> | <int> | <int> | <int> | <int> | <int> |
| RTK\_RAS | 149 | 92 | 102 | 90 | 1 | 117 | 44 | 39 |
| NOTCH | 92 | 149 | 107 | 122 | 12 | 83 | 63 | 15 |
| HIPPO | 102 | 107 | 149 | 114 | 15 | 93 | 72 | 36 |
| WNT | 90 | 122 | 114 | 149 | 18 | 78 | 74 | 18 |
| CELL\_CYCLE | 1 | 12 | 15 | 18 | 149 | 0 | 55 | 9 |
| TGF\_Beta | 117 | 83 | 93 | 78 | 0 | 149 | 35 | 52 |
| MYC | 44 | 63 | 72 | 74 | 55 | 35 | 149 | 18 |
| TP53 | 39 | 15 | 36 | 18 | 9 | 52 | 18 | 149 |

In [27]:

```
text.labels <- ovp.mat
pheatmap::pheatmap(
    ovp.mat,
    display_numbers = text.labels,
    fontsize_number = 12,
    width = 8,
    height = 6,
    filename = sprintf('%s/Heatmap.selected.lincgenes.pdf', out.figs.dir)
)
```

In [28]:

```
uniq.linc.genes <- lapply(path2lincrnas, function(x) names(x)) %>% unlist(.) %>% unique
```

In [29]:

```
length(uniq.linc.genes)
```

498

### Select prognosis-related lincRNAs using Lasso-cox regression method¶

In [30]:

```
label <- ifelse(tcga.obj@meta.data$sample_type %in% c('Primary Tumor', 'Metastatic'), 'Tumor', 'Normal')
uniq.linc.genes.sub <- uniq.linc.genes
```

In [31]:

```
tcga.obj.sub <- subset(tcga.obj, sample_type == 'Primary Tumor' | sample_type == 'Metastatic')
exprs <- FetchData(tcga.obj.sub, vars = uniq.linc.genes.sub) %>% as.matrix %>% {log2(1 + .)}
colnames(exprs) <- gsub('-', '_', colnames(exprs))
sub.clinical <- tcga.obj.sub@misc[['Clin']][colnames(tcga.obj.sub), ]

select.sets.sub <- sapply(colnames(exprs), function(x) {
    sub.clinical$Groups <- exprs[, x]
    tryCatch({
        res.cox <- OS.analysis(sub.clinical, NULL, T, cutoff = T, prefix = NULL)
    }, warning = function(w) { print('Warning...')}, error = function(e) { return(NA)})
}) %>% do.call(cbind, .)
gsets.sig <- select.sets.sub[, select.sets.sub[1, ] < 0.01] %>% colnames
gsets.sig <- gsets.sig[!is.na(gsets.sig)]
idxes <- which(sub.clinical[, 'Time'] > 0)
fit <- glmnet(exprs[idxes, gsets.sig], Surv(sub.clinical[idxes, 'Time'], sub.clinical[idxes, 'Sur']), family = 'cox', type.measure = 'deviance')
fitcv <- cv.glmnet(exprs[idxes, gsets.sig], Surv(sub.clinical[idxes, 'Time'], sub.clinical[idxes, 'Sur']), family = 'cox', alpha=1, nfolds=10, type.measure = 'deviance')
```

In [32]:

```
pdf(file.path(out.figs.dir, 'Lasso_fitCV_threshold_using_celltypes.pdf'), width = 6, height = 6)
plot(fitcv)
dev.off()

pdf(file.path(out.figs.dir, 'Lasso_coefficients_threshold_using_celltypes.pdf'), width = 8, height = 6)
plot(fit, xvar = "lambda",label = T, lwd = 2, cex = 3, col = gg_color_hue(length(path2lincrnas)))
abline(v = log(fitcv$lambda.min), col = "purple", lty = 2, lwd = 2)
dev.off()
```

**png:** 2

**png:** 2

In [33]:

```
coefs <- coef(fitcv, s = fitcv$lambda.min)
hub.genes <- rownames(coefs)[which(as.numeric(coefs) != 0)]
hub.genes
hub.genes %>% length
```

1. 'CTB\_33O18.1'
2. 'AF131215.8'
3. 'LINC01235'
4. 'LINC00987'
5. 'LINC00398'
6. 'SLC25A30\_AS1'
7. 'FGF14\_AS2'
8. 'LINC00667'
9. 'CTD\_2554C21.2'
10. 'ZNF888'
11. 'LL0XNC01\_237H1.2'
12. 'AC016995.3'
13. 'SFTA1P'
14. 'AL022344.7'
15. 'LINC00958'
16. 'LINC00327'
17. 'LINC00346'
18. 'LINC00519'
19. 'AC016735.1'
20. 'AC009495.2'
21. 'LINC00900'
22. 'AC092431.3'
23. 'CTC\_498J12.1'
24. 'LINC01508'
25. 'LINC00707'
26. 'GATA6\_AS1'
27. 'AC092580.4'
28. 'CTA\_384D8.35'
29. 'CTA\_384D8.34'
30. 'MIR4435\_1HG'
31. 'CTC\_490E21.10'
32. 'MLLT4\_AS1'
33. 'EDNRB\_AS1'
34. 'CTC\_459F4.1'
35. 'MIAT'

35

In [34]:

```
formula.fmt <- as.formula(paste0('Surv(Time, Sur)~', paste(hub.genes, sep = ' ', collapse = '+')))
dat <- cbind(sub.clinical[, c('Time', 'Sur')], exprs[, hub.genes])
mult.var.cox <- coxph(formula.fmt, data = dat)
ph.res <- cox.zph(mult.var.cox) 
ph.res <- ph.res$table[-nrow(ph.res$table), ]
hub.genes.sub <- rownames(ph.res)[ph.res[, 3] > 0.05]
```

In [35]:

```
#hub.genes.sub <- setdiff(hub.genes.sub, c('LINC01508', 'AL022344.7', 'AF131215.8', 'GATA6_AS1'))
hub.genes.sub
```

1. 'AF131215.8'
2. 'LINC01235'
3. 'LINC00987'
4. 'LINC00398'
5. 'LINC00667'
6. 'CTD\_2554C21.2'
7. 'ZNF888'
8. 'LL0XNC01\_237H1.2'
9. 'AC016995.3'
10. 'SFTA1P'
11. 'AL022344.7'
12. 'LINC00958'
13. 'LINC00327'
14. 'LINC00346'
15. 'AC016735.1'
16. 'AC009495.2'
17. 'AC092431.3'
18. 'CTC\_498J12.1'
19. 'LINC01508'
20. 'LINC00707'
21. 'GATA6\_AS1'
22. 'AC092580.4'
23. 'CTA\_384D8.35'
24. 'CTA\_384D8.34'
25. 'MIR4435\_1HG'
26. 'MLLT4\_AS1'
27. 'EDNRB\_AS1'
28. 'CTC\_459F4.1'
29. 'MIAT'

In [36]:

```
formula.fmt <- as.formula(paste0('Surv(Time, Sur)~', paste(hub.genes.sub, sep = ' ', collapse = '+')))
dat <- cbind(sub.clinical[, c('Time', 'Sur')], exprs[, hub.genes.sub])
```

In [37]:

```
expr.hubs <- FetchData(tcga.obj.sub, vars = gsub('_', '-', hub.genes.sub))
```

In [38]:

```
cor(expr.hubs)
```

A matrix: 29 × 29 of type dbl

|  | AF131215.8 | LINC01235 | LINC00987 | LINC00398 | LINC00667 | CTD-2554C21.2 | ZNF888 | LL0XNC01-237H1.2 | AC016995.3 | SFTA1P | ... | LINC00707 | GATA6-AS1 | AC092580.4 | CTA-384D8.35 | CTA-384D8.34 | MIR4435-1HG | MLLT4-AS1 | EDNRB-AS1 | CTC-459F4.1 | MIAT |
| --- | --- | --- | --- | --- | --- | --- | --- | --- | --- | --- | --- | --- | --- | --- | --- | --- | --- | --- | --- | --- | --- |
| AF131215.8 | 1.000000000 | 0.030659689 | 0.1371626736 | -0.013242921 | 0.150809294 | 0.049047174 | -0.09765492 | -0.02741655 | 0.082528913 | 0.094769667 | ... | -0.002307675 | 0.0886171901 | 0.018273618 | -0.0293107732 | -0.019550151 | 0.001052183 | 0.134996091 | 0.0213944970 | 0.063829757 | -0.012640405 |
| LINC01235 | 0.030659689 | 1.000000000 | 0.0915942269 | -0.011515421 | -0.003045315 | 0.046283384 | -0.07729871 | -0.04147945 | 0.102774596 | 0.041808008 | ... | 0.088567057 | 0.0601783548 | 0.059067864 | 0.0868840173 | -0.002025324 | 0.080979979 | 0.083613576 | 0.0049687543 | 0.001034400 | -0.018838296 |
| LINC00987 | 0.137162674 | 0.091594227 | 1.0000000000 | -0.074309506 | 0.097940884 | 0.044328857 | -0.09412685 | -0.04432184 | 0.218618564 | 0.015850165 | ... | -0.010416209 | 0.1348154625 | 0.099391726 | 0.0073327720 | -0.017341446 | 0.011541206 | 0.155667644 | 0.0110084579 | -0.002356759 | 0.005072007 |
| LINC00398 | -0.013242921 | -0.011515421 | -0.0743095059 | 1.000000000 | 0.093656760 | -0.021327132 | 0.08198764 | 0.11951749 | -0.123998204 | -0.080589020 | ... | -0.081163895 | -0.1158156059 | -0.140560758 | -0.1385034167 | -0.067258529 | -0.114778852 | -0.112701076 | 0.0099759710 | 0.034727277 | 0.020864362 |
| LINC00667 | 0.150809294 | -0.003045315 | 0.0979408837 | 0.093656760 | 1.000000000 | 0.127383635 | -0.01361608 | 0.17713089 | 0.157541011 | 0.005790952 | ... | -0.057500213 | 0.0658108205 | 0.017676528 | -0.0533958971 | -0.059758620 | -0.067186224 | 0.136555664 | 0.1351481883 | 0.130344239 | 0.102566801 |
| CTD-2554C21.2 | 0.049047174 | 0.046283384 | 0.0443288574 | -0.021327132 | 0.127383635 | 1.000000000 | 0.14494188 | 0.18280806 | 0.126616334 | -0.049427372 | ... | 0.119826844 | 0.1253770488 | -0.031323489 | 0.0337614867 | -0.011324523 | -0.047498428 | 0.181148785 | 0.0344428689 | 0.178291763 | 0.008090492 |
| ZNF888 | -0.097654919 | -0.077298709 | -0.0941268508 | 0.081987641 | -0.013616077 | 0.144941877 | 1.00000000 | 0.39804720 | -0.104612492 | -0.139859262 | ... | -0.048219544 | -0.1129583483 | -0.105521492 | -0.1430256867 | -0.083084588 | -0.092195898 | -0.077418952 | 0.1822520463 | 0.210590088 | 0.107861181 |
| LL0XNC01-237H1.2 | -0.027416548 | -0.041479452 | -0.0443218400 | 0.119517487 | 0.177130890 | 0.182808061 | 0.39804720 | 1.00000000 | -0.059450588 | -0.074041946 | ... | -0.034125031 | -0.0832644639 | -0.105851416 | -0.1259714342 | -0.080950549 | 0.090990065 | 0.016552386 | 0.5217998245 | 0.555406960 | 0.277700028 |
| AC016995.3 | 0.082528913 | 0.102774596 | 0.2186185636 | -0.123998204 | 0.157541011 | 0.126616334 | -0.10461249 | -0.05945059 | 1.000000000 | 0.023633526 | ... | 0.061477511 | 0.2842637050 | 0.199675402 | 0.2610306437 | 0.068159419 | 0.017136333 | 0.230806016 | 0.0184211158 | 0.057916960 | 0.003107471 |
| SFTA1P | 0.094769667 | 0.041808008 | 0.0158501648 | -0.080589020 | 0.005790952 | -0.049427372 | -0.13985926 | -0.07404195 | 0.023633526 | 1.000000000 | ... | -0.003862823 | 0.0334130476 | 0.020593950 | 0.0115679033 | 0.005587912 | 0.302263146 | 0.003369025 | 0.0532314446 | 0.062160436 | 0.017239618 |
| AL022344.7 | 0.085190202 | 0.059140926 | 0.1174687286 | -0.094147454 | 0.043046581 | 0.125390015 | -0.10635482 | -0.03568416 | 0.166139869 | 0.017970766 | ... | 0.182953260 | 0.2678607858 | 0.030237735 | 0.1258589215 | 0.005634605 | 0.118546018 | 0.204903832 | 0.0275981040 | 0.055576313 | 0.043998474 |
| LINC00958 | 0.074305193 | 0.019058317 | 0.0436509466 | -0.019911175 | 0.047694920 | 0.043943029 | -0.02182726 | -0.04554834 | 0.042540255 | 0.014004712 | ... | -0.016283456 | 0.0272291887 | -0.002483873 | -0.0041880640 | -0.020207610 | -0.038729388 | -0.003229988 | -0.0182843660 | -0.004664645 | -0.042297001 |
| LINC00327 | 0.080142312 | -0.003821150 | 0.1239426641 | 0.025434389 | 0.060255428 | 0.076179169 | -0.04918807 | 0.10601622 | 0.052533572 | 0.034049263 | ... | -0.016304307 | 0.0521793972 | 0.027644464 | -0.0558948801 | -0.040393594 | 0.081600230 | -0.053567031 | 0.1144779335 | 0.127731068 | 0.062954229 |
| LINC00346 | 0.017100965 | 0.066416370 | -0.0212464900 | -0.030859135 | -0.052472110 | 0.128915988 | -0.02363447 | -0.02673769 | 0.057211186 | 0.046279659 | ... | 0.212183725 | 0.0812100650 | -0.044299860 | 0.1277173027 | 0.011036729 | 0.080011532 | 0.144182202 | -0.0039672567 | 0.020441144 | -0.022812771 |
| AC016735.1 | 0.127956374 | 0.119526424 | 0.1252801664 | -0.060805651 | 0.082424379 | 0.181613670 | 0.01391167 | 0.13849789 | 0.098503526 | 0.036389537 | ... | 0.102291454 | 0.0834383161 | 0.060525243 | 0.1038982994 | 0.026683593 | 0.210485697 | 0.199468414 | 0.0585451014 | 0.355317128 | 0.005144716 |
| AC009495.2 | 0.124675687 | 0.132015050 | 0.1880802184 | -0.080076652 | 0.134810422 | 0.185259174 | -0.01637652 | 0.05597678 | 0.284301892 | -0.004375553 | ... | 0.083646478 | 0.1598242343 | 0.199660746 | 0.2359964018 | 0.099014002 | 0.101234721 | 0.245520285 | 0.1266565749 | 0.192847136 | 0.111923443 |
| AC092431.3 | 0.005557637 | 0.058429413 | 0.0006159652 | 0.109173352 | 0.112044256 | 0.112851325 | 0.07851498 | 0.38180396 | -0.010067426 | 0.009927376 | ... | -0.017172828 | 0.0002562205 | -0.053773507 | -0.0462190722 | -0.042173054 | 0.093198965 | 0.126376720 | 0.2034315139 | 0.308386853 | 0.150114225 |
| CTC-498J12.1 | 0.094514567 | 0.124407850 | 0.1009323676 | -0.058878769 | -0.001234742 | -0.020110936 | -0.01379975 | 0.05373264 | 0.092981997 | 0.017527433 | ... | -0.011071164 | 0.0467660418 | 0.111512379 | 0.0841498997 | 0.029906813 | 0.197806391 | 0.088904006 | 0.1633934293 | 0.086771019 | 0.098017235 |
| LINC01508 | -0.011758458 | 0.063040269 | 0.0122435072 | -0.173830945 | -0.025202972 | 0.151803948 | -0.07878093 | -0.07093124 | 0.214428478 | -0.017700594 | ... | 0.138184345 | 0.1763740182 | 0.165514661 | 0.2676979337 | 0.096455941 | 0.027056475 | 0.170316131 | 0.0305560754 | 0.046056353 | -0.004832725 |
| LINC00707 | -0.002307675 | 0.088567057 | -0.0104162093 | -0.081163895 | -0.057500213 | 0.119826844 | -0.04821954 | -0.03412503 | 0.061477511 | -0.003862823 | ... | 1.000000000 | 0.0819109618 | 0.015987013 | 0.2105954192 | 0.026243559 | 0.031444184 | 0.124893432 | 0.0083597068 | 0.026987831 | -0.030396232 |
| GATA6-AS1 | 0.088617190 | 0.060178355 | 0.1348154625 | -0.115815606 | 0.065810820 | 0.125377049 | -0.11295835 | -0.08326446 | 0.284263705 | 0.033413048 | ... | 0.081910962 | 1.0000000000 | 0.088361081 | 0.2247654463 | 0.052054363 | 0.105791248 | 0.343622876 | 0.0232220033 | 0.039528916 | 0.028929987 |
| AC092580.4 | 0.018273618 | 0.059067864 | 0.0993917259 | -0.140560758 | 0.017676528 | -0.031323489 | -0.10552149 | -0.10585142 | 0.199675402 | 0.020593950 | ... | 0.015987013 | 0.0883610810 | 1.000000000 | 0.2564053845 | 0.248913746 | 0.077882555 | 0.042348701 | 0.0547892306 | -0.022849714 | 0.294183628 |
| CTA-384D8.35 | -0.029310773 | 0.086884017 | 0.0073327720 | -0.138503417 | -0.053395897 | 0.033761487 | -0.14302569 | -0.12597143 | 0.261030644 | 0.011567903 | ... | 0.210595419 | 0.2247654463 | 0.256405384 | 1.0000000000 | 0.628244445 | 0.119107200 | 0.100999241 | -0.0009140464 | -0.001995572 | 0.058626923 |
| CTA-384D8.34 | -0.019550151 | -0.002025324 | -0.0173414457 | -0.067258529 | -0.059758620 | -0.011324523 | -0.08308459 | -0.08095055 | 0.068159419 | 0.005587912 | ... | 0.026243559 | 0.0520543629 | 0.248913746 | 0.6282444452 | 1.000000000 | 0.033518560 | -0.008760779 | -0.0112179997 | -0.015378260 | 0.090480963 |
| MIR4435-1HG | 0.001052183 | 0.080979979 | 0.0115412057 | -0.114778852 | -0.067186224 | -0.047498428 | -0.09219590 | 0.09099007 | 0.017136333 | 0.302263146 | ... | 0.031444184 | 0.1057912482 | 0.077882555 | 0.1191072004 | 0.033518560 | 1.000000000 | 0.106570339 | 0.2404146762 | 0.309562373 | 0.180501250 |
| MLLT4-AS1 | 0.134996091 | 0.083613576 | 0.1556676436 | -0.112701076 | 0.136555664 | 0.181148785 | -0.07741895 | 0.01655239 | 0.230806016 | 0.003369025 | ... | 0.124893432 | 0.3436228760 | 0.042348701 | 0.1009992405 | -0.008760779 | 0.106570339 | 1.000000000 | 0.0576991745 | 0.117962336 | 0.002198176 |
| EDNRB-AS1 | 0.021394497 | 0.004968754 | 0.0110084579 | 0.009975971 | 0.135148188 | 0.034442869 | 0.18225205 | 0.52179982 | 0.018421116 | 0.053231445 | ... | 0.008359707 | 0.0232220033 | 0.054789231 | -0.0009140464 | -0.011218000 | 0.240414676 | 0.057699174 | 1.0000000000 | 0.584794831 | 0.263713437 |
| CTC-459F4.1 | 0.063829757 | 0.001034400 | -0.0023567588 | 0.034727277 | 0.130344239 | 0.178291763 | 0.21059009 | 0.55540696 | 0.057916960 | 0.062160436 | ... | 0.026987831 | 0.0395289163 | -0.022849714 | -0.0019955724 | -0.015378260 | 0.309562373 | 0.117962336 | 0.5847948308 | 1.000000000 | 0.163463331 |
| MIAT | -0.012640405 | -0.018838296 | 0.0050720069 | 0.020864362 | 0.102566801 | 0.008090492 | 0.10786118 | 0.27770003 | 0.003107471 | 0.017239618 | ... | -0.030396232 | 0.0289299875 | 0.294183628 | 0.0586269231 | 0.090480963 | 0.180501250 | 0.002198176 | 0.2637134374 | 0.163463331 | 1.000000000 |

In [39]:

```
require(corrplot)
M <- cor(expr.hubs)
p.mat <- cor.mtest(expr.hubs)$p
col <- colorRampPalette((c("dodgerblue4", "dodgerblue", "darkorange", "firebrick1")))

outfile <- sprintf('%s/lincrna_expr_corr.pdf', out.figs.dir)
pdf(outfile, width = 8, height = 8)
corrplot(M, 
         col = col(200),
         order = "hclust", 
         tl.col = "black", insig = 'blank',
         p.mat = p.mat, sig.level = 0.01, addrect = 2, cl.cex = 1.5)
dev.off()
```

```
载入需要的程辑包：corrplot

corrplot 0.90 loaded
```

**png:** 2

In [40]:

```
set.seed(123456)
train.SN <- sample(rownames(dat), as.integer(dim(dat)[1] / 2), replace = FALSE)
test.SN <- setdiff(rownames(dat), train.SN)
```

In [41]:

```
mult.var.cox <- coxph(formula.fmt, data = dat[train.SN, ])
mult.var.cox
saveRDS(mult.var.cox, file = file.path(out.data.dir, 'mult.var.cox.rds'))
```

```
Call:
coxph(formula = formula.fmt, data = dat[train.SN, ])

                      coef exp(coef)  se(coef)      z       p
AF131215.8        1.306598  3.693586  0.493032  2.650 0.00805
LINC01235         0.303676  1.354830  0.166620  1.823 0.06837
LINC00987        -1.247201  0.287308  0.465950 -2.677 0.00744
LINC00398         0.443267  1.557788  0.505438  0.877 0.38049
LINC00667        -0.216508  0.805326  0.264472 -0.819 0.41299
CTD_2554C21.2    -0.345267  0.708032  0.216716 -1.593 0.11112
ZNF888            0.468164  1.597060  0.295095  1.586 0.11263
LL0XNC01_237H1.2  0.684621  1.983021  0.412142  1.661 0.09669
AC016995.3       -0.313227  0.731084  0.206437 -1.517 0.12919
SFTA1P            0.474085  1.606543  0.365544  1.297 0.19466
AL022344.7        0.872667  2.393285  0.592120  1.474 0.14054
LINC00958         0.308412  1.361261  0.218652  1.411 0.15839
LINC00327        -0.702556  0.495318  1.390018 -0.505 0.61326
LINC00346         0.453466  1.573758  0.166330  2.726 0.00640
AC016735.1        0.035821  1.036470  0.261241  0.137 0.89094
AC009495.2       -0.620275  0.537797  0.519722 -1.193 0.23268
AC092431.3        2.245101  9.441367  0.777346  2.888 0.00388
CTC_498J12.1     -0.886238  0.412203  0.543939 -1.629 0.10325
LINC01508         0.512718  1.669824  0.359671  1.426 0.15401
LINC00707        -0.094513  0.909816  0.197373 -0.479 0.63204
GATA6_AS1         0.069956  1.072461  0.526543  0.133 0.89430
AC092580.4        0.269505  1.309316  0.256080  1.052 0.29260
CTA_384D8.35     -0.082341  0.920958  0.218169 -0.377 0.70586
CTA_384D8.34     -0.456453  0.633527  0.499327 -0.914 0.36064
MIR4435_1HG       0.236335  1.266599  0.350718  0.674 0.50040
MLLT4_AS1         0.865814  2.376940  0.307453  2.816 0.00486
EDNRB_AS1        -5.850243  0.002879  4.271310 -1.370 0.17079
CTC_459F4.1      -1.003262  0.366681  0.486017 -2.064 0.03899
MIAT             -0.216377  0.805431  0.349922 -0.618 0.53634

Likelihood ratio test=72.81  on 29 df, p=1.238e-05
n= 552, number of events= 73 
   (因为不存在，2个观察量被删除了)
```

In [42]:

```
options(repr.plot.width = 10, repr.plot.height = 8)
coefs <- mult.var.cox$coefficients[!is.na(mult.var.cox$coefficients)]
select.sets <- names(coefs)
risk.socres <- coefs %*% t(as.matrix(exprs[train.SN, select.sets]))
sub.clinical.train <- sub.clinical[train.SN, ]
sub.clinical.train$Groups <- as.vector(risk.socres) #> median(risk.socres)
res.cox <- OS.analysis(
    sub.clinical.train, 
    c('#8A0E1A', '#3574CE'), 
    F, 
    cutoff = T, 
    prefix = 'TCGA_selected_genesets_OS_train.pdf',
    out.figs.dir = out.figs.dir,
    title = sprintf('TCGA-BRAC data (n = %g)', dim(sub.clinical.train)[1])
)
```

In [43]:

```
fmt <- as.formula('Surv(Time, Sur)~Groups')
sub.clinical.train$Groups <- sub.clinical.train$Groups > res.cox$cutoff
coxph(fmt, data = sub.clinical.train) %>% summary
```

```
Call:
coxph(formula = fmt, data = sub.clinical.train)

  n= 552, number of events= 73 
   (因为不存在，2个观察量被删除了)

             coef exp(coef) se(coef)     z Pr(>|z|)    
GroupsTRUE 2.0548    7.8056   0.2519 8.157 3.44e-16 ***
---
Signif. codes:  0 '***' 0.001 '**' 0.01 '*' 0.05 '.' 0.1 ' ' 1

           exp(coef) exp(-coef) lower .95 upper .95
GroupsTRUE     7.806     0.1281     4.764     12.79

Concordance= 0.686  (se = 0.034 )
Likelihood ratio test= 55.38  on 1 df,   p=1e-13
Wald test            = 66.53  on 1 df,   p=3e-16
Score (logrank) test = 91.71  on 1 df,   p=<2e-16
```

In [44]:

```
coefs <- mult.var.cox$coefficients[!is.na(mult.var.cox$coefficients)]
select.sets <- names(coefs)
risk.socres <- coefs %*% t(as.matrix(exprs[test.SN, select.sets]))
sub.clinical.test <- sub.clinical[test.SN, ]
sub.clinical.test$Groups <- as.vector(risk.socres) #> median(risk.socres)
res.cox <- OS.analysis(
    sub.clinical.test, 
    c('#8A0E1A', '#3574CE'), 
    F, 
    cutoff = T, 
    prefix = 'TCGA_selected_genesets_OS_test.pdf',
    out.figs.dir = out.figs.dir,
    title = sprintf('TCGA-BRAC data (n = %g)', dim(sub.clinical.test)[1])
)
```

In [45]:

```
fmt <- as.formula('Surv(Time, Sur)~Groups')
sub.clinical.test$Groups <- sub.clinical.test$Groups > res.cox$cutoff
coxph(fmt, data = sub.clinical.test) %>% summary
```

```
Call:
coxph(formula = fmt, data = sub.clinical.test)

  n= 555, number of events= 83 

            coef exp(coef) se(coef)     z Pr(>|z|)    
GroupsTRUE 1.456     4.288    0.230 6.329 2.47e-10 ***
---
Signif. codes:  0 '***' 0.001 '**' 0.01 '*' 0.05 '.' 0.1 ' ' 1

           exp(coef) exp(-coef) lower .95 upper .95
GroupsTRUE     4.288     0.2332     2.732      6.73

Concordance= 0.698  (se = 0.031 )
Likelihood ratio test= 41  on 1 df,   p=2e-10
Wald test            = 40.05  on 1 df,   p=2e-10
Score (logrank) test = 47.53  on 1 df,   p=5e-12
```

### Adjust coefficients using all data¶

In [46]:

```
mult.var.cox <- coxph(formula.fmt, data = dat)
mult.var.cox
saveRDS(mult.var.cox, file = file.path(out.data.dir, 'mult.var.cox.adjusted..rds'))
```

```
Call:
coxph(formula = formula.fmt, data = dat)

                     coef exp(coef) se(coef)      z        p
AF131215.8        0.84749   2.33379  0.24526  3.455 0.000549
LINC01235         0.41106   1.50841  0.08869  4.635 3.58e-06
LINC00987        -0.58175   0.55892  0.26067 -2.232 0.025632
LINC00398        -0.30935   0.73392  0.37175 -0.832 0.405323
LINC00667        -0.31513   0.72969  0.16600 -1.898 0.057641
CTD_2554C21.2    -0.40370   0.66784  0.14182 -2.847 0.004419
ZNF888            0.41437   1.51341  0.19110  2.168 0.030137
LL0XNC01_237H1.2  0.75157   2.12034  0.27459  2.737 0.006199
AC016995.3       -0.20816   0.81208  0.13269 -1.569 0.116696
SFTA1P            0.43960   1.55209  0.21394  2.055 0.039904
AL022344.7        1.06178   2.89153  0.37784  2.810 0.004952
LINC00958         0.16076   1.17440  0.13235  1.215 0.224491
LINC00327        -1.26541   0.28212  0.93046 -1.360 0.173835
LINC00346         0.41871   1.52001  0.11903  3.518 0.000435
AC016735.1       -0.22637   0.79742  0.16471 -1.374 0.169328
AC009495.2       -0.23897   0.78744  0.30079 -0.794 0.426916
AC092431.3        1.41046   4.09786  0.54044  2.610 0.009058
CTC_498J12.1     -0.46568   0.62771  0.29690 -1.568 0.116766
LINC01508         0.67767   1.96928  0.21082  3.214 0.001307
LINC00707        -0.35293   0.70263  0.16721 -2.111 0.034795
GATA6_AS1         0.34364   1.41007  0.24193  1.420 0.155493
AC092580.4       -0.06501   0.93706  0.16295 -0.399 0.689927
CTA_384D8.35     -0.13445   0.87420  0.13613 -0.988 0.323324
CTA_384D8.34     -0.15232   0.85871  0.29252 -0.521 0.602551
MIR4435_1HG       0.37797   1.45932  0.20518  1.842 0.065458
MLLT4_AS1         0.36861   1.44572  0.22448  1.642 0.100586
EDNRB_AS1        -4.24835   0.01429  2.56798 -1.654 0.098055
CTC_459F4.1      -0.72554   0.48407  0.33383 -2.173 0.029754
MIAT             -0.26078   0.77045  0.22050 -1.183 0.236944

Likelihood ratio test=140.5  on 29 df, p=< 2.2e-16
n= 1107, number of events= 156 
   (因为不存在，2个观察量被删除了)
```

In [47]:

```
coefs <- mult.var.cox$coefficients[!is.na(mult.var.cox$coefficients)]
select.sets <- names(coefs)
risk.socres <- coefs %*% t(as.matrix(exprs[, select.sets]))
sub.clinical$Groups <- as.vector(risk.socres) #> median(risk.socres)
res.cox <- OS.analysis(
    sub.clinical, 
    c('#8A0E1A', '#3574CE'), 
    F, 
    cutoff = T, 
    prefix = 'TCGA_selected_genesets_OS_all.pdf',
    out.figs.dir = out.figs.dir,
    title = sprintf('TCGA-BRAC data (n = %g)', dim(sub.clinical)[1])
)
#res.cox$group <- ifelse(res.cox$group == FALSE, 'High risk', 'Low risk')
#res.cox$cut.off <- median(risk.socres)
```

In [48]:

```
fmt <- as.formula('Surv(Time, Sur)~Groups')
sub.clinical$Groups <- sub.clinical$Groups > res.cox$cutoff
coxph(fmt, data = sub.clinical) %>% summary
```

```
Call:
coxph(formula = fmt, data = sub.clinical)

  n= 1107, number of events= 156 
   (因为不存在，2个观察量被删除了)

             coef exp(coef) se(coef)     z Pr(>|z|)    
GroupsTRUE 2.0175    7.5194   0.1686 11.97   <2e-16 ***
---
Signif. codes:  0 '***' 0.001 '**' 0.01 '*' 0.05 '.' 0.1 ' ' 1

           exp(coef) exp(-coef) lower .95 upper .95
GroupsTRUE     7.519      0.133     5.404     10.46

Concordance= 0.694  (se = 0.023 )
Likelihood ratio test= 120  on 1 df,   p=<2e-16
Wald test            = 143.3  on 1 df,   p=<2e-16
Score (logrank) test = 196  on 1 df,   p=<2e-16
```

In [49]:

```
head(sub.clinical)
```

A data.frame: 6 × 7

|  | patient | vital\_status | days\_to\_death | days\_to\_last\_follow\_up | Sur | Time | Groups |
| --- | --- | --- | --- | --- | --- | --- | --- |
|  | <chr> | <chr> | <int> | <int> | <lgl> | <dbl> | <lgl> |
| TCGA-E2-A15G-01A-11R-A12D-07 | TCGA-E2-A15G | Alive | NA | 554 | FALSE | 1.517808 | FALSE |
| TCGA-E2-A1B5-01A-21R-A12P-07 | TCGA-E2-A1B5 | Alive | NA | 984 | FALSE | 2.695890 | FALSE |
| TCGA-EW-A2FS-01A-11R-A17B-07 | TCGA-EW-A2FS | Alive | NA | 1604 | FALSE | 4.394521 | FALSE |
| TCGA-EW-A1P7-01A-21R-A144-07 | TCGA-EW-A1P7 | Alive | NA | 915 | FALSE | 2.506849 | FALSE |
| TCGA-LL-A5YO-01A-21R-A28M-07 | TCGA-LL-A5YO | Alive | NA | 440 | FALSE | 1.205479 | FALSE |
| TCGA-BH-A1FN-01A-11R-A13Q-07 | TCGA-BH-A1FN | Dead | 2192 | NA | TRUE | 6.005479 | TRUE |

In [50]:

```
dim(subset(sub.clinical, Groups == T & Time > 3 & vital_status == 'Dead'))[1] / dim(subset(sub.clinical, Groups == T & vital_status == 'Dead'))[1]
```

0.358208955223881

In [51]:

```
dim(subset(sub.clinical, Groups == F & Time > 3 & vital_status == 'Dead'))[1] / dim(subset(sub.clinical, Groups == F & vital_status == 'Dead'))[1]
```

0.644444444444444

In [52]:

```
roc.res.5 <- survivalROC(Stime = sub.clinical$Time, status = sub.clinical$Sur, marker = risk.socres, predict.time = 5, method = 'KM')
roc.res.3 <- survivalROC(Stime = sub.clinical$Time, status = sub.clinical$Sur, marker = risk.socres, predict.time = 3, method = 'KM')
roc.res.10 <- survivalROC(Stime = sub.clinical$Time, status = sub.clinical$Sur, marker = risk.socres, predict.time = 10, method = 'KM')
```

```
 2 records with missing values dropped. 

 2 records with missing values dropped. 

 2 records with missing values dropped.
```

In [53]:

```
pdf(file.path(out.figs.dir, 'roc.5vs3.years.pdf'), width = 8, height = 8)
plot(roc.res.5$FP, roc.res.5$TP, type = 'l', xlim = c(0, 1), ylim = c(0, 1), xlab = paste('1 - specificity'), ylab = 'Sensitivity', col = 'yellow', lwd = 2, cex.lab = 1.5, cex.axis = 1.5)
abline(0, 1, lwd = 2, lty = 'dashed')
box(lwd = 2)

par(new = TRUE)
plot(roc.res.3$FP, roc.res.3$TP, type = 'l', xlim = c(0, 1), ylim = c(0, 1), xlab = '', ylab = '', col = 'darkgreen', lwd = 2, xaxt = 'n', yaxt = 'n')
legend(0.35, 0.2, legend = c(sprintf('5-year ROC (AUC = %g)', round(roc.res.5$AUC, 3)), sprintf('3-year ROC (AUC = %g)', round(roc.res.3$AUC, 3))), col = c("yellow", "darkgreen"), lty = 1, cex = 1.5, lwd = 3, bty = 'n')
dev.off()
```

**png:** 2

### Univariate cox regression of 13 genes¶

In [54]:

```
exprs.sub <- exprs[, hub.genes.sub]
head(exprs.sub)[, 1 : 10]
```

A matrix: 6 × 10 of type dbl

|  | AF131215.8 | LINC01235 | LINC00987 | LINC00398 | LINC00667 | CTD\_2554C21.2 | ZNF888 | LL0XNC01\_237H1.2 | AC016995.3 | SFTA1P |
| --- | --- | --- | --- | --- | --- | --- | --- | --- | --- | --- |
| TCGA-E2-A15G-01A-11R-A12D-07 | 0.1270039 | 0.3151554 | 0.6017985 | 0.6327797 | 2.527675 | 1.9468146 | 1.2731744 | 1.0773688 | 0.2732504 | 0.3535274 |
| TCGA-E2-A1B5-01A-21R-A12P-07 | 0.3063988 | 0.9220029 | 1.5484011 | 0.1935985 | 1.779815 | 0.2372294 | 0.3825106 | 0.2789045 | 0.9050483 | 0.5616873 |
| TCGA-EW-A2FS-01A-11R-A17B-07 | 0.1862594 | 0.5951290 | 1.1854779 | 0.2378498 | 2.098742 | 0.6233850 | 0.8206887 | 0.4867726 | 0.5810859 | 0.4586515 |
| TCGA-EW-A1P7-01A-21R-A144-07 | 0.3759908 | 3.0392353 | 1.8106939 | 0.1448384 | 2.960917 | 2.6732052 | 0.9016777 | 0.4734871 | 2.4051563 | 0.6672513 |
| TCGA-LL-A5YO-01A-21R-A28M-07 | 0.2013102 | 2.2819006 | 0.4478722 | 0.1594376 | 2.788373 | 0.5363022 | 1.2295638 | 0.2471743 | 0.4428419 | 0.2670334 |
| TCGA-BH-A1FN-01A-11R-A13Q-07 | 0.2523297 | 1.2883921 | 0.3277982 | 0.1367585 | 2.211886 | 1.5844518 | 2.0234407 | 0.7448843 | 0.1062545 | 0.1943177 |

In [55]:

```
sub.clinical.expr <- cbind.data.frame(sub.clinical, exprs.sub)
```

In [56]:

```
fmt <- as.formula('Surv(Time, Sur)~Groups')
univ.models <- lapply(hub.genes.sub, function(x){ 
    sub.clinical.expr$Groups <- sub.clinical.expr[, x]
    res.cox <- OS.analysis(sub.clinical.expr, NULL, T, cutoff = T, prefix = NULL)
    sub.clinical.expr$Groups <- sub.clinical.expr$Groups <= res.cox$cut.off
    coxph(fmt, data = sub.clinical.expr)
})
```

In [57]:

```
names(univ.models) <- hub.genes.sub
```

In [58]:

```
univ.results <- lapply(univ.models, function(x){
    x <- summary(x)
    p.value <- signif(x$wald["pvalue"], digits = 2)
    HR <-signif(x$coef[2], digits = 2)
    lower.95 <- signif(x$conf.int[,"lower .95"], 2)
    upper.95 <- signif(x$conf.int[,"upper .95"], 2)
    conf <- paste0(HR, " (", lower.95, "-", upper.95, ")")
    res <- c(HR, lower.95, upper.95, conf, p.value)
    return(res)
}) %>% as.data.frame %>% t

rownames(univ.results) <- hub.genes.sub

colnames(univ.results) <- c('HR', 'lower.95', 'upper.95', 'conf', 'p.value')
plot.res <- cbind.data.frame(charac = rownames(univ.results), univ.results)

plot.res[, 2] <- as.numeric(plot.res[, 2]) %>% { log2(1 + .) }
plot.res[, 3] <- as.numeric(plot.res[, 3]) %>% { log2(1 + .) }
plot.res[, 4] <- as.numeric(plot.res[, 4]) %>% { log2(1 + .) }
plot.res[, 6] <- as.numeric(plot.res[, 6])
plot.res <- plot.res[order(plot.res$p.value), ]
plot.res['TGCT', 'upper.95'] <- 1

pdf(file.path(out.figs.dir, 'traning.hub.genes.forestplot.pdf'), width = 8, height = 10)
forestplot(
    plot.res[, c(1, 5, 6)],
    mean = plot.res[, 2],
    lower = plot.res[, 3],
    upper = plot.res[, 4],
    zero = 1,
    col=fpColors(box="darkblue", lines="black", zero = "gray50"),
    boxsize = 0.3,
    graph.pos = 2
)
dev.off()
```

**png:** 2

### Compare with other clinical features¶

In [1328]:

```
select.lab <- c('age_at_index', 'paper_BRCA_Subtype_PAM50', 'paper_pathologic_stage')
dat <- tcga.obj.sub@meta.data[ , select.lab]
dat <- cbind.data.frame(Group = ifelse(res.cox$group == 'High risk', 1, 0), dat)
dat$age_at_index <- as.numeric(dat$age_at_index)
dat$age_at_index <- ifelse(dat$age_at_index > 60, 1, 0)

dat <- subset(dat, !is.na(paper_BRCA_Subtype_PAM50))
dat$paper_BRCA_Subtype_PAM50 <- factor(dat$paper_BRCA_Subtype_PAM50, levels = c('Basal', 'LumA', 'LumB', 'Her2', 'Normal'))

dat <- subset(dat, paper_pathologic_stage != 'NA')
dat$paper_pathologic_stage  <- factor( dat$paper_pathologic_stage, levels = c("Stage_I", "Stage_II", "Stage_III", "Stage_IV"))

head(dat)
```

A data.frame: 6 × 4

|  | Group | age\_at\_index | paper\_BRCA\_Subtype\_PAM50 | paper\_pathologic\_stage |
| --- | --- | --- | --- | --- |
|  | <dbl> | <dbl> | <fct> | <fct> |
| TCGA-E2-A15G-01A-11R-A12D-07 | 0 | 1 | LumA | Stage\_II |
| TCGA-E2-A1B5-01A-21R-A12P-07 | 0 | 0 | LumA | Stage\_II |
| TCGA-EW-A2FS-01A-11R-A17B-07 | 0 | 0 | LumA | Stage\_II |
| TCGA-EW-A1P7-01A-21R-A144-07 | 0 | 0 | Normal | Stage\_II |
| TCGA-LL-A5YO-01A-21R-A28M-07 | 0 | 0 | Her2 | Stage\_I |
| TCGA-BH-A1FN-01A-11R-A13Q-07 | 1 | 0 | LumB | Stage\_II |

In [1329]:

```
select.lab <- c('age_at_index', 'paper_BRCA_Subtype_PAM50', 'paper_pathologic_stage')
dat <- tcga.obj.sub@meta.data[ , select.lab]
dat <- cbind.data.frame(Group = ifelse(res.cox$group == 'High risk', 1, 0), dat)
dat$age_at_index <- as.numeric(dat$age_at_index)
dat$age_at_index <- ifelse(dat$age_at_index > 60, 1, 0)

dat <- subset(dat, !is.na(paper_BRCA_Subtype_PAM50))
dat$paper_BRCA_Subtype_PAM50 <- factor(dat$paper_BRCA_Subtype_PAM50, levels = c('Basal', 'LumA', 'LumB', 'Her2', 'Normal'))

dat <- subset(dat, paper_pathologic_stage != 'NA')
dat$paper_pathologic_stage  <- factor( dat$paper_pathologic_stage, levels = c("Stage_I", "Stage_II", "Stage_III", "Stage_IV"))

colnames(dat) <- c('LincSig', 'Age', 'PAM50', 'Stage')

dat.new <- cbind.data.frame(sub.clinical[rownames(dat), ], dat)
formula.fmt <- as.formula(paste0('Surv(Time, Sur)~', paste(colnames(dat), sep = ' ', collapse = '+')))

mult.var.cox <- coxph(formula.fmt, data = dat.new)
ggforest(mult.var.cox, data = dat.new, fontsize = 1)

ggsave(file.path(out.figs.dir, 'ggforest_riskscore_vs_others.pdf'), width = 8, height = 8)
saveRDS(mult.var.cox, file = file.path(out.data.dir, 'mult.var.cox.clini.charac.rds'))
```

In [1330]:

```
head(dat.new)
```

A data.frame: 6 × 11

|  | patient | vital\_status | days\_to\_death | days\_to\_last\_follow\_up | Sur | Time | Groups | LincSig | Age | PAM50 | Stage |
| --- | --- | --- | --- | --- | --- | --- | --- | --- | --- | --- | --- |
|  | <chr> | <chr> | <int> | <int> | <lgl> | <dbl> | <dbl> | <dbl> | <dbl> | <fct> | <fct> |
| TCGA-E2-A15G-01A-11R-A12D-07 | TCGA-E2-A15G | Alive | NA | 554 | FALSE | 1.517808 | 0.2203707 | 0 | 1 | LumA | Stage\_II |
| TCGA-E2-A1B5-01A-21R-A12P-07 | TCGA-E2-A1B5 | Alive | NA | 984 | FALSE | 2.695890 | -1.1014146 | 0 | 0 | LumA | Stage\_II |
| TCGA-EW-A2FS-01A-11R-A17B-07 | TCGA-EW-A2FS | Alive | NA | 1604 | FALSE | 4.394521 | -0.3787754 | 0 | 0 | LumA | Stage\_II |
| TCGA-EW-A1P7-01A-21R-A144-07 | TCGA-EW-A1P7 | Alive | NA | 915 | FALSE | 2.506849 | -0.8431213 | 0 | 0 | Normal | Stage\_II |
| TCGA-LL-A5YO-01A-21R-A28M-07 | TCGA-LL-A5YO | Alive | NA | 440 | FALSE | 1.205479 | 0.6170595 | 0 | 0 | Her2 | Stage\_I |
| TCGA-BH-A1FN-01A-11R-A13Q-07 | TCGA-BH-A1FN | Dead | 2192 | NA | TRUE | 6.005479 | 1.7747969 | 1 | 0 | LumB | Stage\_II |

In [1331]:

```
univ.models <- lapply(c('LincSig', 'Age', 'PAM50', 'Stage'), function(x){ 
    fmt <- as.formula(paste0('Surv(Time, Sur)~', x))
    coxph(fmt, data = dat.new) %>% summary
})      
names(univ.models) <- c('LincSig', 'Age', 'PAM50', 'Stage')
saveRDS(univ.models, file = file.path(out.data.dir, 'univ.clinical.models.rds'))
```

In [1332]:

```
univ.models
```

```
$LincSig
Call:
coxph(formula = fmt, data = dat.new)

  n= 1065, number of events= 141 
   (因为不存在，1个观察量被删除了)

          coef exp(coef) se(coef)     z Pr(>|z|)    
LincSig 2.0585    7.8341   0.1781 11.56   <2e-16 ***
---
Signif. codes:  0 '***' 0.001 '**' 0.01 '*' 0.05 '.' 0.1 ' ' 1

        exp(coef) exp(-coef) lower .95 upper .95
LincSig     7.834     0.1276     5.526     11.11

Concordance= 0.694  (se = 0.024 )
Likelihood ratio test= 111.2  on 1 df,   p=<2e-16
Wald test            = 133.6  on 1 df,   p=<2e-16
Score (logrank) test = 184.6  on 1 df,   p=<2e-16


$Age
Call:
coxph(formula = fmt, data = dat.new)

  n= 1065, number of events= 141 
   (因为不存在，1个观察量被删除了)

      coef exp(coef) se(coef)     z Pr(>|z|)    
Age 0.8197    2.2699   0.1720 4.766 1.88e-06 ***
---
Signif. codes:  0 '***' 0.001 '**' 0.01 '*' 0.05 '.' 0.1 ' ' 1

    exp(coef) exp(-coef) lower .95 upper .95
Age      2.27     0.4405      1.62      3.18

Concordance= 0.62  (se = 0.023 )
Likelihood ratio test= 22.94  on 1 df,   p=2e-06
Wald test            = 22.71  on 1 df,   p=2e-06
Score (logrank) test = 23.98  on 1 df,   p=1e-06


$PAM50
Call:
coxph(formula = fmt, data = dat.new)

  n= 1065, number of events= 141 
   (因为不存在，1个观察量被删除了)

               coef exp(coef) se(coef)      z Pr(>|z|)  
PAM50LumA   -0.3173    0.7281   0.2326 -1.364    0.172  
PAM50LumB    0.2059    1.2286   0.2641  0.779    0.436  
PAM50Her2    0.5431    1.7213   0.3102  1.751    0.080 .
PAM50Normal  0.1413    1.1518   0.4247  0.333    0.739  
---
Signif. codes:  0 '***' 0.001 '**' 0.01 '*' 0.05 '.' 0.1 ' ' 1

            exp(coef) exp(-coef) lower .95 upper .95
PAM50LumA      0.7281     1.3734    0.4616     1.149
PAM50LumB      1.2286     0.8139    0.7321     2.062
PAM50Her2      1.7213     0.5810    0.9371     3.162
PAM50Normal    1.1518     0.8682    0.5010     2.648

Concordance= 0.594  (se = 0.028 )
Likelihood ratio test= 11.66  on 4 df,   p=0.02
Wald test            = 12.28  on 4 df,   p=0.02
Score (logrank) test = 12.74  on 4 df,   p=0.01


$Stage
Call:
coxph(formula = fmt, data = dat.new)

  n= 1065, number of events= 141 
   (因为不存在，1个观察量被删除了)

                  coef exp(coef) se(coef)     z Pr(>|z|)    
StageStage_II   0.4741    1.6066   0.2785 1.703 0.088645 .  
StageStage_III  1.1009    3.0069   0.2924 3.765 0.000166 ***
StageStage_IV   2.4839   11.9875   0.3674 6.761 1.37e-11 ***
---
Signif. codes:  0 '***' 0.001 '**' 0.01 '*' 0.05 '.' 0.1 ' ' 1

               exp(coef) exp(-coef) lower .95 upper .95
StageStage_II      1.607    0.62242    0.9308     2.773
StageStage_III     3.007    0.33257    1.6953     5.333
StageStage_IV     11.987    0.08342    5.8346    24.629

Concordance= 0.677  (se = 0.026 )
Likelihood ratio test= 46.04  on 3 df,   p=6e-10
Wald test            = 60.4  on 3 df,   p=5e-13
Score (logrank) test = 78.98  on 3 df,   p=<2e-16
```

### Rank score from low to high, and visualized by bar and scatter plots¶

In [1333]:

```
sub.clinical.scores <- cbind.data.frame(sub.clinical, Score = t(risk.socres), Class = res.cox$group)
sub.clinical.scores.ordered <- sub.clinical.scores[order(sub.clinical.scores$Score), ]
head(sub.clinical.scores.ordered)
```

A data.frame: 6 × 9

|  | patient | vital\_status | days\_to\_death | days\_to\_last\_follow\_up | Sur | Time | Groups | Score | Class |
| --- | --- | --- | --- | --- | --- | --- | --- | --- | --- |
|  | <chr> | <chr> | <int> | <int> | <lgl> | <dbl> | <dbl> | <dbl> | <chr> |
| TCGA-A7-A13D-01B-04R-A277-07 | TCGA-A7-A13D | Alive | NA | 965 | FALSE | 2.643836 | -4.041655 | -4.041655 | Low risk |
| TCGA-A7-A26J-01B-02R-A277-07 | TCGA-A7-A26J | Alive | NA | 627 | FALSE | 1.717808 | -4.013849 | -4.013849 | Low risk |
| TCGA-E9-A22G-01A-11R-A157-07 | TCGA-E9-A22G | Alive | NA | 1239 | FALSE | 3.394521 | -3.502837 | -3.502837 | Low risk |
| TCGA-A2-A04U-01A-11R-A115-07 | TCGA-A2-A04U | Alive | NA | 2654 | FALSE | 7.271233 | -3.295690 | -3.295690 | Low risk |
| TCGA-B6-A0I2-01A-11R-A034-07 | TCGA-B6-A0I2 | Alive | NA | 4361 | FALSE | 11.947945 | -3.145192 | -3.145192 | Low risk |
| TCGA-AR-A0U4-01A-11R-A109-07 | TCGA-AR-A0U4 | Alive | NA | 3261 | FALSE | 8.934247 | -2.790738 | -2.790738 | Low risk |

In [1334]:

```
sub.clinical.scores.ordered$patient <- rownames(sub.clinical.scores.ordered)
sub.clinical.scores.ordered$patient <- factor(sub.clinical.scores.ordered$patient, level = sub.clinical.scores.ordered$patient)
sub.clinical.scores.ordered <- sub.clinical.scores.ordered[!is.na(sub.clinical.scores.ordered$Sur), ]
```

In [1335]:

```
sub.clinical.scores.ordered$patient <- factor(sub.clinical.scores.ordered$patient, level = sub.clinical.scores.ordered$patient)
cols <- PAIRWISE_COLORS %>% unlist
names(cols) <- c('High risk', 'Low risk')
gp1 <- ggplot(
    data = sub.clinical.scores.ordered, 
    aes(x = patient, y = Score, fill = Class)) + 
    geom_bar(stat = 'identity') + 
    theme_bw(base_size = 20) + 
    theme(
        panel.border = element_blank(),  
        panel.grid.major = element_blank(),
        panel.grid.minor = element_blank(), 
        axis.text.x = element_blank(), 
        axis.line.x = element_line(),
        axis.title.x = element_blank(),
        axis.ticks.x = element_blank(),
        axis.line.y = element_line(colour = 'black')
    ) + scale_fill_manual(values = cols)

cols <- c('red', 'darkgreen')
names(cols) <- c('Dead', 'Alive')
gp2 <- ggplot(
    data = sub.clinical.scores.ordered, 
    aes(x = patient, y = Time, color = vital_status)) + 
    geom_point(size = 3, alpha = 0.5) + 
    theme_bw(base_size = 20) + 
    theme(
        panel.border = element_blank(),  
        panel.grid.major = element_blank(),
        panel.grid.minor = element_blank(), 
        axis.text.x = element_blank(), 
        axis.line.x = element_line(),
        axis.ticks.x=element_blank(),
        axis.title.x = element_blank(),
        axis.line.y = element_line(colour = 'black')
    ) + scale_color_manual(values = cols)
ggarrange(gp1, gp2, nrow = 2) 
ggsave(file.path(out.figs.dir, 'risk.score.ordered.pdf'), height = 6, width = 8)
```

### Expression analysis of LincSigs¶

In [875]:

```
expr.pf <- FetchData(tcga.obj.sub, vars = gsub('_', '-', hub.genes.sub)) %>% { log2(1 + .) }
expr.pf <- cbind.data.frame(expr.pf, Group = tcga.obj.sub@meta.data$sample_type)
expr.pf[rownames(sub.clinical), 'Group'] <- res.cox$group
dim(expr.pf)
```

1. 1109
2. 30

In [876]:

```
table(expr.pf$Group)
expr.pf <- tidyr::gather(expr.pf, 'Gene', 'Expr', -Group)
```

```
High risk  Low risk 
      179       930
```

In [877]:

```
head(expr.pf)
expr.pf$Gene <- factor(expr.pf$Gene, levels = gsub('_', '-', hub.genes.sub))
```

A data.frame: 6 × 3

|  | Group | Gene | Expr |
| --- | --- | --- | --- |
|  | <chr> | <chr> | <dbl> |
| 1 | Low risk | AF131215.8 | 0.1270039 |
| 2 | Low risk | AF131215.8 | 0.3063988 |
| 3 | Low risk | AF131215.8 | 0.1862594 |
| 4 | Low risk | AF131215.8 | 0.3759908 |
| 5 | Low risk | AF131215.8 | 0.2013102 |
| 6 | High risk | AF131215.8 | 0.2523297 |

In [879]:

```
options(repr.plot.width = 20, repr.plot.height = 8)
cols <- unlist(PAIRWISE_COLORS)
names(cols) <- c('High risk', 'Low risk')
ggplot(data = expr.pf, aes(x = Gene, y = Expr, fill = Group)) + 
    geom_boxplot(outlier.shape = NA) + 
    theme_classic(base_size = 20) + 
    theme(axis.text.x = element_text(angle = 45, vjust = 1, hjust = 1),legend.position="top") + 
    scale_fill_manual(values = cols) + ylab('log2(1 + TPM)') + xlab('') +
    stat_compare_means(aes(group = Group), label = "p.signif")
ggsave(file.path(out.figs.dir, 'expr.lincgenes.boxplot.pdf'), width = 12, height = 6)
```

In [1340]:

```
tcga.obj@meta.data$sample_type %>% unique
```

1. 'Primary Tumor'
2. 'Metastatic'
3. 'Solid Tissue Normal'

In [1341]:

```
expr.pf <- FetchData(tcga.obj, vars = gsub('_', '-', hub.genes.sub)) %>% { log2(1 + .) }
expr.pf <- cbind.data.frame(expr.pf, Group = ifelse(tcga.obj@meta.data$sample_type == 'Solid Tissue Normal', 'Normal', 'Tumor'))

dim(expr.pf)
table(expr.pf$Group)
expr.pf <- tidyr::gather(expr.pf, 'Gene', 'Expr', -Group)

head(expr.pf)
expr.pf$Gene <- factor(expr.pf$Gene, levels = gsub('_', '-', hub.genes.sub))
```

1. 1222
2. 26

```
Normal  Tumor 
   113   1109
```

A data.frame: 6 × 3

|  | Group | Gene | Expr |
| --- | --- | --- | --- |
|  | <chr> | <chr> | <dbl> |
| 1 | Tumor | LINC01235 | 0.3151554 |
| 2 | Tumor | LINC01235 | 0.9220029 |
| 3 | Tumor | LINC01235 | 0.5951290 |
| 4 | Tumor | LINC01235 | 3.0392353 |
| 5 | Tumor | LINC01235 | 2.2819006 |
| 6 | Tumor | LINC01235 | 1.2883921 |

In [1342]:

```
expr.pf$Group <- factor(expr.pf$Group, levels = c('Tumor', 'Normal'))
options(repr.plot.width = 20, repr.plot.height = 8)
cols <- unlist(PAIRWISE_COLORS)
names(cols) <- c('Tumor', 'Normal')
ggplot(data = expr.pf, aes(x = Gene, y = Expr, fill = Group)) + 
    geom_boxplot(outlier.shape = NA) + 
    theme_classic(base_size = 20) + 
    theme(axis.text.x = element_text(angle = 45, vjust = 1, hjust = 1),legend.position="top") + 
    scale_fill_manual(values = cols) + ylab('log2(1 + TPM)') + xlab('') +
    stat_compare_means(aes(group = Group), label = "p.signif")
ggsave(file.path(out.figs.dir, 'expr.lincgenes.normal.vs.tumor.boxplot.pdf'), width = 10, height = 6)
```

In [1343]:

```
exhau.expr <- FetchData(tcga.obj.sub, vars = c('CD247', 'PDCD1', 'CTLA4', 'HAVCR2', 'LAG3'))
head(exhau.expr)
```

A data.frame: 6 × 5

|  | CD247 | PDCD1 | CTLA4 | HAVCR2 | LAG3 |
| --- | --- | --- | --- | --- | --- |
|  | <dbl> | <dbl> | <dbl> | <dbl> | <dbl> |
| TCGA-E2-A15G-01A-11R-A12D-07 | 0.2688011 | 0.0923282 | 0.04091862 | 3.392136 | 0.2341841 |
| TCGA-E2-A1B5-01A-21R-A12P-07 | 14.4774895 | 3.0686221 | 3.68244612 | 5.819615 | 3.2236322 |
| TCGA-EW-A2FS-01A-11R-A17B-07 | 1.3766969 | 1.4287495 | 1.34810875 | 2.480183 | 1.4346915 |
| TCGA-EW-A1P7-01A-21R-A144-07 | 8.0310264 | 2.7789670 | 2.72597001 | 4.844353 | 2.4295641 |
| TCGA-LL-A5YO-01A-21R-A28M-07 | 10.7730938 | 10.7545282 | 7.52808707 | 10.144256 | 9.1889358 |
| TCGA-BH-A1FN-01A-11R-A13Q-07 | 0.2679786 | 0.1917619 | 0.44617809 | 5.050139 | 1.1791293 |

In [1344]:

```
exh.pf <- cbind.data.frame(exhau.expr, Score = as.vector(risk.socres))
exh.pf <- tidyr::gather(exh.pf, 'Gene', 'Expr', -Score)
exh.pf$Gene <- factor(exh.pf$Gene, levels = c('CD247', 'PDCD1', 'CTLA4', 'HAVCR2', 'LAG3'))
head(exh.pf)
```

A data.frame: 6 × 3

|  | Score | Gene | Expr |
| --- | --- | --- | --- |
|  | <dbl> | <fct> | <dbl> |
| 1 | 0.2203707 | CD247 | 0.2688011 |
| 2 | -1.1014146 | CD247 | 14.4774895 |
| 3 | -0.3787754 | CD247 | 1.3766969 |
| 4 | -0.8431213 | CD247 | 8.0310264 |
| 5 | 0.6170595 | CD247 | 10.7730938 |
| 6 | 1.7747969 | CD247 | 0.2679786 |

In [1345]:

```
gpp <- ggplot(exh.pf, aes(x = scale(log2(1 + Expr)), y = scale(Score), color = Gene)) + geom_point() + facet_wrap(~Gene, ncol = 5) + geom_smooth(method = "lm", se = T, color = 'black', lwd = 1.5) + stat_cor(method="pearson")  + theme_bw(base_size = 20) + theme(legend.position = 'none') +
xlab('Scaled expression') + ylab('Scaled risk score')
gpp
```

```
`geom_smooth()` using formula 'y ~ x'
```

In [1346]:

```
exh.pf <- cbind.data.frame(exhau.expr, Class = res.cox$group)
exh.pf <- tidyr::gather(exh.pf, 'Gene', 'Expr', -Class)
exh.pf$Gene <- factor(exh.pf$Gene, levels = c('CD247', 'PDCD1', 'CTLA4', 'HAVCR2', 'LAG3'))
head(exh.pf)
```

A data.frame: 6 × 3

|  | Class | Gene | Expr |
| --- | --- | --- | --- |
|  | <chr> | <fct> | <dbl> |
| 1 | Low risk | CD247 | 0.2688011 |
| 2 | Low risk | CD247 | 14.4774895 |
| 3 | Low risk | CD247 | 1.3766969 |
| 4 | Low risk | CD247 | 8.0310264 |
| 5 | Low risk | CD247 | 10.7730938 |
| 6 | High risk | CD247 | 0.2679786 |

In [1347]:

```
gpp2 <- ggplot(exh.pf, aes(x = Class, y = log2(1 + Expr), fill = Gene)) + geom_boxplot() + facet_wrap(~Gene, ncol = 5) + stat_compare_means(aes(group = Class), label = "p.format") + theme_bw(base_size = 20) + theme(legend.position = 'none') + xlab('')
gpp2
```

In [1348]:

```
ggarrange(gpp, gpp2, nrow = 2)
```

```
`geom_smooth()` using formula 'y ~ x'
```

In [1349]:

```
ggsave(file.path(out.figs.dir, 'exhuas.risk.groups.pdf'), width = 14, height = 7)
```

### Classified by immune patterns¶

In [1440]:

```
sub.clinical$Class <- res.cox$group
sub.clinical.df <- cbind.data.frame(sub.clinical, exhau.expr)
```

In [1441]:

```
for (gene in colnames(exhau.expr)) {
    tmp.set.1 <- subset(sub.clinical.df, Class == 'Low risk')
    tmp.set.1[tmp.set.1[, gene] <= median(sub.clinical.df[, gene]), 'Groups'] <- 'C1'
    tmp.set.1[tmp.set.1[, gene] > median(sub.clinical.df[, gene]), 'Groups'] <- 'C2'
    
    tmp.set.2 <- subset(sub.clinical.df, Class == 'High risk')
    tmp.set.2[tmp.set.2[, gene] <= median(sub.clinical.df[, gene]), 'Groups'] <- 'C3'
    tmp.set.2[tmp.set.2[, gene] > median(sub.clinical.df[, gene]), 'Groups'] <- 'C4'
    
    clin.tmp <- rbind.data.frame(tmp.set.1, tmp.set.2)
    options(repr.plot.width = 10, repr.plot.height = 6)
    res.cox.1 <- OS.analysis(
        clin.tmp, 
        c('#8A0E1A','green', '#3574CE', '#ED8141'), 
        F, 
        cutoff = F, 
        prefix = sprintf('TCGA_%s_OS_all.pdf', gene),
        out.figs.dir = out.figs.dir,
        title = sprintf('LinSig + %s', gene)
    )
}
```

In [51]:

```
tcga.obj.sub@meta.data$riskScore <- as.vector(risk.socres)
tcga.obj.sub@meta.data$Group <- res.cox$group
```

In [1437]:

```
options(repr.plot.width = 15, repr.plot.height = 6)
plot.df <- c()
Age.df <- rbind(
    cbind(tcga.obj.sub@meta.data[, c('riskScore', 'age_at_index')] %>% as.matrix, Sample = rep('TCGA', dim(tcga.obj.sub)[2]))
) %>% as.data.frame

Age.df$Class <- 'Age'
colnames(Age.df) <- c('Risk', 'Type', 'Sample', 'Class')
Age.df$Type <- ifelse(as.numeric(Age.df$Type) >= 60, '>=60', '<60')
Age.df$Risk <- as.numeric(Age.df$Risk)
Age.df <- subset(Age.df, !is.na(Type))
plot.df <- rbind.data.frame(plot.df, Age.df)

pam50.df <- rbind(
    cbind(tcga.obj.sub@meta.data[, c('riskScore', 'paper_BRCA_Subtype_PAM50')] %>% as.matrix, Sample = rep('TCGA', dim(tcga.obj.sub)[2]))
) %>% as.data.frame

pam50.df$Class <- 'PAM50'
colnames(pam50.df) <- c('Risk', 'Type', 'Sample', 'Class')
pam50.df$Risk <- as.numeric(pam50.df$Risk)
pam50.df <- subset(pam50.df, Type %in% c('LumA', 'LumB', 'Her2', 'Basal', 'Normal'))
plot.df <- rbind.data.frame(plot.df, pam50.df)

tcga.obj.sub@meta.data$stage_novel <- tcga.obj.sub@meta.data$tumor_stage
tcga.obj.sub@meta.data$stage_novel[tcga.obj.sub@meta.data$stage_novel %in% c('stage ia', 'stage i', 'stage ib')] <- '1'
tcga.obj.sub@meta.data$stage_novel[tcga.obj.sub@meta.data$stage_novel %in% c('stage ii', 'stage iib', 'stage iia')] <- '2'
tcga.obj.sub@meta.data$stage_novel[tcga.obj.sub@meta.data$stage_novel %in% c('stage iii', 'stage iiib', 'stage iiia', 'stage iiic')] <- '3'
tcga.obj.sub@meta.data$stage_novel[tcga.obj.sub@meta.data$stage_novel %in% c('stage iv')] <- '4'
tcga.obj.sub@meta.data$stage_novel[tcga.obj.sub@meta.data$stage_novel %in% c('stage x')] <- '5'

stage.df <- rbind(
    cbind(tcga.obj.sub@meta.data[, c('riskScore', 'stage_novel')] %>% as.matrix, Sample = rep('TCGA', dim(tcga.obj.sub)[2]))
) %>% as.data.frame

stage.df$Class <- 'Stage'
colnames(stage.df) <- c('Risk', 'Type', 'Sample', 'Class')
stage.df$Risk <- as.numeric(stage.df$Risk)
stage.df <- subset(stage.df, !(Type %in% c('not reported', '5')))
stage.df$Type[stage.df$Type == '1'] <- 'I'
stage.df$Type[stage.df$Type == '2'] <- 'II'
stage.df$Type[stage.df$Type == '3'] <- 'III'
stage.df$Type[stage.df$Type == '4'] <- 'IV'
stage.df <- subset(stage.df, !is.na(Type))

plot.df <- rbind.data.frame(plot.df, stage.df)
Pan.Gyn.df <- rbind(
    cbind(tcga.obj.sub@meta.data[, c('riskScore', 'paper_Pan.Gyn.Clusters')] %>% as.matrix, Sample = rep('TCGA', dim(tcga.obj.sub)[2]))
) %>% as.data.frame

Pan.Gyn.df$Class <- 'Pan.Gyn'
colnames(Pan.Gyn.df) <- c('Risk', 'Type', 'Sample', 'Class')
Pan.Gyn.df$Risk <- as.numeric(Pan.Gyn.df$Risk)
Pan.Gyn.df <- subset(Pan.Gyn.df, Type %in% c('C1', 'C2', 'C3', 'C4', 'C5'))
plot.df <- rbind.data.frame(plot.df, Pan.Gyn.df)

plot.df$Class <- factor(plot.df$Class, levels = c('Age', "Stage", "PAM50", "Pan.Gyn"))
plot.df$Type <- factor(plot.df$Type, levels = c(">=60", "<60", "Normal", "LumA", "LumB", "Her2", "Basal", "I","II","III","IV", "C1", "C2", "C3", "C4", "C5"))

my.comparisons <- list(
    c(">=60", "<60"),    
    c("I","II","III","IV"),
    c("Normal", "LumA", "LumB", "Her2", "Basal"),
    c("C1", "C2", "C3", "C4", "C5")
)
ptest <- plot.df %>% group_by(Class) %>% summarize(p.value = kruskal.test(Risk ~Type)$p.value)
ggplot(data = plot.df, aes(x = Type, y = Risk, fill = Class)) +
    geom_boxplot() +
    theme_bw(base_size = 20) +
    theme_classic()+
    theme(axis.text.x = element_text(angle = 45, vjust = 1, hjust=1), legend.position="top") + stat_compare_means(aes(group = Type), label = "p.signif") + guides(fill = guide_legend(ncol=4))
ptest
ggsave(file.path(out.figs.dir, 'risk_score_groups_boxplot.pdf'), width = 10, height = 4)
```

A tibble: 4 × 2

| Class | p.value |
| --- | --- |
| <fct> | <dbl> |
| Age | 8.459279e-04 |
| Stage | 5.047830e-05 |
| PAM50 | 3.090810e-11 |
| Pan.Gyn | 8.151925e-04 |

In [1354]:

```
group.order <- rbind(
    cbind(tcga.obj.sub@meta.data[, c('Group', 'paper_BRCA_Subtype_PAM50')] %>% as.matrix, Sample = rep('TCGA', dim(tcga.obj.sub)[2]))
) %>% as.data.frame

group.order$Class <- 'PAM50'
colnames(group.order) <- c('Risk', 'Type', 'Sample', 'Class')
pam50.group <- subset(group.order, Type %in% c('LumA', 'LumB', 'Her2', 'Basal', 'Normal'))

aa <- table(pam50.group[, 1], pam50.group[, 2]) %>% t
bb <- sweep(aa, 2, colSums(aa), '/')[names(PAM50_COLOR), ]

options(repr.plot.width = 6, repr.plot.height = 8)
pdf(file.path(out.figs.dir, 'Risk_pam50_percent.bar.pdf'), width = 3, height = 5)
tmp <- barplot(bb, col = PAM50_COLOR %>% unlist, ylab = 'Percentage', cex.axis = 2, cex.names = 2, las = 2)
legend(1,1, legend = names(PAM50_COLOR), col=PAM50_COLOR %>% unlist, lwd=3, cex=1, lty = 1, ncol = 2)
dev.off()

barplot(bb, col = PAM50_COLOR %>% unlist, ylab = 'Percentage', cex.axis = 2, cex.names = 2, las = 2)
```

**png:** 2

In [1442]:

```
source('IPS.R')
ips.score <- IPSCore(GetAssayData(tcga.obj.sub) %>% as.data.frame %>% { log2(1 + .) })
```

```
载入程辑包：'gridExtra'


The following object is masked from 'package:dplyr':

    combine
```

```
differently named or missing genes:  CCL3L1
```

In [1469]:

```
head(ips.score)
```

A data.frame: 6 × 6

|  | SAMPLE | MHC | EC | SC | CP | AZ |
| --- | --- | --- | --- | --- | --- | --- |
|  | <chr> | <dbl> | <dbl> | <dbl> | <dbl> | <dbl> |
| 1 | TCGA-E2-A15G-01A-11R-A12D-07 | 3.076520 | 0.7745970 | -0.894677 | 0.12401948 | 3.080459 |
| 2 | TCGA-E2-A1B5-01A-21R-A12P-07 | 4.584694 | 1.5202322 | -1.592181 | -0.50256740 | 4.010178 |
| 3 | TCGA-EW-A2FS-01A-11R-A17B-07 | 3.510237 | 1.0770778 | -1.011054 | -0.01773302 | 3.558528 |
| 4 | TCGA-EW-A1P7-01A-21R-A144-07 | 4.803366 | 1.5052308 | -1.781104 | -0.47121319 | 4.056279 |
| 5 | TCGA-LL-A5YO-01A-21R-A28M-07 | 4.734896 | 1.5658861 | -1.977480 | -0.82551720 | 3.497785 |
| 6 | TCGA-BH-A1FN-01A-11R-A13Q-07 | 3.693526 | 0.9706054 | -1.209145 | -0.24538344 | 3.209602 |

In [1505]:

```
plot.df.ips <- cbind.data.frame(IPScore = ips.score[, 6], riskScore = res.cox$group)
cols <- unlist(PAIRWISE_COLORS)
names(cols) <- c('High risk', 'Low risk')
gp.1 <- ggplot(plot.df.ips, aes(x = riskScore, y = IPScore, fill = riskScore)) + geom_boxplot() + stat_compare_means(label = "p.signif") + theme_classic(base_size = 20) + scale_fill_manual(values = cols) +  theme(legend.position = 'none') + xlab('')
gp.1
```

In [1503]:

```
plot.df.ips <- cbind.data.frame(IPScore = ips.score[, 6], riskScore = as.vector(risk.socres))
gp.2 <- ggplot(plot.df.ips, aes(x = riskScore, y = IPScore)) + geom_point(size = 3, alpha = 0.5, color = 'blue')+ geom_smooth(method = "lm", se = T, color = 'red', lwd = 1.5) + stat_cor(method="pearson") + theme_classic(base_size = 20) + theme(legend.position = 'none')
gp.2
```

```
`geom_smooth()` using formula 'y ~ x'
```

In [1504]:

```
ggarrange(gp.1, gp.2, nrow = 2) 
ggsave(file.path(out.figs.dir, 'IPS.risk.score.pdf'), height = 6, width = 4)
```

```
`geom_smooth()` using formula 'y ~ x'
```

In [1508]:

```
### NES score of cancer pathways
```

In [462]:

```
nes.score.sub <- nes.score[colnames(tcga.obj.sub), ]
```

In [463]:

```
dim(nes.score.sub)
head(nes.score.sub)
```

1. 1109
2. 10

A matrix: 6 × 10 of type dbl

|  | RTK\_RAS | NOTCH | HIPPO | WNT | PI3K | CELL\_CYCLE | TGF\_Beta | MYC | TP53 | NRF2 |
| --- | --- | --- | --- | --- | --- | --- | --- | --- | --- | --- |
| TCGA-E2-A15G-01A-11R-A12D-07 | 1.336675 | 1.240839 | 1.192835 | 1.186799 | 1.521849 | 1.356145 | 1.478386 | 1.417893 | 1.419021 | 1.737924 |
| TCGA-E2-A1B5-01A-21R-A12P-07 | 1.313803 | 1.267868 | 1.189044 | 1.260562 | 1.453758 | 1.475970 | 1.470507 | 1.449694 | 1.460431 | 1.694040 |
| TCGA-EW-A2FS-01A-11R-A17B-07 | 1.352823 | 1.248907 | 1.171422 | 1.205154 | 1.420731 | 1.500784 | 1.443278 | 1.347984 | 1.438744 | 1.662352 |
| TCGA-EW-A1P7-01A-21R-A144-07 | 1.396801 | 1.311661 | 1.327573 | 1.349573 | 1.428941 | 1.588284 | 1.447075 | 1.425700 | 1.467098 | 1.670157 |
| TCGA-LL-A5YO-01A-21R-A28M-07 | 1.260467 | 1.279847 | 1.182847 | 1.218694 | 1.414804 | 1.546093 | 1.354670 | 1.407807 | 1.400502 | 1.674053 |
| TCGA-BH-A1FN-01A-11R-A13Q-07 | 1.343863 | 1.314693 | 1.287907 | 1.161825 | 1.474510 | 1.539289 | 1.512237 | 1.495506 | 1.476215 | 1.692673 |

In [464]:

```
nes.score.sub.plot <- cbind.data.frame(nes.score.sub %>% scale, Group = ifelse(res.cox$group == 'High risk', 'High', 'Low'))
nes.score.sub.plot <- tidyr::gather(nes.score.sub.plot, 'Pathway', 'NES',  -Group)
nes.score.sub.plot$Pathway <- factor(nes.score.sub.plot$Pathway, levels = colnames(nes.score.sub))
nes.score.sub.plot$Group <- factor(nes.score.sub.plot$Group, levels = c('High', 'Low'))
head(nes.score.sub.plot)
```

A data.frame: 6 × 3

|  | Group | Pathway | NES |
| --- | --- | --- | --- |
|  | <fct> | <fct> | <dbl> |
| 1 | Low | RTK\_RAS | -0.4137421 |
| 2 | Low | RTK\_RAS | -0.7883398 |
| 3 | Low | RTK\_RAS | -0.1492681 |
| 4 | Low | RTK\_RAS | 0.5710126 |
| 5 | Low | RTK\_RAS | -1.6618908 |
| 6 | High | RTK\_RAS | -0.2960191 |

In [465]:

```
cols
```

High
:   '#F9C996'

Low
:   '#90B4C6'

In [466]:

```
options(repr.plot.width = 15, repr.plot.height = 6)
names(cols) <- c('High', 'Low')
ggplot(nes.score.sub.plot, aes(x = Pathway, y = NES, fill = Group)) + geom_boxplot(outlier.shape = NA) + stat_compare_means(label = "p.signif") + theme_classic(base_size = 20) + scale_fill_manual(values = cols) + ylim(-3, 3) + theme(axis.text.x = element_text(angle = 45, vjust = 1, hjust=1), legend.position="top") + xlab('')
ggsave(file.path(out.figs.dir, 'nes.high.vs.low.groups.pathways.pdf'), height = 6, width = 10)
```

In [469]:

```
head(nes.score.sub)
```

A matrix: 6 × 10 of type dbl

|  | RTK\_RAS | NOTCH | HIPPO | WNT | PI3K | CELL\_CYCLE | TGF\_Beta | MYC | TP53 | NRF2 |
| --- | --- | --- | --- | --- | --- | --- | --- | --- | --- | --- |
| TCGA-E2-A15G-01A-11R-A12D-07 | 1.336675 | 1.240839 | 1.192835 | 1.186799 | 1.521849 | 1.356145 | 1.478386 | 1.417893 | 1.419021 | 1.737924 |
| TCGA-E2-A1B5-01A-21R-A12P-07 | 1.313803 | 1.267868 | 1.189044 | 1.260562 | 1.453758 | 1.475970 | 1.470507 | 1.449694 | 1.460431 | 1.694040 |
| TCGA-EW-A2FS-01A-11R-A17B-07 | 1.352823 | 1.248907 | 1.171422 | 1.205154 | 1.420731 | 1.500784 | 1.443278 | 1.347984 | 1.438744 | 1.662352 |
| TCGA-EW-A1P7-01A-21R-A144-07 | 1.396801 | 1.311661 | 1.327573 | 1.349573 | 1.428941 | 1.588284 | 1.447075 | 1.425700 | 1.467098 | 1.670157 |
| TCGA-LL-A5YO-01A-21R-A28M-07 | 1.260467 | 1.279847 | 1.182847 | 1.218694 | 1.414804 | 1.546093 | 1.354670 | 1.407807 | 1.400502 | 1.674053 |
| TCGA-BH-A1FN-01A-11R-A13Q-07 | 1.343863 | 1.314693 | 1.287907 | 1.161825 | 1.474510 | 1.539289 | 1.512237 | 1.495506 | 1.476215 | 1.692673 |

In [478]:

```
head(nes.score.sub.plot)
```

A data.frame: 6 × 3

|  | Group | Pathway | NES |
| --- | --- | --- | --- |
|  | <dbl> | <fct> | <dbl> |
| 1 | 0.2203707 | RTK\_RAS | -0.4137421 |
| 2 | -1.1014146 | RTK\_RAS | -0.7883398 |
| 3 | -0.3787754 | RTK\_RAS | -0.1492681 |
| 4 | -0.8431213 | RTK\_RAS | 0.5710126 |
| 5 | 0.6170595 | RTK\_RAS | -1.6618908 |
| 6 | 1.7747969 | RTK\_RAS | -0.2960191 |

In [485]:

```
options(repr.plot.width = 15, repr.plot.height = 6)
names(cols) <- c('High', 'Low')

nes.score.sub.plot <- cbind.data.frame(nes.score.sub %>% scale, Group = as.vector(risk.socres))
nes.score.sub.plot <- tidyr::gather(nes.score.sub.plot, 'Pathway', 'NES',  -Group)
nes.score.sub.plot$Pathway <- factor(nes.score.sub.plot$Pathway, levels = colnames(nes.score.sub))

ggplot(nes.score.sub.plot, aes(x = Group, y = NES)) +  facet_wrap(~Pathway, ncol = 5) + geom_point(size = 3, alpha = 0.5, color = 'blue')+ geom_smooth(method = "lm", se = T, color = 'red', lwd = 1.5) + stat_cor(method="pearson") + theme_bw(base_size = 20) 
#stat_compare_means(label = "p.signif") + theme_classic(base_size = 20) + scale_fill_manual(values = cols) + ylim(-3, 3) + theme(axis.text.x = element_text(angle = 45, vjust = 1, hjust=1), legend.position="top") + xlab('')
ggsave(file.path(out.figs.dir, 'nes.high.vs.low.scatter.pdf'), height = 6, width = 10)
```

```
`geom_smooth()` using formula 'y ~ x'

`geom_smooth()` using formula 'y ~ x'
```

### DEGs analyssi¶

In [53]:

```
label <- ifelse(res.cox$group == 'High risk', 'High', 'Low')
names(label) <- colnames(tcga.obj.sub)
group.list <- factor(label)

design <- model.matrix(~0 + group.list)
colnames(design) <- levels(group.list)
rownames(design) <- colnames(tcga.obj.sub)
contrast.matrix <- makeContrasts('High-Low',levels = design)
fit.lm <- lmFit(GetAssayData(tcga.obj.sub) %>% as.matrix, design)
fit2 <- contrasts.fit(fit.lm, contrast.matrix)
fit2 <- eBayes(fit2)
DEgeneSets <- topTable(fit2, coef=1, number=Inf, p.value=1e-2, adjust="BH")
```

In [54]:

```
table(label)
```

```
label
High  Low 
 179  930
```

In [55]:

```
head(DEgeneSets)
```

A data.frame: 6 × 6

|  | logFC | AveExpr | t | P.Value | adj.P.Val | B |
| --- | --- | --- | --- | --- | --- | --- |
|  | <dbl> | <dbl> | <dbl> | <dbl> | <dbl> | <dbl> |
| LINC00346 | 0.9279150 | 1.004204 | 7.804087 | 1.380583e-14 | 7.533153e-10 | 21.93668 |
| MOB3C | -0.6611918 | 3.269817 | -6.808866 | 1.609559e-11 | 4.391279e-07 | 15.01021 |
| GIMAP2 | -2.0170385 | 5.254155 | -6.711314 | 3.072753e-11 | 5.588825e-07 | 14.37773 |
| UBA7 | -3.4827201 | 10.869262 | -6.597015 | 6.486447e-11 | 8.848325e-07 | 13.64737 |
| APOBEC3G | -1.2880137 | 2.731514 | -6.482253 | 1.357821e-10 | 1.481790e-06 | 12.92573 |
| CCDC97 | -2.2995720 | 14.719584 | -6.349482 | 3.146348e-10 | 2.861341e-06 | 12.10551 |

In [78]:

```
deg.up.high <- subset(DEgeneSets, logFC > 1)
dim(deg.up.high)
deg.up.high.genes <- deg.up.high[order(deg.up.high$logFC) %>% rev, ] %>% rownames(.)
deg.up.high.anno <- bitr(deg.up.high.genes, fromType = 'SYMBOL', toType = 'ENTREZID', OrgDb = 'org.Hs.eg.db', drop = T)
gene.lst <- deg.up.high[deg.up.high.anno$SYMBOL, 'logFC']
names(gene.lst) <- deg.up.high.anno[, 'ENTREZID']
gsea.high.up <-gseGO(geneList = gene.lst, nPerm = 1000, ont = 'BP', pvalueCutoff = 0.05, OrgDb = org.Hs.eg.db)
as.data.frame(gsea.high.up) %>% head
```

1. 186
2. 6

```
'select()' returned 1:1 mapping between keys and columns

preparing geneSet collections...

GSEA analysis...

leading edge analysis...

done...
```

A data.frame: 6 × 11

|  | ID | Description | setSize | enrichmentScore | NES | pvalue | p.adjust | qvalues | rank | leading\_edge | core\_enrichment |
| --- | --- | --- | --- | --- | --- | --- | --- | --- | --- | --- | --- |
|  | <chr> | <chr> | <int> | <dbl> | <dbl> | <dbl> | <dbl> | <dbl> | <int> | <chr> | <chr> |
| GO:0007275 | GO:0007275 | multicellular organism development | 56 | 0.7926693 | 1.373971 | 0.000999001 | 0.02284473 | 0.0202762 | 19 | tags=23%, list=11%, signal=31% | 6277/1382/3326/3320/6382/114569/999/6271/5230/4320/5885/10397/3304 |
| GO:0030154 | GO:0030154 | cell differentiation | 48 | 0.8328021 | 1.449795 | 0.000999001 | 0.02284473 | 0.0202762 | 19 | tags=27%, list=11%, signal=34% | 6277/1382/3326/3320/8407/6382/6281/999/5230/1672/4320/10397/3304 |
| GO:0032501 | GO:0032501 | multicellular organismal process | 69 | 0.7771564 | 1.338691 | 0.000999001 | 0.02284473 | 0.0202762 | 19 | tags=20%, list=11%, signal=31% | 6277/1382/3326/3320/6382/114569/999/6271/5230/1672/4320/5885/10397/3304 |
| GO:0048518 | GO:0048518 | positive regulation of biological process | 68 | 0.7627155 | 1.313571 | 0.000999001 | 0.02284473 | 0.0202762 | 30 | tags=29%, list=18%, signal=41% | 6277/1382/3326/3320/1917/6382/3312/6281/999/6271/1672/5885/3304/10276/794/7879/6836/10575/7532/3799 |
| GO:0048869 | GO:0048869 | cellular developmental process | 49 | 0.8296615 | 1.443184 | 0.000999001 | 0.02284473 | 0.0202762 | 19 | tags=27%, list=11%, signal=33% | 6277/1382/3326/3320/8407/6382/6281/999/5230/1672/4320/10397/3304 |
| GO:0009653 | GO:0009653 | anatomical structure morphogenesis | 33 | 0.8584428 | 1.501807 | 0.001000000 | 0.02284473 | 0.0202762 | 14 | tags=24%, list=8%, signal=28% | 6277/1382/3326/3320/6382/6281/6271/5230 |

In [94]:

```
deg.up.low <- subset(DEgeneSets, logFC < -1)
dim(deg.up.low)
deg.up.low.genes <- deg.up.low[order(deg.up.low$logFC) %>% rev, ] %>% rownames(.)
deg.up.low.anno <- bitr(deg.up.low.genes, fromType = 'SYMBOL', toType = 'ENTREZID', OrgDb = 'org.Hs.eg.db', drop = T)
gene.lst <- deg.up.low[deg.up.low.anno$SYMBOL, 'logFC']
names(gene.lst) <- deg.up.low.anno[, 'ENTREZID']
gsea.low.up <-gseGO(geneList = gene.lst, nPerm = 1000, ont = 'BP', pvalueCutoff = 0.05, OrgDb = org.Hs.eg.db)
as.data.frame(gsea.low.up) %>% head
```

1. 370
2. 6

```
'select()' returned 1:1 mapping between keys and columns

preparing geneSet collections...

GSEA analysis...

leading edge analysis...

done...
```

A data.frame: 6 × 11

|  | ID | Description | setSize | enrichmentScore | NES | pvalue | p.adjust | qvalues | rank | leading\_edge | core\_enrichment |
| --- | --- | --- | --- | --- | --- | --- | --- | --- | --- | --- | --- |
|  | <chr> | <chr> | <int> | <dbl> | <dbl> | <dbl> | <dbl> | <dbl> | <dbl> | <chr> | <chr> |
| GO:0001775 | GO:0001775 | cell activation | 100 | -0.8091069 | -1.390921 | 0.000999001 | 0.02784266 | 0.02150298 | 26 | tags=15%, list=7%, signal=19% | 7305/3936/677/7805/678/6352/3113/6363/3115/3133/3123/567/3122/972/3493 |
| GO:0002250 | GO:0002250 | adaptive immune response | 70 | -0.8438824 | -1.484318 | 0.000999001 | 0.02784266 | 0.02150298 | 18 | tags=14%, list=5%, signal=17% | 3113/6363/3115/3127/3133/3123/567/3122/972/3493 |
| GO:0002443 | GO:0002443 | leukocyte mediated immunity | 57 | -0.8776227 | -1.557557 | 0.000999001 | 0.02784266 | 0.02150298 | 7 | tags=11%, list=2%, signal=12% | 3133/3123/567/3122/972/3493 |
| GO:0002684 | GO:0002684 | positive regulation of immune system process | 85 | -0.8233067 | -1.431402 | 0.000999001 | 0.02784266 | 0.02150298 | 39 | tags=25%, list=11%, signal=29% | 28755/5721/3119/5696/3118/4239/7305/677/7805/6352/3113/6363/5720/3115/3127/3133/3123/567/3122/972/3493 |
| GO:0002694 | GO:0002694 | regulation of leukocyte activation | 58 | -0.8656032 | -1.532774 | 0.000999001 | 0.02784266 | 0.02150298 | 26 | tags=22%, list=7%, signal=25% | 7305/677/7805/678/6352/3113/6363/3115/3133/3123/3122/972/3493 |
| GO:0006950 | GO:0006950 | response to stress | 146 | -0.8061566 | -1.371842 | 0.000999001 | 0.02784266 | 0.02150298 | 38 | tags=21%, list=11%, signal=33% | 5721/3119/1153/5696/4792/3118/6890/3178/9246/358/4239/7305/677/7805/27250/678/6352/3113/6772/6363/4283/5720/3115/7832/3127/3133/3123/567/3122/972/3493 |

In [112]:

```
gsea.high.up.sorted <- gsea.high.up[order(gsea.high.up$enrichmentScore,decreasing=T)]
gsea.low.up.sorted <- gsea.low.up[order(gsea.low.up$enrichmentScore,decreasing = T)]
```

In [115]:

```
row.names(gsea.high.up.sorted)[1 : 5]
```

1. 'GO:0048812'
2. 'GO:0048858'
3. 'GO:0120039'
4. 'GO:0061564'
5. 'GO:0032989'

In [146]:

```
options(repr.plot.width = 10, repr.plot.height = 6)
gsea.1 <- gseaplot2(gsea.high.up, row.names(gsea.high.up %>% as.data.frame)[1 : 5], base_size = 20)
gsea.1
```

In [147]:

```
gsea.2 <- gseaplot2(gsea.low.up, row.names(gsea.low.up %>% as.data.frame)[1:5], base_size = 20) 
gsea.2
```

In [150]:

```
options(repr.plot.width = 20, repr.plot.height = 6)
ggarrange(gsea.1, gsea.2, nrow = 1)
ggsave(file.path(out.figs.dir, 'gsea.anno.pdf'), width = 18, height = 7)
```

### Mutation analysis¶

In [ ]:

```
library(maftools)
library(TCGAbiolinks)
BRACmut <- GDCquery_Maf(tumor = "BRCA", pipelines = "mutect2")
```

In [164]:

```
mut.barcode <- substr(BRACmut$Tumor_Sample_Barcode, 1, 12)
idx.high <- which(mut.barcode %in% (subset(tcga.obj.sub, Group == 'High risk') %>% Cells %>% substr(., 1, 12)))
idx.low <- which(mut.barcode %in% (subset(tcga.obj.sub, Group == 'Low risk') %>% Cells %>% substr(., 1, 12)))
primary.brac <- read.maf(maf = BRACmut[idx.high, ])
relapse.brac <- read.maf(maf = BRACmut[idx.low, ])
```

```
-Validating
-Silent variants: 9552 
-Summarizing
--Possible FLAGS among top ten genes:
  TTN
  MUC16
  FLG
-Processing clinical data
--Missing clinical data
-Finished in 3.380s elapsed (1.540s cpu) 
-Validating
-Silent variants: 35495 
-Summarizing
--Possible FLAGS among top ten genes:
  TTN
  MUC16
  HMCN1
  USH2A
-Processing clinical data
--Missing clinical data
-Finished in 9.970s elapsed (9.630s cpu)
```

In [234]:

```
options(repr.plot.width = 20, repr.plot.height = 10)
cols <- unlist(PAIRWISE_COLORS)
names(cols) <- c('High risk', 'Low risk')
pdf(file.path(out.figs.dir, 'Mutation_high_vs_low.pdf'), height = 10, width = 8)
pt.vs.rt <- mafCompare(m1 = primary.brac, m2 = relapse.brac, m1Name = 'High risk', m2Name = 'Low risk', minMut = 10)
forestPlot(mafCompareRes = pt.vs.rt, pVal = 0.05, titleSize = 1.8, geneFontSize = 1.5)
dev.off()
pt.vs.rt$results %>% head
forestPlot(mafCompareRes = pt.vs.rt, pVal = 0.05, titleSize = 1.8, geneFontSize = 1.5)
```

**png:** 2

A data.table: 6 × 8

| Hugo\_Symbol | High risk | Low risk | pval | or | ci.up | ci.low | adjPval |
| --- | --- | --- | --- | --- | --- | --- | --- |
| <chr> | <dbl> | <int> | <dbl> | <dbl> | <dbl> | <dbl> | <dbl> |
| ERBB3 | 11 | 8 | 3.866713e-05 | 7.4193866 | 21.6264536 | 2.6677619 | 0.03248039 |
| FMN2 | 12 | 15 | 4.708113e-04 | 4.3128773 | 10.0960972 | 1.8041102 | 0.19774075 |
| CDH1 | 10 | 128 | 1.144698e-03 | 0.3583434 | 0.7022034 | 0.1638182 | 0.32051555 |
| DNAH10 | 10 | 15 | 3.748031e-03 | 3.5476364 | 8.6263010 | 1.3972208 | 0.78708654 |
| MIA3 | 8 | 11 | 6.625547e-03 | 3.8379871 | 10.6766250 | 1.3171137 | 0.99038359 |
| FOXA1 | 9 | 14 | 7.074169e-03 | 3.4028960 | 8.6150640 | 1.2749707 | 0.99038359 |

In [368]:

```
tab.plot <- subset(pt.vs.rt$results, pval < 0.01)[,1 : 7 ] %>% t

tab.plot[4, ] <- round(as.numeric(tab.plot[4, ]), 3)
tab.plot[5, ] <- round(as.numeric(tab.plot[5, ]), 3)
tab.plot[6, ] <- round(as.numeric(tab.plot[6, ]), 3)
tab.plot[7, ] <- round(as.numeric(tab.plot[7, ]), 3)
head(tab.plot)
```

A matrix: 6 × 6 of type chr

|  |  |  |  |  |  |  |
| --- | --- | --- | --- | --- | --- | --- |
| Hugo\_Symbol | ERBB3 | FMN2 | CDH1 | DNAH10 | MIA3 | FOXA1 |
| High risk | 11 | 12 | 10 | 10 | 8 | 9 |
| Low risk | 8 | 15 | 128 | 15 | 11 | 14 |
| pval | 0 | 0 | 0.001 | 0.004 | 0.007 | 0.007 |
| or | 7.419 | 4.313 | 0.358 | 3.548 | 3.838 | 3.403 |
| ci.up | 21.626 | 10.096 | 0.702 | 8.626 | 10.677 | 8.615 |

In [369]:

```
tab.plot <- t(tab.plot)
colnames(tab.plot) <- c('Hugo_Symbol', 'High risk\n(count)', 'Low risk\n(count)', 'P-value', 'OR', 'CI.up', 'CI.low')
```

In [370]:

```
tab.plot <- tab.plot[, c('Hugo_Symbol', 'High risk\n(count)', 'Low risk\n(count)', 'OR', 'CI.up', 'CI.low', 'P-value')]
```

In [371]:

```
grid.table(tab.plot)
```

In [372]:

```
pdf(file.path(out.figs.dir, 'sig.mut.genes.pdf'), width = 6, height = 10)
grid.table(tab.plot)
dev.off()
```

**png:** 2

In [320]:

```
options(repr.plot.width = 10, repr.plot.height = 10)
pdf(file.path(out.figs.dir, 'oncoplot.high.risk.pdf'), height = 8, width = 8)
oncoplot(maf = primary.brac, top = 10, fontSize = 1.0, legendFontSize = 1.5, annotationFontSize = 1.5)
dev.off()
oncoplot(maf = primary.brac, top = 10, fontSize = 1.2)

pdf(file.path(out.figs.dir, 'oncoplot.low.risk.pdf'), height = 8, width = 8)
oncoplot(maf = relapse.brac, top = 10, fontSize = 1.0, legendFontSize = 1.5, annotationFontSize = 1.5)
dev.off()
oncoplot(maf = relapse.brac, top = 10, fontSize = 1.2)
```

**png:** 2

**png:** 2

In [237]:

```
maf.brac <- read.maf(maf = BRACmut)
tmb.bc <- tmb(maf.brac, captureSize = 50, logScale = TRUE)
```

```
-Validating
-Silent variants: 45177 
-Summarizing
--Possible FLAGS among top ten genes:
  TTN
  MUC16
  HMCN1
-Processing clinical data
--Missing clinical data
-Finished in 15.4s elapsed (13.6s cpu)
```

In [252]:

```
tmb.bc %>% head
```

A data.table: 6 × 4

| Tumor\_Sample\_Barcode | total | total\_perMB | total\_perMB\_log |
| --- | --- | --- | --- |
| <fct> | <dbl> | <dbl> | <dbl> |
| TCGA-AC-A2FK-01A-12D-A17W-09 | 0 | 0.00 | -Inf |
| TCGA-A8-A08C-01A-11W-A019-09 | 1 | 0.02 | -1.69897 |
| TCGA-AO-A1KO-01A-31D-A188-09 | 4 | 0.08 | -1.09691 |
| TCGA-PL-A8LY-01A-11D-A41F-09 | 4 | 0.08 | -1.09691 |
| TCGA-A2-A25F-01A-11D-A167-09 | 5 | 0.10 | -1.00000 |
| TCGA-LL-A440-01A-11D-A243-09 | 5 | 0.10 | -1.00000 |

In [284]:

```
risk.socres.sub <- risk.socres[, !duplicated(substr(colnames(risk.socres), 1, 12))]
length(risk.socres.sub)
```

1091

In [287]:

```
tmb.bc.sub <- tmb.bc[!duplicated(substr(tmb.bc$Tumor_Sample_Barcode, 1, 12)), ] %>% as.data.frame
dim(tmb.bc.sub)
rownames(tmb.bc.sub) <- substr(tmb.bc.sub$Tumor_Sample_Barcode, 1, 12)
```

1. 986
2. 4

In [288]:

```
head(tmb.bc.sub)
```

A data.frame: 6 × 4

|  | Tumor\_Sample\_Barcode | total | total\_perMB | total\_perMB\_log |
| --- | --- | --- | --- | --- |
|  | <fct> | <dbl> | <dbl> | <dbl> |
| TCGA-AC-A2FK | TCGA-AC-A2FK-01A-12D-A17W-09 | 0 | 0.00 | -Inf |
| TCGA-A8-A08C | TCGA-A8-A08C-01A-11W-A019-09 | 1 | 0.02 | -1.69897 |
| TCGA-AO-A1KO | TCGA-AO-A1KO-01A-31D-A188-09 | 4 | 0.08 | -1.09691 |
| TCGA-PL-A8LY | TCGA-PL-A8LY-01A-11D-A41F-09 | 4 | 0.08 | -1.09691 |
| TCGA-A2-A25F | TCGA-A2-A25F-01A-11D-A167-09 | 5 | 0.10 | -1.00000 |
| TCGA-LL-A440 | TCGA-LL-A440-01A-11D-A243-09 | 5 | 0.10 | -1.00000 |

In [289]:

```
names(risk.socres.sub) <- substr(names(risk.socres.sub), 1, 12)
```

In [290]:

```
tmb.bc.sub %>% dim
```

1. 986
2. 4

In [291]:

```
comm.SN <- intersect(names(risk.socres.sub), rownames(tmb.bc.sub))
risk.socres.sub <- risk.socres.sub[comm.SN]
tmb.bc.sub <- data.frame(tmb.bc.sub)[comm.SN, ]
head(tmb.bc.sub)
```

A data.frame: 6 × 4

|  | Tumor\_Sample\_Barcode | total | total\_perMB | total\_perMB\_log |
| --- | --- | --- | --- | --- |
|  | <fct> | <dbl> | <dbl> | <dbl> |
| TCGA-E2-A15G | TCGA-E2-A15G-01A-11D-A12B-09 | 50 | 1.00 | 0.0000000 |
| TCGA-E2-A1B5 | TCGA-E2-A1B5-01A-21D-A12Q-09 | 27 | 0.54 | -0.2676062 |
| TCGA-EW-A2FS | TCGA-EW-A2FS-01A-11D-A17D-09 | 33 | 0.66 | -0.1804561 |
| TCGA-EW-A1P7 | TCGA-EW-A1P7-01A-21D-A142-09 | 19 | 0.38 | -0.4202164 |
| TCGA-LL-A5YO | TCGA-LL-A5YO-01A-21D-A28B-09 | 39 | 0.78 | -0.1079054 |
| TCGA-BH-A1FN | TCGA-BH-A1FN-01A-11D-A13L-09 | 92 | 1.84 | 0.2648178 |

In [322]:

```
plot.df.tmp <- cbind.data.frame(tmb.bc.sub, Score = ifelse(risk.socres.sub > res.cox$cutoff, 'High', 'Low'))
names(cols) <- c('High', 'Low')
ggplot(plot.df.tmp, aes(x = Score, y = total_perMB_log, fill = Score)) + geom_boxplot() + theme_classic(base_size = 20) + stat_compare_means(label = "p.format") + scale_fill_manual(values = cols)
ggsave(file.path(out.figs.dir, 'tmp.high.vs.low.pdf'), height = 6, width = 6)
```

In [330]:

```
head(plot.df.tmp)
```

A data.frame: 6 × 5

|  | Tumor\_Sample\_Barcode | total | total\_perMB | total\_perMB\_log | Score |
| --- | --- | --- | --- | --- | --- |
|  | <fct> | <dbl> | <dbl> | <dbl> | <dbl> |
| TCGA-E2-A15G | TCGA-E2-A15G-01A-11D-A12B-09 | 50 | 1.00 | 0.0000000 | 0.2203707 |
| TCGA-E2-A1B5 | TCGA-E2-A1B5-01A-21D-A12Q-09 | 27 | 0.54 | -0.2676062 | -1.1014146 |
| TCGA-EW-A2FS | TCGA-EW-A2FS-01A-11D-A17D-09 | 33 | 0.66 | -0.1804561 | -0.3787754 |
| TCGA-EW-A1P7 | TCGA-EW-A1P7-01A-21D-A142-09 | 19 | 0.38 | -0.4202164 | -0.8431213 |
| TCGA-LL-A5YO | TCGA-LL-A5YO-01A-21D-A28B-09 | 39 | 0.78 | -0.1079054 | 0.6170595 |
| TCGA-BH-A1FN | TCGA-BH-A1FN-01A-11D-A13L-09 | 92 | 1.84 | 0.2648178 | 1.7747969 |

In [333]:

```
plot.df.tmp <- cbind.data.frame(tmb.bc.sub, Score = risk.socres.sub)
names(cols) <- c('High', 'Low')
ggplot(plot.df.tmp, aes(x = Score, y = total_perMB_log, fill = Score)) + geom_point() + geom_smooth(method = "lm", se = T, color = 'red', lwd = 1.5) + stat_cor(method="pearson") + theme_classic(base_size = 20) + theme(legend.position = 'none')
#ggsave(file.path(out.figs.dir, 'tmp.high.vs.low.pdf'), height = 6, width = 6)
```

```
`geom_smooth()` using formula 'y ~ x'
```

### Correlation with markers of Fiblasts cell¶

In [132]:

```
tmp.xls <- file.path(out.data.dir, 'tmp_training_data.xls')
tmp.gct <- file.path(out.data.dir, 'tmp_training_data.gct')
tmp.score <- file.path(out.data.dir, 'tmp_immune_scores.gct')

write.table(GetAssayData(tcga.obj.sub) %>% as.matrix %>% { log2(1 + .)}, tmp.xls, sep = '\t', row.names = TRUE, col.names = NA, quote = FALSE)
```

In [133]:

```
library(estimate)
filterCommonGenes(tmp.xls, output.f = tmp.gct, id = "GeneSymbol")
estimateScore(tmp.gct, tmp.score, platform  = 'affymetrix')
purity.score <- read.table(tmp.score, sep = '\t', header = TRUE, row.names = 1, skip = 2) %>% .[, -1]
```

```
[1] "Merged dataset includes 10214 genes (198 mismatched)."
[1] "1 gene set: StromalSignature  overlap= 139"
[1] "2 gene set: ImmuneSignature  overlap= 141"
```

In [135]:

```
purity.score.t <- purity.score %>% t
head(purity.score.t)
```

A matrix: 6 × 4 of type dbl

|  | StromalScore | ImmuneScore | ESTIMATEScore | TumorPurity |
| --- | --- | --- | --- | --- |
| TCGA.E2.A15G.01A.11R.A12D.07 | -194.46335 | -89.87435 | -284.3377 | 0.8455244 |
| TCGA.E2.A1B5.01A.21R.A12P.07 | 829.19480 | 2673.47073 | 3502.6655 | 0.4364583 |
| TCGA.EW.A2FS.01A.11R.A17B.07 | 89.66423 | 806.83986 | 896.5041 | 0.7407679 |
| TCGA.EW.A1P7.01A.21R.A144.07 | 1154.16065 | 2669.38048 | 3823.5411 | 0.3936121 |
| TCGA.LL.A5YO.01A.21R.A28M.07 | 712.35812 | 2842.81553 | 3555.1736 | 0.4295107 |
| TCGA.BH.A1FN.01A.11R.A13Q.07 | 348.58867 | 75.83553 | 424.4242 | 0.7855030 |

In [136]:

```
fib.genes <- list('Fibroblasts' = c('COL1A1','COL3A1','COL6A1','COL6A2','DCN','GREM1','PAMR1','TAGLN'))
end.genes <- c('ACVRL1','APLN','BCL6B','BMP6','BMX','CDH5','CLEC14A','CXorf36','EDN1','ELTD1','EMCN','ESAM','ESM1','HECW2','HHIP','KDR','MMRN1','MMRN2','MYCT1','PALMD','PEAR1','PGF','PLXNA2','PTPRB','ROBO4','SDPR','SHANK3','SHE','TEK','TIE1','VEPH1','VWF')
```

In [146]:

```
head(plot.data.fib)
```

A data.frame: 6 × 3

|  | Fibroblasts | Risk | Group |
| --- | --- | --- | --- |
|  | <dbl> | <dbl> | <chr> |
| 1 | -89.87435 | 0.2203707 | Low risk |
| 2 | 2673.47073 | -1.1014146 | Low risk |
| 3 | 806.83986 | -0.3787754 | Low risk |
| 4 | 2669.38048 | -0.8431213 | Low risk |
| 5 | 2842.81553 | 0.6170595 | Low risk |
| 6 | 75.83553 | 1.7747969 | High risk |

In [170]:

```
options(repr.plot.width = 10, repr.plot.height = 6)
cols <- unlist(PAIRWISE_COLORS)
names(cols) <- c('High risk', 'Low risk')
plot.data.fib <- cbind.data.frame(Stromal =  as.vector(purity.score.t[, 1]), Risk = as.vector(risk.socres), Group = res.cox$group)
ggplot(plot.data.fib, aes(x = scale(Risk), y = scale(Stromal))) + geom_point(size = 3, alpha = 0.5, color = 'blue')+ geom_smooth(method = "lm", se = T, color = 'red', lwd = 1.5) + stat_cor(method="pearson") + theme_classic(base_size = 20) + theme(legend.position = 'none')
gp.1 <- ggplot(plot.data.fib, aes(x = Group, y = scale(Stromal), fill = Group)) + geom_boxplot() + theme_classic(base_size = 20) + stat_compare_means(label = "p.format") + scale_fill_manual(values = cols)
gp.1
```

```
`geom_smooth()` using formula 'y ~ x'
```

In [171]:

```
plot.data.fib <- cbind.data.frame(Immune =  as.vector(purity.score.t[, 2]), Risk = as.vector(risk.socres), Group = res.cox$group)
ggplot(plot.data.fib, aes(x = scale(Risk), y = scale(Immune))) + geom_point(size = 3, alpha = 0.5, color = 'blue')+ geom_smooth(method = "lm", se = T, color = 'red', lwd = 1.5) + stat_cor(method="pearson") + theme_classic(base_size = 20) + theme(legend.position = 'none')
gp.2 <- ggplot(plot.data.fib, aes(x = Group, y = scale(Immune), fill = Group)) + geom_boxplot() + theme_classic(base_size = 20) + stat_compare_means(label = "p.format") + scale_fill_manual(values = cols)
gp.2
```

```
`geom_smooth()` using formula 'y ~ x'
```

In [172]:

```
plot.data.fib <- cbind.data.frame(Tumor =  as.vector(purity.score.t[, 4]), Risk = as.vector(risk.socres), Group = res.cox$group)
ggplot(plot.data.fib, aes(x = scale(Risk), y = scale(Tumor))) + geom_point(size = 3, alpha = 0.5, color = 'blue')+ geom_smooth(method = "lm", se = T, color = 'red', lwd = 1.5) + stat_cor(method="pearson") + theme_classic(base_size = 20) + theme(legend.position = 'none')
gp.3 <- ggplot(plot.data.fib, aes(x = Group, y = scale(Tumor), fill = Group)) + geom_boxplot() + theme_classic(base_size = 20) + stat_compare_means(label = "p.format") + scale_fill_manual(values = cols)
gp.3
```

```
`geom_smooth()` using formula 'y ~ x'
```

In [176]:

```
options(repr.plot.width = 15, repr.plot.height = 6)
gp.1 + gp.2 + gp.3
ggsave(file.path(out.figs.dir, 'estimate.brca.pdf'), width = 15, height = 6)
```

### lincRNA-mRNA networrk¶

In [59]:

```
anno <- read.table('../0.data/gencode.gene.info.v22.tsv', sep = '\t', header = T) %>% subset(., gene_type == 'protein_coding')
head(anno)
com.genes <- intersect(rownames(tcga.obj.sub),anno$gene_name)
length(com.genes)
#tcga.obj.sub.sub <- subset(tcga.obj.sub, features = com.genes)
```

A data.frame: 6 × 12

|  | gene\_id | gene\_name | seqname | start | end | strand | gene\_type | gene\_status | havana\_gene | full\_length | exon\_length | exon\_num |
| --- | --- | --- | --- | --- | --- | --- | --- | --- | --- | --- | --- | --- |
|  | <chr> | <chr> | <chr> | <int> | <int> | <chr> | <chr> | <chr> | <chr> | <int> | <int> | <int> |
| 7 | ENSG00000206557.5 | TRIM71 | chr3 | 32818018 | 32897826 | + | protein\_coding | KNOWN | OTTHUMG00000155778.3 | 79809 | 8685 | 4 |
| 8 | ENSG00000183813.6 | CCR4 | chr3 | 32951574 | 32956349 | + | protein\_coding | KNOWN | OTTHUMG00000130752.2 | 4776 | 3095 | 2 |
| 9 | ENSG00000170266.14 | GLB1 | chr3 | 32996608 | 33097230 | - | protein\_coding | KNOWN | OTTHUMG00000155781.6 | 100623 | 4364 | 123 |
| 12 | ENSG00000188167.7 | TMPPE | chr3 | 33090421 | 33096801 | - | protein\_coding | KNOWN | OTTHUMG00000155779.1 | 6381 | 3966 | 4 |
| 14 | ENSG00000170275.13 | CRTAP | chr3 | 33113979 | 33147773 | + | protein\_coding | KNOWN | OTTHUMG00000130746.3 | 33795 | 6695 | 18 |
| 17 | ENSG00000173705.7 | SUSD5 | chr3 | 33150042 | 33219215 | - | protein\_coding | KNOWN | OTTHUMG00000155829.2 | 69174 | 5008 | 9 |

19459

In [60]:

```
expr <- GetAssayData(tcga.obj.sub) %>% as.matrix %>% .[com.genes, ]
```

In [61]:

```
linc.mrna <- lapply(gsub('_', '-', hub.genes.sub), function(gene) {
    lnc.expr <- FetchData(tcga.obj.sub, vars = gene)
    cor.res <- cor(lnc.expr, expr %>% t)
    cor.res <- cor.res[1, ][order(cor.res[1, ]) %>% rev]
    cor.res <- cor.res[!is.na(cor.res)] 
    cor.res[1:50] %>% names
})
```

In [62]:

```
names(linc.mrna) <- hub.genes.sub
```

In [63]:

```
genes.map <- clusterProfiler::bitr(unlist(linc.mrna, use.names = F) %>% unique, fromType = "SYMBOL", toType = c("ENTREZID", "SYMBOL"), OrgDb = org.Hs.eg.db)
ego <- clusterProfiler::enrichGO(
    gene          = genes.map$ENTREZID %>% unique,
    OrgDb         = org.Hs.eg.db,
    ont           = "BP",
    pAdjustMethod = "BH",
    pvalueCutoff  = 0.01,
    qvalueCutoff  = 0.01,
    readable      = TRUE
)
```

```
'select()' returned 1:many mapping between keys and columns
```

In [64]:

```
data.frame(ego)
```

A data.frame: 10 × 9

|  | ID | Description | GeneRatio | BgRatio | pvalue | p.adjust | qvalue | geneID | Count |
| --- | --- | --- | --- | --- | --- | --- | --- | --- | --- |
|  | <chr> | <chr> | <chr> | <chr> | <dbl> | <dbl> | <dbl> | <chr> | <int> |
| GO:0042110 | GO:0042110 | T cell activation | 58/1038 | 474/18862 | 9.283775e-09 | 4.515628e-05 | 4.408327e-05 | GNRH1/VNN1/TGFBR2/DPP4/PAK3/NCK2/FZD7/MR1/KIF13B/RORC/PELI1/THY1/HHLA2/PLA2G2F/LIG4/IFNK/IFNE/PIK3R1/IFNA1/PTPN2/CD70/CCND3/TBX21/PDCD1/CD7/CD3D/CCL5/SLA2/CD2/CD8A/IFNG/SIRPG/IL12RB1/HLA-DRA/HLA-DPB1/LCK/ZAP70/CD74/IRF1/CD3E/TIGIT/CEBPB/LYN/LAG3/LGALS1/NLRC3/KLRK1/DOCK2/LAT/JAK3/CARD11/ITK/EOMES/CTLA4/RASAL3/WDFY4/PTPRC/GRAP2 | 58 |
| GO:0050863 | GO:0050863 | regulation of T cell activation | 40/1038 | 327/18862 | 1.876032e-06 | 4.147984e-03 | 4.049419e-03 | GNRH1/VNN1/TGFBR2/DPP4/PAK3/NCK2/PELI1/THY1/HHLA2/PLA2G2F/PIK3R1/PTPN2/CD70/TBX21/PDCD1/CCL5/CD2/IFNG/SIRPG/IL12RB1/HLA-DRA/HLA-DPB1/LCK/ZAP70/CD74/IRF1/CD3E/TIGIT/CEBPB/LYN/LAG3/LGALS1/KLRK1/LAT/JAK3/CARD11/CTLA4/RASAL3/PTPRC/GRAP2 | 40 |
| GO:0050852 | GO:0050852 | T cell receptor signaling pathway | 29/1038 | 204/18862 | 2.558378e-06 | 4.147984e-03 | 4.049419e-03 | BTN1A1/TXK/PAK3/KCNN4/PVRIG/THY1/HHLA2/PIK3R1/PTPN2/CD3D/SLA2/HLA-DRA/HLA-DPB1/CD247/PSMB9/LCK/ZAP70/UBASH3A/CD3E/GBP1/PSMB8/PSME2/LAT/CARD11/ITK/CTLA4/LCP2/PTPRC/GRAP2 | 29 |
| GO:0045059 | GO:0045059 | positive thymic T cell selection | 7/1038 | 14/18862 | 3.654224e-06 | 4.443537e-03 | 4.337949e-03 | PTPN2/CD3D/ZAP70/CD74/CD3E/DOCK2/PTPRC | 7 |
| GO:0050870 | GO:0050870 | positive regulation of T cell activation | 29/1038 | 212/18862 | 5.556663e-06 | 4.703347e-03 | 4.591585e-03 | VNN1/TGFBR2/DPP4/PAK3/NCK2/THY1/HHLA2/PIK3R1/CD70/PDCD1/CCL5/IFNG/SIRPG/IL12RB1/HLA-DRA/HLA-DPB1/LCK/ZAP70/CD74/CD3E/LYN/LGALS1/KLRK1/JAK3/CARD11/CTLA4/RASAL3/PTPRC/GRAP2 | 29 |
| GO:1903037 | GO:1903037 | regulation of leukocyte cell-cell adhesion | 39/1038 | 330/18862 | 5.801826e-06 | 4.703347e-03 | 4.591585e-03 | GNRH1/VNN1/TGFBR2/DPP4/PAK3/NCK2/PELI1/THY1/HHLA2/PLA2G2F/PPARA/PIK3R1/PTPN2/CD70/TBX21/PDCD1/CCL5/IFNG/SIRPG/IL12RB1/HLA-DRA/HLA-DPB1/LCK/ZAP70/CD74/IRF1/CD3E/TIGIT/CEBPB/LYN/LAG3/LGALS1/KLRK1/JAK3/CARD11/CTLA4/RASAL3/PTPRC/GRAP2 | 39 |
| GO:0031295 | GO:0031295 | T cell costimulation | 13/1038 | 57/18862 | 1.002652e-05 | 6.967002e-03 | 6.801452e-03 | DPP4/PAK3/HHLA2/PIK3R1/PDCD1/LCK/CD3E/LYN/LGALS1/KLRK1/CARD11/CTLA4/GRAP2 | 13 |
| GO:0045061 | GO:0045061 | thymic T cell selection | 8/1038 | 22/18862 | 1.309751e-05 | 7.277702e-03 | 7.104768e-03 | PTPN2/CD3D/ZAP70/CD74/CD3E/DOCK2/CARD11/PTPRC | 8 |
| GO:0071346 | GO:0071346 | cellular response to interferon-gamma | 25/1038 | 177/18862 | 1.381567e-05 | 7.277702e-03 | 7.104768e-03 | TLR2/TXK/VIM/RAB7B/MID1/DAPK1/PTPN2/FASLG/CCL5/IFNG/IL12RB1/HLA-DRA/HLA-DPB1/CCL4/XCL2/IRF1/HLA-F/GBP1/NMI/PML/GBP5/GBP4/GBP2/STAT1/CIITA | 25 |
| GO:0031294 | GO:0031294 | lymphocyte costimulation | 13/1038 | 59/18862 | 1.496238e-05 | 7.277702e-03 | 7.104768e-03 | DPP4/PAK3/HHLA2/PIK3R1/PDCD1/LCK/CD3E/LYN/LGALS1/KLRK1/CARD11/CTLA4/GRAP2 | 13 |

In [65]:

```
edox <- setReadable(ego, 'org.Hs.eg.db', 'ENTREZID')
```

In [66]:

```
options(repr.plot.width = 6, repr.plot.height = 6)
dotplot(ego, showCategory=30) + scale_color_viridis_c(guide=guide_colorbar(reverse=TRUE))
ggsave(file.path(out.figs.dir, 'go.anno.linc.pdf'), width = 6, height = 6)
```

```
Scale for 'colour' is already present. Adding another scale for 'colour',
which will replace the existing scale.
```

In [69]:

```
options(repr.plot.width = 12, repr.plot.height = 12)
cnetplot(edox, categorySize="pvalue", colorEdge = TRUE)
```

In [71]:

```
pdf('cnetplot.pdf', width = 12, height = 10)
cnetplot(edox, categorySize="pvalue", colorEdge = TRUE) 
dev.off()
```

**png:** 2

In [107]:

```
gene.lst <- lapply(split(data.frame(ego)$geneID, data.frame(ego)$Description), function(x) strsplit(x, '/')[[1]])
```

In [108]:

```
corre.score <- gsva(tcga.obj.sub %>% GetAssayData(.) %>% as.matrix, gene.lst, method = 'ssgsea')
```

```
Estimating ssGSEA scores for 10 gene sets.
  |======================================================================| 100%
```

In [109]:

```
linc.go.corr <- lapply(gsub('_', '-', hub.genes.sub), function(gene) {
    lnc.expr <- FetchData(tcga.obj.sub, vars = gene)
    cor.res <- cor(lnc.expr, corre.score %>% t)
}) %>% do.call(rbind, .)
```

In [111]:

```
(linc.go.corr)
```

A matrix: 29 × 10 of type dbl

|  | cellular response to interferon-gamma | lymphocyte costimulation | positive regulation of T cell activation | positive thymic T cell selection | regulation of leukocyte cell-cell adhesion | regulation of T cell activation | T cell activation | T cell costimulation | T cell receptor signaling pathway | thymic T cell selection |
| --- | --- | --- | --- | --- | --- | --- | --- | --- | --- | --- |
| AF131215.8 | -0.002570809 | 0.024897976 | 0.006964023 | 0.0025995741 | 0.0125568170 | 0.009061531 | 0.012340251 | 0.024897976 | 0.014739849 | 0.001126272 |
| LINC01235 | 0.122673282 | 0.131967622 | 0.113081846 | 0.0772729436 | 0.1239629500 | 0.114340862 | 0.108891493 | 0.131967622 | 0.123473749 | 0.077599911 |
| LINC00987 | 0.051208062 | 0.115890938 | 0.086429971 | 0.0595631392 | 0.0887826182 | 0.080536922 | 0.069113765 | 0.115890938 | 0.092314571 | 0.049293694 |
| LINC00398 | -0.151207151 | -0.126840292 | -0.127825425 | -0.0994882024 | -0.1410139884 | -0.137339985 | -0.100865428 | -0.126840292 | -0.127030757 | -0.088494218 |
| LINC00667 | -0.078316964 | -0.062622471 | -0.073060132 | -0.0325427459 | -0.0719594882 | -0.071304216 | -0.066952015 | -0.062622471 | -0.057365407 | -0.037208392 |
| CTD-2554C21.2 | -0.011531288 | -0.026592150 | -0.038015701 | -0.0235350483 | -0.0295173397 | -0.036019144 | -0.043664004 | -0.026592150 | -0.033745607 | -0.030705129 |
| ZNF888 | -0.077502463 | -0.069116864 | -0.084409219 | -0.0354857991 | -0.0990545396 | -0.098586487 | -0.075812294 | -0.069116864 | -0.074105056 | -0.041036636 |
| LL0XNC01-237H1.2 | -0.249311191 | -0.259236805 | -0.284164866 | -0.2128361676 | -0.2914067476 | -0.295757926 | -0.273941979 | -0.259236805 | -0.279913664 | -0.229807252 |
| AC016995.3 | 0.136823120 | 0.117129634 | 0.093054902 | 0.0602250314 | 0.1168784901 | 0.104184441 | 0.082080594 | 0.117129634 | 0.107351333 | 0.050228081 |
| SFTA1P | 0.019319121 | -0.010157858 | 0.018844278 | -0.0162401624 | 0.0180080835 | 0.015026166 | -0.005040032 | -0.010157858 | -0.003319005 | -0.014693806 |
| AL022344.7 | 0.095989007 | 0.082231248 | 0.078116042 | 0.0493280034 | 0.0919576458 | 0.084322799 | 0.071290150 | 0.082231248 | 0.063197602 | 0.042290879 |
| LINC00958 | 0.001371099 | 0.012051565 | -0.009860696 | -0.0302221835 | -0.0009752959 | -0.006075734 | -0.012744752 | 0.012051565 | -0.007917133 | -0.032914727 |
| LINC00327 | -0.023812613 | 0.001885152 | -0.015380563 | -0.0442239958 | -0.0189942220 | -0.027717485 | -0.031022985 | 0.001885152 | -0.023424900 | -0.046706195 |
| LINC00346 | 0.049899773 | -0.005328880 | 0.012020581 | -0.0055536841 | 0.0251391258 | 0.019519200 | 0.004491049 | -0.005328880 | 0.004758881 | -0.006779333 |
| AC016735.1 | 0.062060509 | 0.048205764 | 0.032065069 | 0.0028561901 | 0.0518629846 | 0.042901662 | 0.022654084 | 0.048205764 | 0.035424832 | -0.006152402 |
| AC009495.2 | 0.222869594 | 0.177649874 | 0.160996503 | 0.1452074846 | 0.1864808621 | 0.174554106 | 0.156174786 | 0.177649874 | 0.171451605 | 0.128865735 |
| AC092431.3 | -0.066384346 | -0.082611529 | -0.083032040 | -0.0532036895 | -0.0807819036 | -0.086606611 | -0.077423134 | -0.082611529 | -0.094537952 | -0.063762640 |
| CTC-498J12.1 | 0.136448128 | 0.114406547 | 0.103697621 | 0.0873854828 | 0.1218917530 | 0.115681196 | 0.103040883 | 0.114406547 | 0.101892816 | 0.083064718 |
| LINC01508 | 0.151301210 | 0.065497352 | 0.064645641 | 0.0518642367 | 0.0971061027 | 0.089494617 | 0.064796653 | 0.065497352 | 0.076594584 | 0.042374164 |
| LINC00707 | 0.089636592 | 0.044399709 | 0.056379336 | 0.0482472616 | 0.0696667946 | 0.063528353 | 0.054770844 | 0.044399709 | 0.050122752 | 0.043420843 |
| GATA6-AS1 | 0.094087552 | 0.069479269 | 0.062373485 | 0.0382695924 | 0.0855410961 | 0.079157502 | 0.053274793 | 0.069479269 | 0.062323661 | 0.032705033 |
| AC092580.4 | 0.486535578 | 0.446829143 | 0.453205167 | 0.4522715112 | 0.4766552315 | 0.475226079 | 0.470048411 | 0.446829143 | 0.479570862 | 0.447419834 |
| CTA-384D8.35 | 0.367392733 | 0.239890132 | 0.254585411 | 0.2282311858 | 0.2977635951 | 0.289601545 | 0.264867427 | 0.239890132 | 0.261349339 | 0.219672513 |
| CTA-384D8.34 | 0.282570541 | 0.213574739 | 0.230136538 | 0.2171957677 | 0.2492026936 | 0.247638921 | 0.235619459 | 0.213574739 | 0.229737680 | 0.214129930 |
| MIR4435-1HG | 0.089861243 | 0.022403556 | 0.051692297 | -0.0008066739 | 0.0652123246 | 0.062436433 | 0.034546443 | 0.022403556 | 0.023816494 | -0.004125197 |
| MLLT4-AS1 | -0.068362721 | -0.106506300 | -0.097794343 | -0.0870788991 | -0.0692661939 | -0.070505482 | -0.098135381 | -0.106506300 | -0.086705344 | -0.099028134 |
| EDNRB-AS1 | -0.041258339 | -0.081815235 | -0.089010152 | -0.0547154184 | -0.0805039290 | -0.085262064 | -0.084907186 | -0.081815235 | -0.088536271 | -0.069011586 |
| CTC-459F4.1 | -0.145507791 | -0.192609831 | -0.200094998 | -0.1889624667 | -0.1881323213 | -0.197099627 | -0.203050894 | -0.192609831 | -0.204674682 | -0.204836478 |
| MIAT | 0.359573660 | 0.383989883 | 0.396336593 | 0.4238596677 | 0.3960583029 | 0.401473021 | 0.409837724 | 0.383989883 | 0.400720029 | 0.429858856 |

In [50]:

```
C5.set <- cogena::gmt2list('../0.data/c5.all.v7.4.symbols.gmt')
cd.score <- gsva(tcga.obj.sub %>% GetAssayData(.) %>% as.matrix, C5.set, method = 'plage')
```

```
Registered S3 method overwritten by 'cli':
  method     from         
  print.boxx spatstat.geom
```

```
Estimating PLAGE scores for 7480 gene sets.
  |======================================================================| 100%
```

In [51]:

```
saveRDS(cd.score, file = file.path(out.data.dir, 'c5.score.rds'))
```

In [57]:

```
linc.func <- lapply(gsub('_', '-', hub.genes.sub), function(gene) {
    lnc.expr <- FetchData(tcga.obj.sub, vars = gene)
    cor.res <- cor(lnc.expr, cd.score %>% t)
    cor.res <- cor.res[1, ][order(cor.res[1, ]) %>% rev]
    cor.res <- cor.res[!is.na(cor.res)]
    cor.res[1]
})
```

In [58]:

```
linc.func
```

1. **GOBP\_NEGATIVE\_REGULATION\_OF\_IMMATURE\_T\_CELL\_PROLIFERATION:** 0.206948790924502
2. **GOBP\_CELLULAR\_RESPONSE\_TO\_VITAMIN\_D:** 0.416223729913624
3. **GOBP\_CELLULAR\_HYPEROSMOTIC\_RESPONSE:** 0.402546552398278
4. **GOBP\_GROOMING\_BEHAVIOR:** 0.416426095114604
5. **GOBP\_FOLLICLE\_STIMULATING\_HORMONE\_SECRETION:** 0.301991430407066
6. **GOBP\_PHOTORECEPTOR\_CELL\_DIFFERENTIATION:** 0.320948377654716
7. **GOBP\_PEPTIDYL\_LYSINE\_METHYLATION:** 0.480898005392247
8. **GOBP\_NEGATIVE\_REGULATION\_OF\_GENE\_EXPRESSION:** 0.845143530722819
9. **GOBP\_NEURAL\_TUBE\_PATTERNING:** 0.47825900375087
10. **GOBP\_POSITIVE\_REGULATION\_OF\_MAST\_CELL\_CHEMOTAXIS:** 0.327449125728622
11. **GOBP\_CARDIOBLAST\_PROLIFERATION:** 0.441312686537301
12. **GOBP\_TERPENOID\_CATABOLIC\_PROCESS:** 0.230180969620006
13. **GOBP\_EMBRYONIC\_SKELETAL\_SYSTEM\_DEVELOPMENT:** 0.30530199958639
14. **GOBP\_AZOLE\_TRANSMEMBRANE\_TRANSPORT:** 0.250934065437459
15. **GOBP\_PEPTIDOGLYCAN\_METABOLIC\_PROCESS:** 0.401873522376837
16. **GOBP\_O\_GLYCAN\_PROCESSING:** 0.496518334652502
17. **GOBP\_NEGATIVE\_REGULATION\_OF\_ENDOTHELIAL\_CELL\_PROLIFERATION:** 0.507480410119277
18. **GOBP\_RETINAL\_METABOLIC\_PROCESS:** 0.416012793156534
19. **GOBP\_REGULATION\_OF\_MICROVILLUS\_ASSEMBLY:** 0.485572580397275
20. **GOBP\_KERATAN\_SULFATE\_BIOSYNTHETIC\_PROCESS:** 0.286903770468006
21. **GOBP\_TYPE\_II\_PNEUMOCYTE\_DIFFERENTIATION:** 0.607400719535263
22. **GOBP\_GRANZYME\_MEDIATED\_PROGRAMMED\_CELL\_DEATH\_SIGNALING\_PATHWAY:** 0.646384047356844
23. **GOBP\_PROTEIN\_HOMOTETRAMERIZATION:** 0.4857598526001
24. **GOBP\_ANTIGEN\_PROCESSING\_AND\_PRESENTATION\_OF\_EXOGENOUS\_PEPTIDE\_ANTIGEN\_VIA\_MHC\_CLASS\_I:** 0.361833770732835
25. **GOBP\_NEGATIVE\_REGULATION\_OF\_AUTOPHAGY\_OF\_MITOCHONDRION:** 0.450563831530237
26. **GOBP\_EPITHELIAL\_CELL\_PROLIFERATION\_INVOLVED\_IN\_PROSTATE\_GLAND\_DEVELOPMENT:** 0.396615726860752
27. **GOBP\_SENSORY\_PERCEPTION\_OF\_TASTE:** 0.70072605041149
28. **GOBP\_SINGLE\_FERTILIZATION:** 0.851072749747684
29. **GOBP\_LYMPHOCYTE\_COSTIMULATION:** 0.663332734617271

In [60]:

```
linc.func.corr <- lapply(gsub('_', '-', hub.genes.sub), function(gene) {
    lnc.expr <- FetchData(tcga.obj.sub, vars = gene)
    cor.res <- cor(lnc.expr, cd.score[unlist(linc.func) %>% names, ] %>% t)
}) %>% do.call(rbind, .)
```

In [ ]:

```

```

### Volcano plots for the enrichment of immune cell types for tumors with high TILSig and low TILSig calculated based on the NES score from the gene set enrichment analysis (GSEA)¶

In [1355]:

```
createGSEAinput <- function (prefix = "GSE1009", exprSet = example_exprSet, group_list,
    destdir = ".")
{
    gct_file = paste0(prefix, ".gct")
    sink(gct_file)
    cat("#1.2\n")
    cat(paste0(nrow(exprSet), "\t", length(group_list), "\n"))
    sink()
    gct_out <- cbind(NAME = rownames(exprSet), Description = "na",
        exprSet)
    write.table(gct_out, gct_file, append = T, quote = F, row.names = F,
        sep = "\t")
    cls_file = paste0(prefix, ".cls")
    sink(cls_file)
    cat(paste0(length(group_list), " ", length(unique(group_list)),
        " 1\n"))
    cat(paste0("# ", paste(levels(group_list), collapse = " "),
        "\n"))
    cat(paste(group_list, collapse = " "))
    sink()
}
```

In [1356]:

```
#tcga.obj.sub = readRDS(file.path(out.data.dir, 'tcga.obj.sub.rds'))
```

In [1357]:

```
tcga.obj.sub@meta.data$Class <- ifelse(res.cox$group == 'Low risk', 'Low', 'High')
tcga.obj.sub@meta.data$Class <- factor(tcga.obj.sub@meta.data$Class, levels = c('High', 'Low'))
#saveRDS(tcga.obj.sub, file = file.path(out.data.dir, 'tcga.obj.sub.rds'))
```

In [1358]:

```
anno <- read.table('../0.data/gencode.gene.info.v22.tsv', sep = '\t', header = T) %>% subset(., gene_type == 'protein_coding')
head(anno)
com.genes <- intersect(rownames(tcga.obj.sub),anno$gene_name)
length(com.genes)
```

A data.frame: 6 × 12

|  | gene\_id | gene\_name | seqname | start | end | strand | gene\_type | gene\_status | havana\_gene | full\_length | exon\_length | exon\_num |
| --- | --- | --- | --- | --- | --- | --- | --- | --- | --- | --- | --- | --- |
|  | <chr> | <chr> | <chr> | <int> | <int> | <chr> | <chr> | <chr> | <chr> | <int> | <int> | <int> |
| 7 | ENSG00000206557.5 | TRIM71 | chr3 | 32818018 | 32897826 | + | protein\_coding | KNOWN | OTTHUMG00000155778.3 | 79809 | 8685 | 4 |
| 8 | ENSG00000183813.6 | CCR4 | chr3 | 32951574 | 32956349 | + | protein\_coding | KNOWN | OTTHUMG00000130752.2 | 4776 | 3095 | 2 |
| 9 | ENSG00000170266.14 | GLB1 | chr3 | 32996608 | 33097230 | - | protein\_coding | KNOWN | OTTHUMG00000155781.6 | 100623 | 4364 | 123 |
| 12 | ENSG00000188167.7 | TMPPE | chr3 | 33090421 | 33096801 | - | protein\_coding | KNOWN | OTTHUMG00000155779.1 | 6381 | 3966 | 4 |
| 14 | ENSG00000170275.13 | CRTAP | chr3 | 33113979 | 33147773 | + | protein\_coding | KNOWN | OTTHUMG00000130746.3 | 33795 | 6695 | 18 |
| 17 | ENSG00000173705.7 | SUSD5 | chr3 | 33150042 | 33219215 | - | protein\_coding | KNOWN | OTTHUMG00000155829.2 | 69174 | 5008 | 9 |

19459

In [1359]:

```
cogena::gmtlist2file(marker.lst, filename = '../0.data/markers.gmt')
```

In [1360]:

```
exprs <- FetchData(tcga.obj.sub, vars = com.genes) %>% t
head(exprs)[, 1:10]
```

A matrix: 6 × 10 of type dbl

|  | TCGA-E2-A15G-01A-11R-A12D-07 | TCGA-E2-A1B5-01A-21R-A12P-07 | TCGA-EW-A2FS-01A-11R-A17B-07 | TCGA-EW-A1P7-01A-21R-A144-07 | TCGA-LL-A5YO-01A-21R-A28M-07 | TCGA-BH-A1FN-01A-11R-A13Q-07 | TCGA-A7-A3RF-01A-11R-A22K-07 | TCGA-E9-A1NG-01A-21R-A14M-07 | TCGA-D8-A1XB-01A-11R-A14D-07 | TCGA-A2-A0CX-01A-21R-A00Z-07 |
| --- | --- | --- | --- | --- | --- | --- | --- | --- | --- | --- |
| TRIM71 | 0.004994098 | 0.008560776 | 0.01495782 | 0.008813333 | 0.01218971 | 0.01815194 | 0.003377841 | 0.00151496 | 0.007488023 | 0.08492265 |
| CCR4 | 0.399402754 | 4.750494303 | 0.43547734 | 6.244687523 | 4.78884252 | 0.42932499 | 0.047393457 | 0.38685806 | 0.840497275 | 1.16935589 |
| GLB1 | 19.763674937 | 11.738630778 | 6.94716634 | 15.097403835 | 25.52685822 | 17.64965299 | 15.125397977 | 17.07992986 | 15.483451471 | 39.49689988 |
| TMPPE | 0.935061668 | 0.257770344 | 0.30708364 | 0.598299950 | 0.38038670 | 0.64168301 | 0.380946119 | 0.29526244 | 0.934671752 | 1.80779207 |
| CRTAP | 27.760487837 | 29.451397079 | 14.64504293 | 26.032892907 | 27.26545987 | 15.54798693 | 23.846079661 | 37.24956500 | 29.500622634 | 40.64311861 |
| SUSD5 | 0.775149665 | 0.790566222 | 0.28534251 | 1.230386459 | 0.77159911 | 3.58866879 | 0.038076592 | 0.63580210 | 0.904685611 | 0.50690001 |

In [1361]:

```
es.scores <- gsva(exprs, marker.lst, method = 'ssgsea')
```

```
Estimating ssGSEA scores for 28 gene sets.
  |======================================================================| 100%
```

In [1362]:

```
label <- tcga.obj.sub@meta.data$Class
names(label) <- colnames(tcga.obj.sub)
group.list <- factor(label)

design <- model.matrix(~0 + group.list)
colnames(design) <- levels(group.list)
rownames(design) <- colnames(es.scores)
contrast.matrix <- makeContrasts('High-Low',levels = design)
fit.lm <- lmFit(es.scores, design)
fit2 <- contrasts.fit(fit.lm, contrast.matrix)
fit2 <- eBayes(fit2)

DEgeneSets <- topTable(fit2, coef=1, number=Inf, p.value=1e-2, adjust = "BH")
```

In [1364]:

```
createGSEAinput(prefix = ".expr", exprSet = exprs %>% quantileNorm(.), tcga.obj.sub@meta.data$Class, destdir = ".")
```

In [1365]:

```
gesa.res <- GSEA::GSEA('.expr.gct', '.expr.cls', gs.db = '../0.data/markers.gmt', output.directory = file.path(out.figs.dir, 'L_vs_H'), collapse.mode = 'max')
```

```
[1] " *** Running Gene Set Enrichment Analysis..."
[1] "Number of genes:" "19459"           
[1] "Number of Gene Sets:" "52"                  
[1] "Number of samples:" "1109"              
[1] "Original number of Gene Sets:" "56"                           
[1] "Maximum gene set size:" "77"                    
[1] "Minimum gene set size:" "14"                    
[1] "Number of gene annotation file entries: 19459"
[1] "Computing ranked list for actual and permuted phenotypes.......permutations:  1 -- 100"
[1] "Computing ranked list for actual and permuted phenotypes.......permutations:  101 -- 200"
[1] "Computing ranked list for actual and permuted phenotypes.......permutations:  201 -- 300"
[1] "Computing ranked list for actual and permuted phenotypes.......permutations:  301 -- 400"
[1] "Computing ranked list for actual and permuted phenotypes.......permutations:  401 -- 500"
[1] "Computing ranked list for actual and permuted phenotypes.......permutations:  501 -- 600"
[1] "Computing ranked list for actual and permuted phenotypes.......permutations:  601 -- 700"
[1] "Computing ranked list for actual and permuted phenotypes.......permutations:  701 -- 800"
[1] "Computing ranked list for actual and permuted phenotypes.......permutations:  801 -- 900"
[1] "Computing ranked list for actual and permuted phenotypes.......permutations:  901 -- 1000"
[1] "Computing observed enrichment for gene set: 1 Activated B cell"
[1] "Computing observed enrichment for gene set: 2 Activated CD4 T cell"
[1] "Computing observed enrichment for gene set: 3 Activated CD8 T cell"
[1] "Computing observed enrichment for gene set: 4 Activated dendritic cell"
[1] "Computing observed enrichment for gene set: 5 CD56bright natural killer cell"
[1] "Computing observed enrichment for gene set: 6 Central memory CD4 T cell"
[1] "Computing observed enrichment for gene set: 7 Central memory CD8 T cell"
[1] "Computing observed enrichment for gene set: 8 Effector memeory CD4 T cell"
[1] "Computing observed enrichment for gene set: 9 Effector memeory CD8 T cell"
[1] "Computing observed enrichment for gene set: 10 Eosinophil"
[1] "Computing observed enrichment for gene set: 11 Gamma delta T cell"
[1] "Computing observed enrichment for gene set: 12 Immature  B cell"
[1] "Computing observed enrichment for gene set: 13 Immature dendritic cell"
[1] "Computing observed enrichment for gene set: 14 Macrophage"
[1] "Computing observed enrichment for gene set: 15 Mast cell"
[1] "Computing observed enrichment for gene set: 16 MDSC"
[1] "Computing observed enrichment for gene set: 17 Monocyte"
[1] "Computing observed enrichment for gene set: 18 Natural killer cell"
[1] "Computing observed enrichment for gene set: 19 Natural killer T cell"
[1] "Computing observed enrichment for gene set: 20 Neutrophil"
[1] "Computing observed enrichment for gene set: 21 Plasmacytoid dendritic cell"
[1] "Computing observed enrichment for gene set: 22 Regulatory T cell"
[1] "Computing observed enrichment for gene set: 23 T follicular helper cell"
[1] "Computing observed enrichment for gene set: 24 Type 1 T helper cell"
[1] "Computing observed enrichment for gene set: 25 Type 17 T helper cell"
[1] "Computing observed enrichment for gene set: 26 Type 2 T helper cell"
[1] "Computing observed enrichment for gene set: 27 Activated B cell"
[1] "Computing observed enrichment for gene set: 28 Activated CD4 T cell"
[1] "Computing observed enrichment for gene set: 29 Activated CD8 T cell"
[1] "Computing observed enrichment for gene set: 30 Activated dendritic cell"
[1] "Computing observed enrichment for gene set: 31 CD56bright natural killer cell"
[1] "Computing observed enrichment for gene set: 32 Central memory CD4 T cell"
[1] "Computing observed enrichment for gene set: 33 Central memory CD8 T cell"
[1] "Computing observed enrichment for gene set: 34 Effector memeory CD4 T cell"
[1] "Computing observed enrichment for gene set: 35 Effector memeory CD8 T cell"
[1] "Computing observed enrichment for gene set: 36 Eosinophil"
[1] "Computing observed enrichment for gene set: 37 Gamma delta T cell"
[1] "Computing observed enrichment for gene set: 38 Immature  B cell"
[1] "Computing observed enrichment for gene set: 39 Immature dendritic cell"
[1] "Computing observed enrichment for gene set: 40 Macrophage"
[1] "Computing observed enrichment for gene set: 41 Mast cell"
[1] "Computing observed enrichment for gene set: 42 MDSC"
[1] "Computing observed enrichment for gene set: 43 Monocyte"
[1] "Computing observed enrichment for gene set: 44 Natural killer cell"
[1] "Computing observed enrichment for gene set: 45 Natural killer T cell"
[1] "Computing observed enrichment for gene set: 46 Neutrophil"
[1] "Computing observed enrichment for gene set: 47 Plasmacytoid dendritic cell"
[1] "Computing observed enrichment for gene set: 48 Regulatory T cell"
[1] "Computing observed enrichment for gene set: 49 T follicular helper cell"
[1] "Computing observed enrichment for gene set: 50 Type 1 T helper cell"
[1] "Computing observed enrichment for gene set: 51 Type 17 T helper cell"
[1] "Computing observed enrichment for gene set: 52 Type 2 T helper cell"
[1] "Computing random permutations' enrichment for gene set: 1 Activated B cell"
[1] "Computing random permutations' enrichment for gene set: 2 Activated CD4 T cell"
[1] "Computing random permutations' enrichment for gene set: 3 Activated CD8 T cell"
[1] "Computing random permutations' enrichment for gene set: 4 Activated dendritic cell"
[1] "Computing random permutations' enrichment for gene set: 5 CD56bright natural killer cell"
[1] "Computing random permutations' enrichment for gene set: 6 Central memory CD4 T cell"
[1] "Computing random permutations' enrichment for gene set: 7 Central memory CD8 T cell"
[1] "Computing random permutations' enrichment for gene set: 8 Effector memeory CD4 T cell"
[1] "Computing random permutations' enrichment for gene set: 9 Effector memeory CD8 T cell"
[1] "Computing random permutations' enrichment for gene set: 10 Eosinophil"
[1] "Computing random permutations' enrichment for gene set: 11 Gamma delta T cell"
[1] "Computing random permutations' enrichment for gene set: 12 Immature  B cell"
[1] "Computing random permutations' enrichment for gene set: 13 Immature dendritic cell"
[1] "Computing random permutations' enrichment for gene set: 14 Macrophage"
[1] "Computing random permutations' enrichment for gene set: 15 Mast cell"
[1] "Computing random permutations' enrichment for gene set: 16 MDSC"
[1] "Computing random permutations' enrichment for gene set: 17 Monocyte"
[1] "Computing random permutations' enrichment for gene set: 18 Natural killer cell"
[1] "Computing random permutations' enrichment for gene set: 19 Natural killer T cell"
[1] "Computing random permutations' enrichment for gene set: 20 Neutrophil"
[1] "Computing random permutations' enrichment for gene set: 21 Plasmacytoid dendritic cell"
[1] "Computing random permutations' enrichment for gene set: 22 Regulatory T cell"
[1] "Computing random permutations' enrichment for gene set: 23 T follicular helper cell"
[1] "Computing random permutations' enrichment for gene set: 24 Type 1 T helper cell"
[1] "Computing random permutations' enrichment for gene set: 25 Type 17 T helper cell"
[1] "Computing random permutations' enrichment for gene set: 26 Type 2 T helper cell"
[1] "Computing random permutations' enrichment for gene set: 27 Activated B cell"
[1] "Computing random permutations' enrichment for gene set: 28 Activated CD4 T cell"
[1] "Computing random permutations' enrichment for gene set: 29 Activated CD8 T cell"
[1] "Computing random permutations' enrichment for gene set: 30 Activated dendritic cell"
[1] "Computing random permutations' enrichment for gene set: 31 CD56bright natural killer cell"
[1] "Computing random permutations' enrichment for gene set: 32 Central memory CD4 T cell"
[1] "Computing random permutations' enrichment for gene set: 33 Central memory CD8 T cell"
[1] "Computing random permutations' enrichment for gene set: 34 Effector memeory CD4 T cell"
[1] "Computing random permutations' enrichment for gene set: 35 Effector memeory CD8 T cell"
[1] "Computing random permutations' enrichment for gene set: 36 Eosinophil"
[1] "Computing random permutations' enrichment for gene set: 37 Gamma delta T cell"
[1] "Computing random permutations' enrichment for gene set: 38 Immature  B cell"
[1] "Computing random permutations' enrichment for gene set: 39 Immature dendritic cell"
[1] "Computing random permutations' enrichment for gene set: 40 Macrophage"
[1] "Computing random permutations' enrichment for gene set: 41 Mast cell"
[1] "Computing random permutations' enrichment for gene set: 42 MDSC"
[1] "Computing random permutations' enrichment for gene set: 43 Monocyte"
[1] "Computing random permutations' enrichment for gene set: 44 Natural killer cell"
[1] "Computing random permutations' enrichment for gene set: 45 Natural killer T cell"
[1] "Computing random permutations' enrichment for gene set: 46 Neutrophil"
[1] "Computing random permutations' enrichment for gene set: 47 Plasmacytoid dendritic cell"
[1] "Computing random permutations' enrichment for gene set: 48 Regulatory T cell"
[1] "Computing random permutations' enrichment for gene set: 49 T follicular helper cell"
[1] "Computing random permutations' enrichment for gene set: 50 Type 1 T helper cell"
[1] "Computing random permutations' enrichment for gene set: 51 Type 17 T helper cell"
[1] "Computing random permutations' enrichment for gene set: 52 Type 2 T helper cell"
[1] "Computing nominal p-values..."
[1] "Computing rescaling normalization for each gene set null..."
[1] "Computing FWER p-values..."
[1] "Computing FDR q-values..."
[1] "Producing result tables and plots..."
[1] -0.002782127  0.009273756  0.009273756  0.009273756  0.008699307
```

In [1366]:

```
gesa.res
```

$report1
:   A data.frame: 52 × 13

    |  | GS | SIZE | SOURCE | ES | NES | NOM p-val | FDR q-val | FWER p-val | Tag % | Gene % | Signal | FDR (median) | glob.p.val |
    | --- | --- | --- | --- | --- | --- | --- | --- | --- | --- | --- | --- | --- | --- |
    |  | <chr> | <chr> | <chr> | <chr> | <chr> | <chr> | <chr> | <chr> | <chr> | <chr> | <chr> | <chr> | <chr> |
    | 1 | Type 1 T helper cell | 76 | 77 | 0.58389 | 1.9872 | 0.003591 | 0.0054323 | 0.003006 | 0.382 | 0.127 | 0.335 | 0 | 0.003 |
    | 2 | Type 1 T helper cell | 76 | 77 | 0.58389 | 1.9872 | 0.003591 | 0.0054323 | 0.003006 | 0.382 | 0.127 | 0.335 | 0 | 0.003 |
    | 3 | Effector memeory CD8 T cell | 25 | 25 | 0.76655 | 1.9722 | 0 | 0.0048923 | 0.007014 | 0.52 | 0.0799 | 0.479 | 0 | 0.001 |
    | 4 | Effector memeory CD8 T cell | 25 | 25 | 0.76655 | 1.9722 | 0 | 0.0048923 | 0.007014 | 0.52 | 0.0799 | 0.479 | 0 | 0.001 |
    | 5 | Activated CD8 T cell | 26 | 26 | 0.77928 | 1.9526 | 0 | 0.0044105 | 0.009018 | 0.462 | 0.00992 | 0.458 | 0 | 0.001 |
    | 6 | Activated CD8 T cell | 26 | 26 | 0.77928 | 1.9526 | 0 | 0.0044105 | 0.009018 | 0.462 | 0.00992 | 0.458 | 0 | 0.001 |
    | 7 | Natural killer cell | 33 | 35 | 0.56798 | 1.8672 | 0.005618 | 0.014308 | 0.03607 | 0.455 | 0.18 | 0.373 | 0 | 0.004 |
    | 8 | Natural killer cell | 33 | 35 | 0.56798 | 1.8672 | 0.005618 | 0.014308 | 0.03607 | 0.455 | 0.18 | 0.373 | 0 | 0.004 |
    | 9 | Activated B cell | 24 | 26 | 0.89044 | 1.8434 | 0 | 0.013214 | 0.04008 | 0.833 | 0.0734 | 0.773 | 0 | 0.001 |
    | 10 | Activated B cell | 24 | 26 | 0.89044 | 1.8434 | 0 | 0.013214 | 0.04008 | 0.833 | 0.0734 | 0.773 | 0 | 0.001 |
    | 11 | Mast cell | 17 | 17 | 0.70573 | 1.8363 | 0.005291 | 0.012243 | 0.04509 | 0.588 | 0.115 | 0.521 | 0 | 0.001 |
    | 12 | Mast cell | 17 | 17 | 0.70573 | 1.8363 | 0.005291 | 0.012243 | 0.04509 | 0.588 | 0.115 | 0.521 | 0 | 0.001 |
    | 13 | MDSC | 20 | 20 | 0.78245 | 1.8288 | 0.007505 | 0.010934 | 0.04509 | 0.5 | 0.0666 | 0.467 | 0 | 0 |
    | 14 | MDSC | 20 | 20 | 0.78245 | 1.8288 | 0.007505 | 0.010934 | 0.04509 | 0.5 | 0.0666 | 0.467 | 0 | 0 |
    | 15 | Natural killer T cell | 43 | 44 | 0.58031 | 1.8112 | 0.006897 | 0.013522 | 0.05411 | 0.465 | 0.137 | 0.402 | 0 | 0.002 |
    | 16 | Natural killer T cell | 43 | 44 | 0.58031 | 1.8112 | 0.006897 | 0.013522 | 0.05411 | 0.465 | 0.137 | 0.402 | 0 | 0.002 |
    | 17 | Immature B cell | 18 | 19 | 0.83065 | 1.7708 | 0.001802 | 0.01712 | 0.07615 | 0.833 | 0.109 | 0.743 | 0 | 0.001 |
    | 18 | Immature B cell | 18 | 19 | 0.83065 | 1.7708 | 0.001802 | 0.01712 | 0.07615 | 0.833 | 0.109 | 0.743 | 0 | 0.001 |
    | 19 | Macrophage | 31 | 33 | 0.57574 | 1.6881 | 0.02541 | 0.035702 | 0.1513 | 0.419 | 0.0921 | 0.381 | 0 | 0.001 |
    | 20 | Macrophage | 31 | 33 | 0.57574 | 1.6881 | 0.02541 | 0.035702 | 0.1513 | 0.419 | 0.0921 | 0.381 | 0 | 0.001 |
    | 21 | Regulatory T cell | 19 | 20 | 0.65171 | 1.6338 | 0.0411 | 0.051103 | 0.2064 | 0.632 | 0.129 | 0.551 | 0 | 0.004 |
    | 22 | Regulatory T cell | 19 | 20 | 0.65171 | 1.6338 | 0.0411 | 0.051103 | 0.2064 | 0.632 | 0.129 | 0.551 | 0 | 0.004 |
    | 23 | T follicular helper cell | 34 | 36 | 0.55154 | 1.6255 | 0.04945 | 0.048768 | 0.2104 | 0.441 | 0.097 | 0.399 | 0 | 0.001 |
    | 24 | T follicular helper cell | 34 | 36 | 0.55154 | 1.6255 | 0.04945 | 0.048768 | 0.2104 | 0.441 | 0.097 | 0.399 | 0 | 0.001 |
    | 25 | Activated CD4 T cell | 25 | 25 | 0.61481 | 1.5541 | 0.06818 | 0.073425 | 0.2836 | 0.4 | 0.0552 | 0.378 | 0 | 0.005 |
    | 26 | Activated CD4 T cell | 25 | 25 | 0.61481 | 1.5541 | 0.06818 | 0.073425 | 0.2836 | 0.4 | 0.0552 | 0.378 | 0 | 0.005 |
    | 27 | Plasmacytoid dendritic cell | 31 | 32 | 0.45291 | 1.4973 | 0.04182 | 0.093117 | 0.3587 | 0.355 | 0.152 | 0.301 | 0 | 0.004 |
    | 28 | Plasmacytoid dendritic cell | 31 | 32 | 0.45291 | 1.4973 | 0.04182 | 0.093117 | 0.3587 | 0.355 | 0.152 | 0.301 | 0 | 0.004 |
    | 29 | Central memory CD8 T cell | 21 | 21 | 0.46711 | 1.4713 | 0.05106 | 0.1008 | 0.4008 | 0.333 | 0.105 | 0.299 | 0 | 0.003 |
    | 30 | Central memory CD8 T cell | 21 | 21 | 0.46711 | 1.4713 | 0.05106 | 0.1008 | 0.4008 | 0.333 | 0.105 | 0.299 | 0 | 0.003 |
    | 31 | Central memory CD4 T cell | 26 | 28 | 0.46612 | 1.469 | 0.05243 | 0.095288 | 0.4018 | 0.308 | 0.108 | 0.275 | 0 | 0.001 |
    | 32 | Central memory CD4 T cell | 26 | 28 | 0.46612 | 1.469 | 0.05243 | 0.095288 | 0.4018 | 0.308 | 0.108 | 0.275 | 0 | 0.001 |
    | 33 | Activated dendritic cell | 35 | 35 | 0.47191 | 1.4671 | 0.08688 | 0.090812 | 0.4078 | 0.2 | 0.0329 | 0.194 | 0 | 0.001 |
    | 34 | Activated dendritic cell | 35 | 35 | 0.47191 | 1.4671 | 0.08688 | 0.090812 | 0.4078 | 0.2 | 0.0329 | 0.194 | 0 | 0.001 |
    | 35 | Eosinophil | 16 | 17 | 0.50756 | 1.389 | 0.1089 | 0.13069 | 0.5291 | 0.625 | 0.273 | 0.455 | 0.068783 | 0.005 |
    | 36 | Eosinophil | 16 | 17 | 0.50756 | 1.389 | 0.1089 | 0.13069 | 0.5291 | 0.625 | 0.273 | 0.455 | 0.068783 | 0.005 |
    | 37 | Gamma delta T cell | 30 | 31 | 0.40846 | 1.2839 | 0.2099 | 0.20126 | 0.6764 | 0.3 | 0.073 | 0.279 | 0.13684 | 0.01 |
    | 38 | Gamma delta T cell | 30 | 31 | 0.40846 | 1.2839 | 0.2099 | 0.20126 | 0.6764 | 0.3 | 0.073 | 0.279 | 0.13684 | 0.01 |
    | 39 | Monocyte | 20 | 21 | 0.39912 | 1.2452 | 0.188 | 0.22162 | 0.7264 | 0.15 | 0.0229 | 0.147 | 0.1625 | 0.009 |
    | 40 | Monocyte | 20 | 21 | 0.39912 | 1.2452 | 0.188 | 0.22162 | 0.7264 | 0.15 | 0.0229 | 0.147 | 0.1625 | 0.009 |
    | 41 | Type 2 T helper cell | 29 | 29 | 0.34808 | 1.1012 | 0.3416 | 0.35698 | 0.8707 | 0.241 | 0.117 | 0.213 | 0.30952 | 0.02 |
    | 42 | Type 2 T helper cell | 29 | 29 | 0.34808 | 1.1012 | 0.3416 | 0.35698 | 0.8707 | 0.241 | 0.117 | 0.213 | 0.30952 | 0.02 |
    | 43 | Immature dendritic cell | 23 | 23 | 0.31194 | 1.0215 | 0.4283 | 0.44061 | 0.9268 | 0.391 | 0.197 | 0.315 | 0.41364 | 0.028 |
    | 44 | Immature dendritic cell | 23 | 23 | 0.31194 | 1.0215 | 0.4283 | 0.44061 | 0.9268 | 0.391 | 0.197 | 0.315 | 0.41364 | 0.028 |
    | 45 | Type 17 T helper cell | 26 | 27 | 0.29708 | 0.92938 | 0.5649 | 0.54442 | 0.9659 | 0.154 | 0.0873 | 0.141 | 0.53547 | 0.041 |
    | 46 | Type 17 T helper cell | 26 | 27 | 0.29708 | 0.92938 | 0.5649 | 0.54442 | 0.9659 | 0.154 | 0.0873 | 0.141 | 0.53547 | 0.041 |
    | 47 | Effector memeory CD4 T cell | 27 | 27 | 0.26353 | 0.84077 | 0.6757 | 0.64767 | 0.983 | 0.148 | 0.047 | 0.141 | 0.65 | 0.056 |
    | 48 | Effector memeory CD4 T cell | 27 | 27 | 0.26353 | 0.84077 | 0.6757 | 0.64767 | 0.983 | 0.148 | 0.047 | 0.141 | 0.65 | 0.056 |
    | 49 | CD56bright natural killer cell | 34 | 40 | 0.22268 | 0.80326 | 0.7632 | 0.67394 | 0.984 | 0.294 | 0.175 | 0.243 | 0.69333 | 0.041 |
    | 50 | CD56bright natural killer cell | 34 | 40 | 0.22268 | 0.80326 | 0.7632 | 0.67394 | 0.984 | 0.294 | 0.175 | 0.243 | 0.69333 | 0.041 |
    | 51 | Neutrophil | 17 | 18 | 0.23867 | 0.66298 | 0.8784 | 0.82664 | 0.995 | 0.353 | 0.245 | 0.267 | 0.84615 | 0.188 |
    | 52 | Neutrophil | 17 | 18 | 0.23867 | 0.66298 | 0.8784 | 0.82664 | 0.995 | 0.353 | 0.245 | 0.267 | 0.84615 | 0.188 |

$report2
:   A data.frame: 1 × 13

    |  | GS | SIZE | SOURCE | ES | NES | NOM p-val | FDR q-val | FWER p-val | Tag % | Gene % | Signal | FDR (median) | glob.p.val |
    | --- | --- | --- | --- | --- | --- | --- | --- | --- | --- | --- | --- | --- | --- |
    |  | <chr> | <chr> | <chr> | <chr> | <chr> | <chr> | <chr> | <chr> | <chr> | <chr> | <chr> | <chr> | <chr> |
    | 1 | Neutrophil | 17 | 18 | 0.23867 | 0.66298 | 0.8784 | 0.82664 | 0.995 | 0.353 | 0.245 | 0.267 | 0.84615 | 0.188 |

In [1367]:

```
saveRDS(gesa.res, file = file.path(out.data.dir, 'gesa.res.rds'))
```

In [1368]:

```
plot.df <- rbind.data.frame(gesa.res$report1, gesa.res$report2)[, c('GS', 'NES', 'NOM p-val')]
plot.df <- cbind.data.frame(plot.df, Class = c(rep('Low', dim(gesa.res$report1)[1]), rep('High', dim(gesa.res$report2)[1])))
colnames(plot.df)[3] <- 'Pval'
plot.df[, 2] <- as.numeric(plot.df[, 2])
plot.df[, 3] <- as.numeric(plot.df[, 3])
head(plot.df)
```

A data.frame: 6 × 4

|  | GS | NES | Pval | Class |
| --- | --- | --- | --- | --- |
|  | <chr> | <dbl> | <dbl> | <chr> |
| 1 | Type 1 T helper cell | 1.9872 | 0.003591 | Low |
| 2 | Type 1 T helper cell | 1.9872 | 0.003591 | Low |
| 3 | Effector memeory CD8 T cell | 1.9722 | 0.000000 | Low |
| 4 | Effector memeory CD8 T cell | 1.9722 | 0.000000 | Low |
| 5 | Activated CD8 T cell | 1.9526 | 0.000000 | Low |
| 6 | Activated CD8 T cell | 1.9526 | 0.000000 | Low |

In [1369]:

```
cols <- PAIRWISE_COLORS %>% unlist
names(cols) <- c('High', 'Low')
ggplot(plot.df, aes(x = -NES, y = -log10(Pval), color = Class)) + 
    geom_point(size = 8, alpha = 0.6) +     
    theme_classic(base_size = 20) + 
    xlab('Immune cell types enrichment (NES)') + 
    geom_vline(xintercept = 0, lty = 'dotted', lwd = 1.5) + 
    scale_color_manual(values = cols) + 
    ggrepel::geom_text_repel(
        data = plot.df,
        aes(label = GS),
        size = 5,
        box.padding = unit(0.35, "lines"),
        point.padding = unit(0.3, "lines")
      ) + ylab('-log10(P-value)')
ggsave(file.path(out.figs.dir, 'valcon.plot.immmuneCells.pdf'), width = 12, height = 7)
```

### Classification using lincRNA genes¶

In [50]:

```
hub.genes.sub.sub <- setdiff(hub.genes.sub, c('LINC01508', 'AL022344.7', 'AF131215.8', 'GATA6_AS1'))
expr.linc <- FetchData(tcga.obj.sub, vars = hub.genes.sub.sub)
head(expr.linc)[, 1:10]
```

A data.frame: 6 × 10

|  | LINC01235 | LINC00987 | LINC00398 | LINC00667 | ZNF888 | AC016995.3 | SFTA1P | LINC00958 | LINC00327 | LINC00346 |
| --- | --- | --- | --- | --- | --- | --- | --- | --- | --- | --- |
|  | <dbl> | <dbl> | <dbl> | <dbl> | <dbl> | <dbl> | <dbl> | <dbl> | <dbl> | <dbl> |
| TCGA-E2-A15G-01A-11R-A12D-07 | 0.2441457 | 0.5176073 | 0.5505497 | 4.766416 | 1.4169279 | 0.20852759 | 0.2776808 | 0.81744329 | 0.026560770 | 1.3050199 |
| TCGA-E2-A1B5-01A-21R-A12P-07 | 0.8947440 | 1.9249279 | 0.1436127 | 2.433822 | 0.3036085 | 0.87260722 | 0.4759945 | 0.29544300 | 0.005691239 | 0.7654584 |
| TCGA-EW-A2FS-01A-11R-A17B-07 | 0.5106076 | 1.2743872 | 0.1792338 | 3.283357 | 0.7662489 | 0.49597481 | 0.3742567 | 0.19911069 | 0.079552150 | 1.0549247 |
| TCGA-EW-A1P7-01A-21R-A144-07 | 7.2205520 | 2.5081097 | 0.1056068 | 6.786185 | 0.8682372 | 4.29692959 | 0.5880445 | 0.39106162 | 0.099605365 | 5.3635635 |
| TCGA-LL-A5YO-01A-21R-A28M-07 | 3.8631820 | 0.3640270 | 0.1168517 | 5.908502 | 1.3449608 | 0.35927927 | 0.2033309 | 0.00000000 | 0.056726376 | 0.2492494 |
| TCGA-BH-A1FN-01A-11R-A13Q-07 | 1.4425567 | 0.2550964 | 0.0994321 | 3.632807 | 3.0655222 | 0.07642999 | 0.1441829 | 0.04091075 | 0.055165634 | 2.2107969 |

In [51]:

```
results.clust <- ConsensusClusterPlus(t(log2(1 + expr.linc) %>% scale), maxK=6, reps=50, pItem=0.8, pFeature=1, title=file.path(out.figs.dir, 'Consens'), clusterAlg="pam", distance="pearson", seed=123456, plot="pdf")
```

```
end fraction

clustered

clustered

clustered

clustered

clustered
```

In [52]:

```
sub.clinical$Groups <- as.character(results.clust[[4]]$consensusClass)
res.cox.bak <- OS.analysis(
    sub.clinical, 
    c('#8A0E1A', '#3574CE', '#ED8141', 'green', 'red'), 
    F, 
    cutoff = F, 
    prefix = 'TCGA_selected_genesets_OS_Clusters.pdf',
    out.figs.dir = out.figs.dir,
    title = sprintf('TCGA-BRAC data (n = %g)', dim(sub.clinical)[1])
)
```

In [53]:

```
sub.clinical$Score <- as.vector(risk.socres)
```

In [54]:

```
comparisons <- pairwiseComp(c('1', '2', '3', '4'))
ggplot(sub.clinical, aes(x = Groups, y = Score, fill = Groups)) + geom_boxplot() + theme_classic(base_size = 20) + geom_signif(comparisons = comparisons, step_increase = 0.1) + ylab('Risk score') + xlab('Cluster')
ggsave(file.path(out.figs.dir, 'clusters.parewise.sig.pdf'))
```

```
Saving 6.67 x 6.67 in image
```

In [324]:

```
class.lab <- class.lab.tmp <- as.character(results.clust[[4]]$consensusClass)
```

In [325]:

```
class.lab.tmp[class.lab.tmp %in% c('2', '3')] <- '2_3'
table(class.lab.tmp)
```

```
class.lab.tmp
  1 2_3   4 
389 482 238
```

In [57]:

```
sub.clinical$Groups <- class.lab.tmp
res.cox.bak <- OS.analysis(
    sub.clinical, 
    c('#8A0E1A', '#3574CE', '#ED8141', 'green', 'red'), 
    F, 
    cutoff = F, 
    prefix = 'TCGA_selected_genesets_OS_Clusters_merged.pdf',
    out.figs.dir = out.figs.dir,
    title = sprintf('TCGA-BRAC data (n = %g)', dim(sub.clinical)[1])
)
```

In [326]:

```
Idents(tcga.obj.sub) <- class.lab.tmp
```

In [59]:

```
anno <- read.table('../0.data/gencode.gene.info.v22.tsv', sep = '\t', header = T) %>% subset(., gene_type == 'protein_coding')
head(anno)
com.genes <- intersect(rownames(tcga.obj.sub),anno$gene_name)
length(com.genes)
```

A data.frame: 6 × 12

|  | gene\_id | gene\_name | seqname | start | end | strand | gene\_type | gene\_status | havana\_gene | full\_length | exon\_length | exon\_num |
| --- | --- | --- | --- | --- | --- | --- | --- | --- | --- | --- | --- | --- |
|  | <chr> | <chr> | <chr> | <int> | <int> | <chr> | <chr> | <chr> | <chr> | <int> | <int> | <int> |
| 7 | ENSG00000206557.5 | TRIM71 | chr3 | 32818018 | 32897826 | + | protein\_coding | KNOWN | OTTHUMG00000155778.3 | 79809 | 8685 | 4 |
| 8 | ENSG00000183813.6 | CCR4 | chr3 | 32951574 | 32956349 | + | protein\_coding | KNOWN | OTTHUMG00000130752.2 | 4776 | 3095 | 2 |
| 9 | ENSG00000170266.14 | GLB1 | chr3 | 32996608 | 33097230 | - | protein\_coding | KNOWN | OTTHUMG00000155781.6 | 100623 | 4364 | 123 |
| 12 | ENSG00000188167.7 | TMPPE | chr3 | 33090421 | 33096801 | - | protein\_coding | KNOWN | OTTHUMG00000155779.1 | 6381 | 3966 | 4 |
| 14 | ENSG00000170275.13 | CRTAP | chr3 | 33113979 | 33147773 | + | protein\_coding | KNOWN | OTTHUMG00000130746.3 | 33795 | 6695 | 18 |
| 17 | ENSG00000173705.7 | SUSD5 | chr3 | 33150042 | 33219215 | - | protein\_coding | KNOWN | OTTHUMG00000155829.2 | 69174 | 5008 | 9 |

19459

In [61]:

```
tcga.obj.sub.sub <- subset(tcga.obj.sub, features = com.genes)
dim(tcga.obj.sub.sub)
```

1. 19459
2. 1109

In [490]:

```
label <- ifelse(res.cox$group == 'High risk', 'High', 'Low')
tcga.obj.sub.sub@meta.data$Group <- label
names(label) <- colnames(tcga.obj.sub.sub)
group.list <- factor(label)

design <- model.matrix(~0 + group.list)
colnames(design) <- levels(group.list)
rownames(design) <- colnames(tcga.obj.sub.sub)
contrast.matrix <- makeContrasts('High-Low',levels = design)
fit.lm <- lmFit(GetAssayData(tcga.obj.sub.sub) %>% as.matrix, design)
fit2 <- contrasts.fit(fit.lm, contrast.matrix)
fit2 <- eBayes(fit2)
DEgeneSets <- topTable(fit2, coef=1, number=Inf, p.value=1e-2, adjust="BH")

DEgeneSets.sub <- subset(DEgeneSets, logFC > 4 | logFC < -4)
head(DEgeneSets.sub)
dim(DEgeneSets.sub)
```

A data.frame: 6 × 6

|  | logFC | AveExpr | t | P.Value | adj.P.Val | B |
| --- | --- | --- | --- | --- | --- | --- |
|  | <dbl> | <dbl> | <dbl> | <dbl> | <dbl> | <dbl> |
| IMPAD1 | 5.930885 | 22.62325 | 6.282567 | 4.777469e-10 | 1.328068e-06 | 12.442014 |
| TNFRSF1B | -4.525251 | 11.43782 | -6.209707 | 7.495137e-10 | 1.745249e-06 | 12.018339 |
| SDC1 | 46.049912 | 115.59689 | 6.197639 | 8.071966e-10 | 1.745249e-06 | 11.948607 |
| CD74 | -257.495588 | 644.58295 | -6.037343 | 2.135010e-09 | 2.596572e-06 | 11.034386 |
| CD2 | -6.650829 | 10.72835 | -5.865746 | 5.897856e-09 | 3.702141e-06 | 10.080531 |
| FUCA1 | -7.043216 | 29.62709 | -5.767137 | 1.045114e-08 | 5.496450e-06 | 9.544063 |

1. 195
2. 6

In [491]:

```
x <- FetchData(tcga.obj.sub.sub, vars = rownames(DEgeneSets.sub)) %>% {log2(1 + .)} %>% as.matrix
y <- factor(label)

alpha1_fit <- glmnet(x, y, alpha = 1, family = 'binomial')
alpha1.fit <- cv.glmnet(x, y, type.measure = "class", alpha = 1, family = "binomial")
print(alpha1.fit)
c <- as.data.frame(coef(alpha1_fit, s=alpha1.fit$lambda.1se))
names(c) <-'coef'
lasso.cox.gene.list <- rownames(subset(c, abs(coef)>0))[-1]
length(lasso.cox.gene.list)
```

```
Call:  cv.glmnet(x = x, y = y, type.measure = "class", alpha = 1, family = "binomial") 

Measure: Misclassification Error 

     Lambda Index Measure       SE Nonzero
min 0.01126    23  0.1515 0.009405      52
1se 0.02369    15  0.1596 0.011735      37
```

37

In [488]:

```
DoHeatmap(tcga.obj.sub.sub %>% NormalizeData %>% ScaleData, features = lasso.cox.gene.list, group.by = 'Group')
```

```
Centering and scaling data matrix
```

In [242]:

```
source('CIBERSORT.R')
load('../0.data/LM22.Rdata')
```

In [243]:

```
res.cib <- CIBERSORT(LM22, GetAssayData(tcga.obj.sub) %>% as.matrix)
```

In [247]:

```
colnames(res.cib)[1:22]
```

1. 'B cells naive'
2. 'B cells memory'
3. 'Plasma cells'
4. 'T cells CD8'
5. 'T cells CD4 naive'
6. 'T cells CD4 memory resting'
7. 'T cells CD4 memory activated'
8. 'T cells follicular helper'
9. 'T cells regulatory (Tregs)'
10. 'T cells gamma delta'
11. 'NK cells resting'
12. 'NK cells activated'
13. 'Monocytes'
14. 'Macrophages M0'
15. 'Macrophages M1'
16. 'Macrophages M2'
17. 'Dendritic cells resting'
18. 'Dendritic cells activated'
19. 'Mast cells resting'
20. 'Mast cells activated'
21. 'Eosinophils'
22. 'Neutrophils'

In [501]:

```
data <- res.cib[, 1:22] %>% as.data.frame
data$label <- label
```

In [502]:

```
kk = tidyr::gather(data, 'Label', 'Frac', -label)
head(kk)
kk$Label <- factor(kk$Label, levels = colnames(LM22))
```

A data.frame: 6 × 3

|  | label | Label | Frac |
| --- | --- | --- | --- |
|  | <chr> | <chr> | <dbl> |
| 1 | Low | B cells naive | 0.07013699 |
| 2 | Low | B cells naive | 0.06243730 |
| 3 | Low | B cells naive | 0.13569893 |
| 4 | Low | B cells naive | 0.11419116 |
| 5 | Low | B cells naive | 0.08215979 |
| 6 | High | B cells naive | 0.06081735 |

In [874]:

```
options(repr.plot.width = 20, repr.plot.height = 10)
cols <- unlist(PAIRWISE_COLORS)
names(cols) <- c('High', 'Low')
ggplot(kk, aes(x = Label, y = Frac, fill = label)) + geom_boxplot(outlier.shape = NA) + stat_compare_means(label = "p.signif") + theme_classic(base_size = 20)  + theme(axis.text.x = element_text(angle = 45, vjust = 1, hjust=1), legend.position="top") + xlab('') + ylab('Relative fraction') + scale_fill_manual(values = cols)
ggsave(file.path(out.figs.dir, 'cibersort.frac.pdf'), width = 12, height = 7)
```

In [374]:

```
colnames(data)[c(1, 4, 15,16,19)]
```

1. 'B cells naive'
2. 'T cells CD4 memory resting'
3. 'LINC00987'
4. 'LINC00398'
5. 'ZNF888'

In [495]:

```
data <- res.cib[, c(1, 3, 4, 6,7,8,9,12,13,15,16,19)] %>% scale %>% as.data.frame
```

In [510]:

```
set.seed(1234)   
data <- FetchData(tcga.obj.sub, vars ='LINC00667') %>% {log2(1 + .)} %>% scale %>%  as.data.frame

#data <- cbind.data.frame(data, data.bak)
data$label <- Idents(tcga.obj.sub)
ind <- sample(2, nrow(data), replace = T, prob = c(0.7, 0.3))
ind <- ifelse(ind == 2, 0, 1)
train_data <- data[ind==1, ] #训练数据集
test_data <- data[ind==0, ] #测试数据集
```

In [512]:

```
svm_model <- svm(formula=label ~ ., data = train_data, cross = 10, scale = T) #十折交叉验证
svm_pred <- predict(object = svm_model, newdata = test_data, type = "class", kernel="linear")
svm_model$accuracies
cor.rat <- sum(svm_pred == test_data$label)/nrow(test_data)
print(paste0("该SVM模型下测试集预测准确率为",cor.rat,'.'))
```

1. 41.025641025641
2. 48.7179487179487
3. 39.7435897435897
4. 39.7435897435897
5. 41.025641025641
6. 47.4358974358974
7. 52.5641025641026
8. 42.3076923076923
9. 32.0512820512821
10. 40.5063291139241

```
[1] "该SVM模型下测试集预测准确率为0.445121951219512."
```

In [509]:

```
saveRDS(hub.genes.sub, file = 'hub.genes.sub')
```

### PAN-cancer analysis¶

In [526]:

```
model.res <- lapply(list.files('../0.data/Pan.cancer', pattern = 'TCGA-', full.names  = T), function(fil) {
    obj <- readRDS(fil)
    sub.clinical.tmp <- obj@meta.data[, c('patient', 'vital_status', 'days_to_death', 'days_to_last_follow_up')]
    sub.clinical.tmp$Sur <- sub.clinical.tmp$vital_status == "Dead"
    sub.clinical.tmp$Time <- ifelse(sub.clinical.tmp$Sur, sub.clinical.tmp$days_to_death, sub.clinical.tmp$days_to_last_follow_up)

    expr.tmp <- FetchData(obj, vars = gsub('_', '-', hub.genes.sub))  %>% { log2(1 + .)}
    coefs <- mult.var.cox$coefficients[!is.na(mult.var.cox$coefficients)]
    select.sets <- names(coefs)
    risk.ss <- coefs %*% t(as.matrix(expr.tmp[, gsub('_', '-', select.sets)]))
    sub.clinical.tmp$Groups <- as.vector(risk.ss) 

    formula.fmt <- as.formula(paste0('Surv(Time, Sur)~', 'Groups'))

    tmp.cox <- OS.analysis(sub.clinical.tmp, c('#8A0E1A', '#3574CE'), T, cutoff = T)
    sub.clinical.tmp[, 'Groups'] <- sub.clinical.tmp[, 'Groups'] > tmp.cox$cut.off
    uni.cox <- coxph(formula.fmt, data = sub.clinical.tmp)
    return(list(model = uni.cox, score = risk.ss))
})
```

In [527]:

```
cancers <- gsub('.*TCGA-|-Obj.rds', '', list.files('../0.data/Pan.cancer', pattern = 'TCGA-', full.names  = T))
```

In [548]:

```
model.lst <- lapply(model.res, function(x) x$model)
model.score <- lapply(1 : length(cancers), function(x) { 
    score <- model.res[[x]]$score
    label.cancer <- rep(cancers[x], dim(score)[2])
    return(cbind.data.frame(Score = as.vector(score), Label = label.cancer))
}) %>% do.call(rbind, .)
```

In [549]:

```
head(model.score)
```

A data.frame: 6 × 2

|  | Score | Label |
| --- | --- | --- |
|  | <dbl> | <chr> |
| 1 | -1.38027325 | ACC |
| 2 | -0.96964600 | ACC |
| 3 | -0.08312899 | ACC |
| 4 | 0.03971200 | ACC |
| 5 | 1.10204287 | ACC |
| 6 | 0.96298532 | ACC |

In [550]:

```
model.score$Label <- factor(model.score$Label, levels = cancers)
```

In [557]:

```
model.score$Label <- factor(model.score$Label, levels = cancers)
ggplot(model.score, aes(x = Label, y = Score, color = Label)) + geom_boxplot() + scale_color_manual(values = unlist(CANCER_COLORS)) + theme_bw(base_size = 20) + theme(axis.text.x = element_text(angle = 45, vjust = 1, hjust=1), legend.position="none") + ylim(-5, 5) + xlab('') + ylab('LincSI')
```

In [558]:

```
names(model.lst) <- gsub('.*TCGA-|-Obj.rds', '', list.files('../0.data/Pan.cancer', pattern = 'TCGA-', full.names  = T))
```

In [559]:

```
model.lst
```

```
$ACC
Call:
coxph(formula = formula.fmt, data = sub.clinical.tmp)

             coef exp(coef) se(coef)     z       p
GroupsTRUE 1.4708    4.3528   0.4968 2.961 0.00307

Likelihood ratio test=11.34  on 1 df, p=0.000757
n= 79, number of events= 28 

$BLCA
Call:
coxph(formula = formula.fmt, data = sub.clinical.tmp)

             coef exp(coef) se(coef)     z        p
GroupsTRUE 0.6151    1.8498   0.1454 4.229 2.34e-05

Likelihood ratio test=17.66  on 1 df, p=2.642e-05
n= 431, number of events= 193 
   (因为不存在，2个观察量被删除了)

$BRCA
Call:
coxph(formula = formula.fmt, data = sub.clinical.tmp)

             coef exp(coef) se(coef)     z      p
GroupsTRUE 1.7315    5.6490   0.1516 11.42 <2e-16

Likelihood ratio test=106.3  on 1 df, p=< 2.2e-16
n= 1220, number of events= 200 
   (因为不存在，2个观察量被删除了)

$CESC
Call:
coxph(formula = formula.fmt, data = sub.clinical.tmp)

             coef exp(coef) se(coef)     z      p
GroupsTRUE 0.4492    1.5671   0.2358 1.905 0.0568

Likelihood ratio test=3.69  on 1 df, p=0.05484
n= 309, number of events= 74 

$CHOL
Call:
coxph(formula = formula.fmt, data = sub.clinical.tmp)

                coef exp(coef)  se(coef)     z     p
GroupsTRUE 1.822e+01 8.153e+07 5.277e+03 0.003 0.997

Likelihood ratio test=5.47  on 1 df, p=0.01939
n= 45, number of events= 23 

$COAD
Call:
coxph(formula = formula.fmt, data = sub.clinical.tmp)

             coef exp(coef) se(coef)     z       p
GroupsTRUE 0.6493    1.9142   0.2106 3.083 0.00205

Likelihood ratio test=8.53  on 1 df, p=0.003495
n= 518, number of events= 115 
   (因为不存在，3个观察量被删除了)

$DLBC
Call:
coxph(formula = formula.fmt, data = sub.clinical.tmp)

              coef exp(coef) se(coef)     z     p
GroupsTRUE -1.3812    0.2513   0.8689 -1.59 0.112

Likelihood ratio test=2.07  on 1 df, p=0.1502
n= 48, number of events= 9 

$ESCA
Call:
coxph(formula = formula.fmt, data = sub.clinical.tmp)

             coef exp(coef) se(coef)     z       p
GroupsTRUE 0.7429    2.1019   0.2555 2.908 0.00364

Likelihood ratio test=7.97  on 1 df, p=0.004747
n= 173, number of events= 68 

$GBM
Call:
coxph(formula = formula.fmt, data = sub.clinical.tmp)

             coef exp(coef) se(coef)     z      p
GroupsTRUE 0.4682    1.5972   0.1981 2.364 0.0181

Likelihood ratio test=5.95  on 1 df, p=0.01474
n= 168, number of events= 136 
   (因为不存在，6个观察量被删除了)

$HNSC
Call:
coxph(formula = formula.fmt, data = sub.clinical.tmp)

             coef exp(coef) se(coef)    z       p
GroupsTRUE 0.5148    1.6734   0.1570 3.28 0.00104

Likelihood ratio test=9.72  on 1 df, p=0.001819
n= 543, number of events= 250 
   (因为不存在，3个观察量被删除了)

$KICH
Call:
coxph(formula = formula.fmt, data = sub.clinical.tmp)

             coef exp(coef) se(coef)     z      p
GroupsTRUE 1.1376    3.1194   0.6685 1.702 0.0888

Likelihood ratio test=3.36  on 1 df, p=0.06699
n= 87, number of events= 12 
   (因为不存在，2个观察量被删除了)

$KIRC
Call:
coxph(formula = formula.fmt, data = sub.clinical.tmp)

              coef exp(coef) se(coef)      z      p
GroupsTRUE -0.3622    0.6961   0.1559 -2.324 0.0201

Likelihood ratio test=5.66  on 1 df, p=0.01738
n= 611, number of events= 200 

$KIRP
Call:
coxph(formula = formula.fmt, data = sub.clinical.tmp)

             coef exp(coef) se(coef)     z        p
GroupsTRUE 1.0060    2.7345   0.3033 3.316 0.000912

Likelihood ratio test=9.49  on 1 df, p=0.002066
n= 320, number of events= 51 
   (因为不存在，1个观察量被删除了)

$LAML
Call:
coxph(formula = formula.fmt, data = sub.clinical.tmp)

              coef exp(coef) se(coef)      z     p
GroupsTRUE -0.3489    0.7055   0.2158 -1.616 0.106

Likelihood ratio test=2.58  on 1 df, p=0.108
n= 140, number of events= 87 
   (因为不存在，11个观察量被删除了)

$LGG
Call:
coxph(formula = formula.fmt, data = sub.clinical.tmp)

             coef exp(coef) se(coef)     z      p
GroupsTRUE 0.4707    1.6012   0.1902 2.475 0.0133

Likelihood ratio test=5.7  on 1 df, p=0.01696
n= 527, number of events= 136 
   (因为不存在，2个观察量被删除了)

$LIHC
Call:
coxph(formula = formula.fmt, data = sub.clinical.tmp)

             coef exp(coef) se(coef)     z        p
GroupsTRUE 0.7123    2.0386   0.2028 3.512 0.000444

Likelihood ratio test=10.59  on 1 df, p=0.001139
n= 423, number of events= 164 
   (因为不存在，1个观察量被删除了)

$LUAD
Call:
coxph(formula = formula.fmt, data = sub.clinical.tmp)

              coef exp(coef) se(coef)      z      p
GroupsTRUE -0.2661    0.7664   0.1617 -1.645 0.0999

Likelihood ratio test=2.83  on 1 df, p=0.09234
n= 585, number of events= 214 
   (因为不存在，9个观察量被删除了)

$LUSC
Call:
coxph(formula = formula.fmt, data = sub.clinical.tmp)

             coef exp(coef) se(coef)     z      p
GroupsTRUE 0.3325    1.3944   0.1299 2.559 0.0105

Likelihood ratio test=6.45  on 1 df, p=0.01111
n= 545, number of events= 242 
   (因为不存在，6个观察量被删除了)

$MESO
Call:
coxph(formula = formula.fmt, data = sub.clinical.tmp)

             coef exp(coef) se(coef)     z        p
GroupsTRUE 0.9052    2.4725   0.2544 3.559 0.000373

Likelihood ratio test=13.36  on 1 df, p=0.0002566
n= 85, number of events= 72 
   (因为不存在，1个观察量被删除了)

$OV
Call:
coxph(formula = formula.fmt, data = sub.clinical.tmp)

             coef exp(coef) se(coef)     z      p
GroupsTRUE 0.3184    1.3749   0.1654 1.925 0.0542

Likelihood ratio test=3.94  on 1 df, p=0.04712
n= 377, number of events= 231 
   (因为不存在，2个观察量被删除了)

$PAAD
Call:
coxph(formula = formula.fmt, data = sub.clinical.tmp)

             coef exp(coef) se(coef)     z      p
GroupsTRUE 0.5428    1.7209   0.2182 2.487 0.0129

Likelihood ratio test=6.45  on 1 df, p=0.01111
n= 182, number of events= 94 

$PCPG
Call:
coxph(formula = formula.fmt, data = sub.clinical.tmp)

              coef exp(coef) se(coef)      z      p
GroupsTRUE -2.2493    0.1055   1.0694 -2.103 0.0354

Likelihood ratio test=7.06  on 1 df, p=0.007904
n= 186, number of events= 8 

$PRAD
Call:
coxph(formula = formula.fmt, data = sub.clinical.tmp)

             coef exp(coef) se(coef)     z     p
GroupsTRUE 0.9452    2.5732   0.7927 1.192 0.233

Likelihood ratio test=1.68  on 1 df, p=0.1951
n= 551, number of events= 10 

$READ
Call:
coxph(formula = formula.fmt, data = sub.clinical.tmp)

              coef exp(coef) se(coef)      z      p
GroupsTRUE -0.9100    0.4025   0.3774 -2.411 0.0159

Likelihood ratio test=5.81  on 1 df, p=0.01597
n= 175, number of events= 30 
   (因为不存在，2个观察量被删除了)

$SARC
Call:
coxph(formula = formula.fmt, data = sub.clinical.tmp)

             coef exp(coef) se(coef)     z       p
GroupsTRUE 0.8430    2.3233   0.2637 3.197 0.00139

Likelihood ratio test=8.57  on 1 df, p=0.003424
n= 265, number of events= 99 

$SKCM
Call:
coxph(formula = formula.fmt, data = sub.clinical.tmp)

             coef exp(coef) se(coef)     z        p
GroupsTRUE 0.6948    2.0032   0.1929 3.601 0.000316

Likelihood ratio test=11.05  on 1 df, p=0.0008865
n= 463, number of events= 222 
   (因为不存在，9个观察量被删除了)

$STAD
Call:
coxph(formula = formula.fmt, data = sub.clinical.tmp)

             coef exp(coef) se(coef)     z     p
GroupsTRUE 0.3087    1.3616   0.2157 1.431 0.152

Likelihood ratio test=2.19  on 1 df, p=0.139
n= 399, number of events= 153 
   (因为不存在，8个观察量被删除了)

$TGCT
Call:
coxph(formula = formula.fmt, data = sub.clinical.tmp)

            coef exp(coef) se(coef)    z     p
GroupsTRUE 1.579     4.851    1.161 1.36 0.174

Likelihood ratio test=2.2  on 1 df, p=0.1379
n= 139, number of events= 4 
   (因为不存在，17个观察量被删除了)

$THCA
Call:
coxph(formula = formula.fmt, data = sub.clinical.tmp)

              coef exp(coef) se(coef)      z      p
GroupsTRUE -1.0858    0.3376   0.5594 -1.941 0.0523

Likelihood ratio test=4.54  on 1 df, p=0.03304
n= 568, number of events= 20 

$THYM
Call:
coxph(formula = formula.fmt, data = sub.clinical.tmp)

             coef exp(coef) se(coef)     z     p
GroupsTRUE 1.1413    3.1308   0.8046 1.418 0.156

Likelihood ratio test=2.39  on 1 df, p=0.1221
n= 120, number of events= 9 
   (因为不存在，1个观察量被删除了)

$UCEC
Call:
coxph(formula = formula.fmt, data = sub.clinical.tmp)

             coef exp(coef) se(coef)     z      p
GroupsTRUE 0.5091    1.6637   0.2091 2.434 0.0149

Likelihood ratio test=5.64  on 1 df, p=0.01754
n= 574, number of events= 97 
   (因为不存在，13个观察量被删除了)

$UCS
Call:
coxph(formula = formula.fmt, data = sub.clinical.tmp)

             coef exp(coef) se(coef)     z      p
GroupsTRUE 0.9138    2.4939   0.4401 2.076 0.0379

Likelihood ratio test=5  on 1 df, p=0.02536
n= 56, number of events= 34 

$UVM
Call:
coxph(formula = formula.fmt, data = sub.clinical.tmp)

             coef exp(coef) se(coef)     z       p
GroupsTRUE 2.0154    7.5038   0.6344 3.177 0.00149

Likelihood ratio test=14.94  on 1 df, p=0.0001109
n= 80, number of events= 23
```

In [561]:

```
univ.results <- lapply(model.lst, function(x){
    x <- summary(x)
    p.value <- signif(x$wald["pvalue"], digits = 2)
    HR <-signif(x$coef[2], digits = 2)
    lower.95 <- signif(x$conf.int[,"lower .95"], 2)
    upper.95 <- signif(x$conf.int[,"upper .95"], 2)
    conf <- paste0(HR, " (", lower.95, "-", upper.95, ")")
    res <- c(HR, lower.95, upper.95, conf, p.value)
    return(res)
}) %>% as.data.frame %>% t

rownames(univ.results) <- names(model.lst)

colnames(univ.results) <- c('HR', 'lower.95', 'upper.95', 'conf', 'p.value')
plot.res <- cbind.data.frame(charac = rownames(univ.results), univ.results)
plot.res[plot.res == Inf] <- 9999
plot.res[, 2] <- as.numeric(plot.res[, 2]) %>% { log2(1 + .) }
plot.res[, 3] <- as.numeric(plot.res[, 3]) %>% { log2(1 + .) }
plot.res[, 4] <- as.numeric(plot.res[, 4]) %>% { log2(1 + .) }
plot.res[, 6] <- as.numeric(plot.res[, 6])
plot.res <- plot.res[order(plot.res$p.value), ]
plot.res['TGCT', 'upper.95'] <- 1
plot.res[plot.res == Inf] <- 999

pdf(file.path(out.figs.dir, 'pan.cancer.hub.genes.forestplot.pdf'), width = 8, height = 10)
forestplot(
    plot.res[, c(1, 5, 6)],
    mean = plot.res[, 2],
    lower = plot.res[, 3],
    upper = plot.res[, 4],
    zero = 1,
    col=fpColors(box="darkblue", lines="black", zero = "gray50"),
    boxsize = 0.3,
    graph.pos = 2
)
dev.off()
```

**png:** 2

In [761]:

```
head(univ.results)
```

A matrix: 6 × 5 of type chr

|  | HR | lower.95 | upper.95 | conf | p.value |
| --- | --- | --- | --- | --- | --- |
| ACC | 4.4 | 1.6 | 12 | 4.4 (1.6-12) | 0.0031 |
| BLCA | 1.8 | 1.4 | 2.5 | 1.8 (1.4-2.5) | 2.3e-05 |
| BRCA | 5.6 | 4.2 | 7.6 | 5.6 (4.2-7.6) | 3.4e-30 |
| CESC | 1.6 | 0.99 | 2.5 | 1.6 (0.99-2.5) | 0.057 |
| CHOL | 8.2e+07 | 0 | Inf | 8.2e+07 (0-Inf) | 1 |
| COAD | 1.9 | 1.3 | 2.9 | 1.9 (1.3-2.9) | 0.0021 |

In [564]:

```
grid.table(univ.results)
```

In [762]:

```
univ.results %>% head
```

A matrix: 6 × 5 of type chr

|  | HR | lower.95 | upper.95 | conf | p.value |
| --- | --- | --- | --- | --- | --- |
| ACC | 4.4 | 1.6 | 12 | 4.4 (1.6-12) | 0.0031 |
| BLCA | 1.8 | 1.4 | 2.5 | 1.8 (1.4-2.5) | 2.3e-05 |
| BRCA | 5.6 | 4.2 | 7.6 | 5.6 (4.2-7.6) | 3.4e-30 |
| CESC | 1.6 | 0.99 | 2.5 | 1.6 (0.99-2.5) | 0.057 |
| CHOL | 8.2e+07 | 0 | Inf | 8.2e+07 (0-Inf) | 1 |
| COAD | 1.9 | 1.3 | 2.9 | 1.9 (1.3-2.9) | 0.0021 |

In [764]:

```
subset(as.data.frame(univ.results), p.value <= 0.01)
```

A data.frame: 10 × 5

|  | HR | lower.95 | upper.95 | conf | p.value |
| --- | --- | --- | --- | --- | --- |
|  | <chr> | <chr> | <chr> | <chr> | <chr> |
| ACC | 4.4 | 1.6 | 12 | 4.4 (1.6-12) | 0.0031 |
| COAD | 1.9 | 1.3 | 2.9 | 1.9 (1.3-2.9) | 0.0021 |
| ESCA | 2.1 | 1.3 | 3.5 | 2.1 (1.3-3.5) | 0.0036 |
| HNSC | 1.7 | 1.2 | 2.3 | 1.7 (1.2-2.3) | 0.001 |
| KIRP | 2.7 | 1.5 | 5 | 2.7 (1.5-5) | 0.00091 |
| LIHC | 2 | 1.4 | 3 | 2 (1.4-3) | 0.00044 |
| MESO | 2.5 | 1.5 | 4.1 | 2.5 (1.5-4.1) | 0.00037 |
| SARC | 2.3 | 1.4 | 3.9 | 2.3 (1.4-3.9) | 0.0014 |
| SKCM | 2 | 1.4 | 2.9 | 2 (1.4-2.9) | 0.00032 |
| UVM | 7.5 | 2.2 | 26 | 7.5 (2.2-26) | 0.0015 |

In [772]:

```
univ.results <- as.data.frame(univ.results)
univ.results[, 'p.value'] <- as.numeric(univ.results[, 'p.value'])
```

In [777]:

```
sig.cancers <- subset(as.data.frame(univ.results), p.value <= 0.01) %>% rownames
sig.cancers <- setdiff(sig.cancers, 'BRCA')
```

In [803]:

```
options(repr.plot.width = 10, repr.plot.height = 10)
cox.res <- lapply(sig.cancers, function(cancer) {
    obj <- readRDS(sprintf('../0.data/Pan.cancer/TCGA-%s-Obj.rds', cancer))
    obj.sub <- subset(obj, sample_type == 'Primary Tumor' | sample_type == 'Metastatic' | sample_type == 'Primary Blood Derived Cancer - Peripheral Blood')
    sub.clinical.tmp <- obj.sub@meta.data[, c('patient', 'vital_status', 'days_to_death', 'days_to_last_follow_up')]
    sub.clinical.tmp$Sur <- sub.clinical.tmp$vital_status == "Dead"
    sub.clinical.tmp$Time <- ifelse(sub.clinical.tmp$Sur, sub.clinical.tmp$days_to_death, sub.clinical.tmp$days_to_last_follow_up) / 365

    expr.tmp <- FetchData(obj.sub, vars = gsub('_', '-', hub.genes.sub))  %>% { log2(1 + .)}
    coefs <- mult.var.cox$coefficients[!is.na(mult.var.cox$coefficients)]
    select.sets <- names(coefs)
    risk.ss <- coefs %*% t(as.matrix(expr.tmp[, gsub('_', '-', select.sets)]))
    sub.clinical.tmp$Groups <- as.vector(risk.ss) 
    tmp.cox <- OS.analysis(sub.clinical.tmp, c('#8A0E1A', '#3574CE'), F, cutoff = T, prefix = sprintf('TCGA_%s_OS.pdf', cancer), risk.table = F, title = cancer)
})
```

In [804]:

```
sur.plot <- lapply(cox.res, function(x) x$gplot)
length(sur.plot)
```

11

In [806]:

```
options(repr.plot.width = 10, repr.plot.height = 20)
res.plot <-arrange_ggsurvplots(sur.plot, print = FALSE, ncol = 3, nrow = 4, risk.table.height = 0)
ggsave(file.path(out.figs.dir, 'Pan.cancer.sig.pdf'), plot = res.plot, width = 10, height = 13)
res.plot
```

```
[[1]]
NULL
```

In [781]:

```
library(estimate)
esti.res <- lapply(sig.cancers, function(cancer) {
    obj <- readRDS(sprintf('../0.data/Pan.cancer/TCGA-%s-Obj.rds', cancer))
    obj.sub <- subset(obj, sample_type == 'Primary Tumor' | sample_type == 'Metastatic' | sample_type == 'Primary Blood Derived Cancer - Peripheral Blood')
    tmp.xls <- file.path(out.data.dir, 'tmp_training_data.xls')
    tmp.gct <- file.path(out.data.dir, 'tmp_training_data.gct')
    tmp.score <- file.path(out.data.dir, 'tmp_immune_scores.gct')

    write.table(GetAssayData(obj.sub) %>% as.matrix, tmp.xls, sep = '\t', row.names = TRUE, col.names = NA, quote = FALSE)
    filterCommonGenes(tmp.xls, output.f = tmp.gct, id = "GeneSymbol")
    estimateScore(tmp.gct, tmp.score, platform  = 'affymetrix')
    purity.score <- read.table(tmp.score, sep = '\t', header = TRUE, row.names = 1, skip = 2) %>% .[, -1] 
})
```

```
[1] "Merged dataset includes 10214 genes (198 mismatched)."
[1] "1 gene set: StromalSignature  overlap= 139"
[1] "2 gene set: ImmuneSignature  overlap= 141"
[1] "Merged dataset includes 10214 genes (198 mismatched)."
[1] "1 gene set: StromalSignature  overlap= 139"
[1] "2 gene set: ImmuneSignature  overlap= 141"
[1] "Merged dataset includes 10214 genes (198 mismatched)."
[1] "1 gene set: StromalSignature  overlap= 139"
[1] "2 gene set: ImmuneSignature  overlap= 141"
[1] "Merged dataset includes 10214 genes (198 mismatched)."
[1] "1 gene set: StromalSignature  overlap= 139"
[1] "2 gene set: ImmuneSignature  overlap= 141"
[1] "Merged dataset includes 10214 genes (198 mismatched)."
[1] "1 gene set: StromalSignature  overlap= 139"
[1] "2 gene set: ImmuneSignature  overlap= 141"
[1] "Merged dataset includes 10214 genes (198 mismatched)."
[1] "1 gene set: StromalSignature  overlap= 139"
[1] "2 gene set: ImmuneSignature  overlap= 141"
[1] "Merged dataset includes 10214 genes (198 mismatched)."
[1] "1 gene set: StromalSignature  overlap= 139"
[1] "2 gene set: ImmuneSignature  overlap= 141"
[1] "Merged dataset includes 10214 genes (198 mismatched)."
[1] "1 gene set: StromalSignature  overlap= 139"
[1] "2 gene set: ImmuneSignature  overlap= 141"
[1] "Merged dataset includes 10214 genes (198 mismatched)."
[1] "1 gene set: StromalSignature  overlap= 139"
[1] "2 gene set: ImmuneSignature  overlap= 141"
[1] "Merged dataset includes 10214 genes (198 mismatched)."
[1] "1 gene set: StromalSignature  overlap= 139"
[1] "2 gene set: ImmuneSignature  overlap= 141"
[1] "Merged dataset includes 10214 genes (198 mismatched)."
[1] "1 gene set: StromalSignature  overlap= 139"
[1] "2 gene set: ImmuneSignature  overlap= 141"
```

In [782]:

```
names(esti.res) <- sig.cancers
```

In [783]:

```
risk.ss <- lapply(sig.cancers, function(cancer) {
    obj <- readRDS(sprintf('../0.data/Pan.cancer/TCGA-%s-Obj.rds', cancer))
    obj.sub <- subset(obj, sample_type == 'Primary Tumor' | sample_type == 'Metastatic' | sample_type == 'Primary Blood Derived Cancer - Peripheral Blood')
    sub.clinical.tmp <- obj.sub@meta.data[, c('patient', 'vital_status', 'days_to_death', 'days_to_last_follow_up')]
    sub.clinical.tmp$Sur <- sub.clinical.tmp$vital_status == "Dead"
    sub.clinical.tmp$Time <- ifelse(sub.clinical.tmp$Sur, sub.clinical.tmp$days_to_death, sub.clinical.tmp$days_to_last_follow_up)

    expr.tmp <- FetchData(obj.sub, vars = gsub('_', '-', hub.genes.sub))  %>% { log2(1 + .)}
    coefs <- mult.var.cox$coefficients[!is.na(mult.var.cox$coefficients)]
    select.sets <- names(coefs)
    risk.ss <- coefs %*% t(as.matrix(expr.tmp[, gsub('_', '-', select.sets)]))
    return(as.vector(risk.ss))
})
```

In [784]:

```
names(risk.ss) <- sig.cancers
```

In [115]:

```
sig.cancers <- c('ACC','BLCA','COAD','ESCA','HNSC','KIRP','LIHC','MESO','SARC','SKCM','UVM')
```

In [792]:

```
est.plot <- lapply(1 : length(sig.cancers), function(x) {
    group <- cox.res[[x]]$group
    cbind.data.frame(esti.res[[x]][c(1, 2), ] %>% t %>% scale, Group = group, Cancer = sig.cancers[x])    
}) %>% do.call(rbind, .)
```

In [793]:

```
sca.df <- cbind.data.frame(Risk = risk.ss$UVM, Immune = esti.res[['UVM']] %>% .[2, ] %>% t %>% scale) %>% as.data.frame
```

In [794]:

```
options(repr.plot.width = 6, repr.plot.height = 6)
ggplot(data = sca.df, aes(x = Risk, y = ImmuneScore)) + geom_point(size = 3, color = 'blue', alpha = 0.5) + geom_smooth(method = "lm", se = T, color = 'red', lwd = 1.5) + stat_cor(method="pearson") + theme_classic(base_size = 20) + theme(legend.position = 'none') + xlab('Risk score') + ylab('Immune score')
ggsave(file.path(out.figs.dir, 'UVM.risk.immune.pdf'), width = 6, height = 6)
```

```
`geom_smooth()` using formula 'y ~ x'

`geom_smooth()` using formula 'y ~ x'
```

In [795]:

```
head(est.plot)
```

A data.frame: 6 × 4

|  | StromalScore | ImmuneScore | Group | Cancer |
| --- | --- | --- | --- | --- |
|  | <dbl> | <dbl> | <chr> | <chr> |
| TCGA.OR.A5JJ.01A.11R.A29S.07 | -0.6676613 | -0.7090082 | Low risk | ACC |
| TCGA.OR.A5LT.01A.11R.A29S.07 | -0.7557121 | -0.7241064 | Low risk | ACC |
| TCGA.OR.A5LH.01A.11R.A29S.07 | -0.6019411 | 0.1862074 | High risk | ACC |
| TCGA.OR.A5K0.01A.11R.A29S.07 | -1.2030903 | -0.6718270 | High risk | ACC |
| TCGA.OR.A5K9.01A.11R.A29S.07 | -0.3573127 | -0.8941908 | High risk | ACC |
| TCGA.OR.A5LD.01A.11R.A29S.07 | -0.7742672 | -1.1061682 | High risk | ACC |

In [796]:

```
est.plot <- tidyr::gather(est.plot, 'Label', 'Score', -Group, -Cancer)
head(est.plot)
```

A data.frame: 6 × 4

|  | Group | Cancer | Label | Score |
| --- | --- | --- | --- | --- |
|  | <chr> | <chr> | <chr> | <dbl> |
| 1 | Low risk | ACC | StromalScore | -0.6676613 |
| 2 | Low risk | ACC | StromalScore | -0.7557121 |
| 3 | High risk | ACC | StromalScore | -0.6019411 |
| 4 | High risk | ACC | StromalScore | -1.2030903 |
| 5 | High risk | ACC | StromalScore | -0.3573127 |
| 6 | High risk | ACC | StromalScore | -0.7742672 |

In [797]:

```
options(repr.plot.width = 15, repr.plot.height = 6)
est.plot.stromal <- subset(est.plot, Label == 'ImmuneScore')
cols <- unlist(PAIRWISE_COLORS)
names(cols) <- c('High risk', 'Low risk')
ggplot(data = est.plot.stromal, aes(x = Cancer, y = Score, fill = Group)) + geom_boxplot(outlier.shape = NA) + theme_bw(base_size = 20) + stat_compare_means(method = 't.test', label = "p.signif") + theme_classic(base_size = 20)  + theme(axis.text.x = element_text(angle = 45, vjust = 1, hjust=1), legend.position="top") + xlab('')  + ylab('Immune Score') + scale_fill_manual(values = cols)
ggsave(file.path(out.figs.dir, 'Immune.score.pan.cancer.pdf'), width = 8, height = 6)
```

In [798]:

```
options(repr.plot.width = 15, repr.plot.height = 6)
est.plot.stromal <- subset(est.plot, Label == 'StromalScore')
ggplot(data = est.plot.stromal, aes(x = Cancer, y = Score, fill = Group)) + geom_boxplot(outlier.shape = NA) + theme_bw(base_size = 20) + stat_compare_means(method = 't.test', label = "p.signif") + theme_classic(base_size = 20)  + theme(axis.text.x = element_text(angle = 45, vjust = 1, hjust=1), legend.position="top") + xlab('')  + ylab('Stromal Score')
```

In [799]:

```
ips.ss <- lapply(sig.cancers, function(cancer) {
    obj <- readRDS(sprintf('../0.data/Pan.cancer/TCGA-%s-Obj.rds', cancer))
    obj.sub <- subset(obj, sample_type == 'Primary Tumor' | sample_type == 'Metastatic' | sample_type == 'Primary Blood Derived Cancer - Peripheral Blood')
    source('IPS.R')
    ips.score <- IPSCore(GetAssayData(obj.sub) %>% as.data.frame %>% { log2(1 + .) })
})
```

```
differently named or missing genes:  CCL3L1 
differently named or missing genes:  CCL3L1 
differently named or missing genes:  CCL3L1 
differently named or missing genes:  CCL3L1 
differently named or missing genes:  CCL3L1 
differently named or missing genes:  CCL3L1 
differently named or missing genes:  CCL3L1 
differently named or missing genes:  CCL3L1 
differently named or missing genes:  CCL3L1 
differently named or missing genes:  CCL3L1 
differently named or missing genes:  CCL3L1
```

In [800]:

```
est.plot <- lapply(1 : length(sig.cancers), function(x) {   
    group <- cox.res[[x]]$group
    dim(group)
    dim(ips.ss[[x]])
    cbind.data.frame(IPS = ips.ss[[x]][, 6], Group = group, Cancer = sig.cancers[x])  
}) %>% do.call(rbind, .)
```

In [801]:

```
head(est.plot)
```

A data.frame: 6 × 3

|  | IPS | Group | Cancer |
| --- | --- | --- | --- |
|  | <dbl> | <chr> | <chr> |
| 1 | 3.396132 | Low risk | ACC |
| 2 | 3.668195 | Low risk | ACC |
| 3 | 4.451541 | High risk | ACC |
| 4 | 4.348812 | High risk | ACC |
| 5 | 3.005572 | High risk | ACC |
| 6 | 2.390665 | High risk | ACC |

In [802]:

```
options(repr.plot.width = 15, repr.plot.height = 6)
ggplot(data = est.plot, aes(x = Cancer, y = IPS, fill = Group)) + geom_boxplot(outlier.shape = NA) + theme_bw(base_size = 20) + stat_compare_means(label = "p.signif") + theme_classic(base_size = 20)  + theme(axis.text.x = element_text(angle = 45, vjust = 1, hjust=1), legend.position="top") + xlab('')
```

In [116]:

```
log2FcCalc <- lapply(sig.cancers, function(cancer) {    
    obj <- readRDS(sprintf('../0.data/Pan.cancer/TCGA-%s-Obj.rds', cancer))
    if (!'Solid Tissue Normal' %in% (obj@meta.data$sample_type %>% unique)) return(NA)
    obj.tumor <- subset(obj, sample_type == 'Primary Tumor' | sample_type == 'Metastatic' | sample_type == 'Primary Blood Derived Cancer - Peripheral Blood')
    obj.normal <- subset(obj, sample_type == 'Solid Tissue Normal')
    tumor.exprs <- colMeans(FetchData(obj.tumor, vars = gsub('_', '-', hub.genes.sub)))
    normal.exprs <- colMeans(FetchData(obj.normal, vars = gsub('_', '-', hub.genes.sub)))
    log2(tumor.exprs / (0.01 + normal.exprs))
})
```

In [117]:

```
names(log2FcCalc) <- sig.cancers
log2FcCalc <- log2FcCalc[!is.na(log2FcCalc)]
log2FcCalc <- log2FcCalc %>% as.data.frame
log2FcCalc
```

In [121]:

```
options(repr.plot.width = 20, repr.plot.height = 4)

pheatmap::pheatmap(t(log2FcCalc), cluster_rows = T, cluster_cols = T, fontsize = 15, file = file.path(out.figs.dir, 'log2fc.heatmap.pdf'), height = 5, width = 12)
```

In [122]:

```
pheatmap::pheatmap(t(log2FcCalc), cluster_rows = F, cluster_cols = F, fontsize = 15)
```

In [157]:

```
log2FcCalc <- lapply(sig.cancers, function(cancer) {
    obj <- readRDS(sprintf('../0.data/Pan.cancer/TCGA-%s-Obj.rds', cancer))
    obj.sub <- subset(obj, sample_type == 'Primary Tumor' | sample_type == 'Metastatic' | sample_type == 'Primary Blood Derived Cancer - Peripheral Blood')
    sub.clinical.tmp <- obj.sub@meta.data[, c('patient', 'vital_status', 'days_to_death', 'days_to_last_follow_up')]
    sub.clinical.tmp$Sur <- sub.clinical.tmp$vital_status == "Dead"
    sub.clinical.tmp$Time <- ifelse(sub.clinical.tmp$Sur, sub.clinical.tmp$days_to_death, sub.clinical.tmp$days_to_last_follow_up) / 365

    expr.tmp <- FetchData(obj.sub, vars = gsub('_', '-', hub.genes.sub))  %>% { log2(1 + .)}
    coefs <- mult.var.cox$coefficients[!is.na(mult.var.cox$coefficients)]
    select.sets <- names(coefs)
    risk.ss <- coefs %*% t(as.matrix(expr.tmp[, gsub('_', '-', select.sets)]))
    sub.clinical.tmp$Groups <- as.vector(risk.ss) 
    tmp.cox <- OS.analysis(sub.clinical.tmp, c('#8A0E1A', '#3574CE'), T, cutoff = T, prefix = sprintf('TCGA_%s_OS.pdf', cancer), risk.table = F, title = cancer, out.figs.dir = out.figs.dir)
    obj.sub@meta.data$Group <- tmp.cox$group
    obj.sub.high <- subset(obj.sub, Group == 'High risk')
    obj.sub.low <- subset(obj.sub, Group == 'Low risk')
        
    tumor.exprs <- colMeans(FetchData(obj.sub.high, vars = gsub('_', '-', hub.genes.sub)))
    normal.exprs <- colMeans(FetchData(obj.sub.low, vars = gsub('_', '-', hub.genes.sub)))
    log2(tumor.exprs / (0.01 + normal.exprs))   
})
```

In [158]:

```
names(log2FcCalc) <- sig.cancers
log2FcCalc <- log2FcCalc[!is.na(log2FcCalc)]
log2FcCalc <- log2FcCalc %>% as.data.frame
log2FcCalc
```

A data.frame: 29 × 11

|  | ACC | BLCA | COAD | ESCA | HNSC | KIRP | LIHC | MESO | SARC | SKCM | UVM |
| --- | --- | --- | --- | --- | --- | --- | --- | --- | --- | --- | --- |
|  | <dbl> | <dbl> | <dbl> | <dbl> | <dbl> | <dbl> | <dbl> | <dbl> | <dbl> | <dbl> | <dbl> |
| AF131215.8 | 0.81502490 | 0.985456140 | 0.49670895 | 0.74413468 | 1.060263376 | 0.296494449 | 1.44551522 | 1.55139612 | 1.610029111 | -0.46025186 | -0.78732561 |
| LINC01235 | 0.79352990 | -0.003049091 | 0.54927866 | 0.05226120 | 0.361366930 | 0.601773773 | 0.90354849 | -0.48324568 | 1.237643779 | 0.59361012 | -0.21914152 |
| LINC00987 | -0.59828261 | -0.356178924 | -0.13130242 | -0.71026387 | -0.440232717 | -0.054720303 | -1.07543795 | -0.57135891 | 0.212514625 | -1.94444031 | -1.04731521 |
| LINC00398 | 1.39404324 | -0.802347687 | 0.31738435 | -0.64746742 | -0.224746088 | -0.318581194 | -0.62504077 | 0.16803319 | -0.927741529 | -1.19074168 | -2.29360576 |
| LINC00667 | -0.04149651 | -0.325220721 | -0.36503380 | -0.25645480 | -0.186927729 | -0.067364523 | 0.21649312 | 0.05173750 | 0.002696177 | -0.25451095 | -0.50527619 |
| CTD-2554C21.2 | -1.16212407 | -0.132536966 | 0.24699467 | -0.86162162 | -1.215301061 | -0.064666402 | 1.15549988 | -0.94719916 | -2.145402297 | -1.20350743 | -2.91003799 |
| ZNF888 | -0.46709452 | 1.050699509 | 0.55517740 | 0.52792256 | 0.402550143 | 0.756429445 | 1.70919466 | -0.22053915 | 0.261396645 | -0.28004616 | -0.55602123 |
| LL0XNC01-237H1.2 | -0.67440783 | 0.293321280 | 1.31644833 | 0.30570838 | 0.054336575 | 0.507548258 | 0.87320843 | 0.08276762 | 0.062144602 | -0.18056468 | -0.26072174 |
| AC016995.3 | -0.52141708 | -0.827352354 | -0.07788888 | -0.81841876 | -0.601072708 | -0.164734264 | 0.95660135 | -0.44811498 | -1.280685621 | -1.18136932 | -0.98710883 |
| SFTA1P | -0.01026646 | -0.113540647 | 0.90607415 | 1.61467903 | 2.202017770 | 0.030230840 | 0.60409501 | 2.95277066 | 3.786315185 | 0.57110451 | 1.63574396 |
| AL022344.7 | 1.19618036 | 1.204773330 | 0.17394336 | 0.47583836 | 0.924173999 | 0.882106211 | 1.24935609 | 0.09061006 | 0.840935087 | -0.26052672 | -0.42554710 |
| LINC00958 | -0.76944116 | 0.297475079 | 0.32210672 | -0.05484957 | 0.449080060 | 0.442752872 | 2.55525531 | -0.63679204 | 0.446375262 | 1.63975578 | -1.27048749 |
| LINC00327 | -0.89781931 | -0.882311099 | -0.53162664 | -0.35549254 | -0.454252708 | 0.010934274 | 0.12912970 | 0.42089088 | -0.731990212 | -1.93488488 | -0.13932054 |
| LINC00346 | 0.75562683 | 0.044639528 | 0.71059982 | 0.40194617 | 0.823715437 | 0.063824790 | 1.24492206 | 1.54291241 | 0.977464567 | 0.60611057 | 1.32799800 |
| AC016735.1 | -0.17199013 | -0.407697505 | -0.23277451 | -0.26338300 | 0.005052688 | 0.190627169 | 0.96565685 | -0.02083048 | 0.172430063 | -1.54324128 | -0.91268241 |
| AC009495.2 | -1.22858189 | -0.343907344 | 0.03270092 | -0.17427365 | 0.181089286 | -0.003834396 | 0.31727963 | -1.11317440 | -0.897121842 | -0.97564935 | -0.55432696 |
| AC092431.3 | -0.38962562 | 0.248459553 | 0.94431091 | 0.49368153 | 0.801177825 | 1.199695779 | 0.70125537 | 0.26790788 | 0.023966442 | 0.33672297 | -0.31887125 |
| CTC-498J12.1 | -1.32861937 | -0.163256781 | -0.34351687 | -0.18053926 | -0.640151247 | -0.720096882 | -0.58950698 | 0.32124617 | -0.203616257 | -1.59642767 | -1.81104503 |
| LINC01508 | -1.09494588 | 1.329534712 | 0.16672730 | 1.39623333 | 1.305084326 | 0.993871333 | 3.07217033 | 2.24015523 | 1.212160417 | -0.64337658 | -0.44217107 |
| LINC00707 | -3.69839512 | -2.046491446 | -0.82168162 | -1.44514119 | -0.401578019 | -0.026025737 | 0.41676862 | -1.02204864 | -0.926698272 | -2.20487395 | -1.74602530 |
| GATA6-AS1 | 1.08268031 | -0.542210994 | 0.35044578 | 1.30479888 | -0.470791911 | 0.346827586 | 0.73681061 | 0.40066674 | -0.787998596 | -0.04902550 | -1.08003039 |
| AC092580.4 | -0.22939662 | -1.556486407 | -0.60627454 | -0.77295105 | -1.162081897 | -0.283052985 | -0.06237084 | -0.45332312 | 0.086400051 | -0.91865938 | -0.18573830 |
| CTA-384D8.35 | -0.93425070 | -1.639934177 | -0.51103593 | -1.00215146 | -0.791475137 | -0.559713869 | -0.14214080 | 0.17295215 | 0.367293687 | -0.49696003 | 0.91986527 |
| CTA-384D8.34 | -1.46507390 | -1.359004146 | -0.38922048 | -0.52099132 | -0.824701081 | -0.308030944 | 0.05328385 | 0.11191103 | 0.762385618 | -0.74237222 | 0.58459098 |
| MIR4435-1HG | 0.47123279 | -0.073396124 | 0.34362462 | 0.26027335 | 0.633124344 | 0.338619021 | 0.94619696 | 0.30953098 | 1.048469607 | 0.54584958 | 1.84786324 |
| MLLT4-AS1 | 0.74030906 | 0.213397377 | 0.32062732 | 0.37662888 | 0.165130054 | 0.546309830 | 0.59234586 | 0.21880100 | -0.254408574 | 0.04952717 | 0.04937668 |
| EDNRB-AS1 | -1.83053471 | -1.664210708 | -1.71799941 | -1.66650668 | -2.263447706 | -1.093858050 | -2.22060498 | -0.98634056 | -0.646599224 | -2.86292265 | -2.98191350 |
| CTC-459F4.1 | -0.98923301 | -0.217021885 | 0.59863615 | -0.36002568 | -0.265334565 | 0.628944284 | 0.34759369 | -0.97915584 | -0.549142539 | -0.76730942 | -1.19646158 |
| MIAT | -0.29164217 | -2.185770423 | -0.07657509 | -0.45376073 | -0.894865185 | -0.192188816 | -0.59838353 | -0.75419280 | -0.621600490 | -0.60748981 | 0.39049826 |

In [159]:

```
options(repr.plot.width = 20, repr.plot.height = 4)

pheatmap::pheatmap(t(log2FcCalc), cluster_rows = T, cluster_cols = T, fontsize = 15, file = file.path(out.figs.dir, 'log2fc.high.vs.low.heatmap.pdf'), height = 5, width = 12)
```

In [160]:

```
pheatmap::pheatmap(t(log2FcCalc), cluster_rows = F, cluster_cols = F, fontsize = 15)
```

In [166]:

```
log2FcCalc <- lapply(sig.cancers, function(cancer) {
    obj <- readRDS(sprintf('../0.data/Pan.cancer/TCGA-%s-Obj.rds', cancer))
    obj.sub <- subset(obj, sample_type == 'Primary Tumor' | sample_type == 'Metastatic' | sample_type == 'Primary Blood Derived Cancer - Peripheral Blood')
    sub.clinical.tmp <- obj.sub@meta.data[, c('patient', 'vital_status', 'days_to_death', 'days_to_last_follow_up')]
    sub.clinical.tmp$Sur <- sub.clinical.tmp$vital_status == "Dead"
    sub.clinical.tmp$Time <- ifelse(sub.clinical.tmp$Sur, sub.clinical.tmp$days_to_death, sub.clinical.tmp$days_to_last_follow_up) / 365

    expr.tmp <- FetchData(obj.sub, vars = gsub('_', '-', hub.genes.sub))  %>% { log2(1 + .)}
    coefs <- mult.var.cox$coefficients[!is.na(mult.var.cox$coefficients)]
    select.sets <- names(coefs)
    risk.ss <- coefs %*% t(as.matrix(expr.tmp[, gsub('_', '-', select.sets)]))
    sub.clinical.tmp$Groups <- as.vector(risk.ss) 
    tmp.cox <- OS.analysis(sub.clinical.tmp, c('#8A0E1A', '#3574CE'), T, cutoff = T, prefix = sprintf('TCGA_%s_OS.pdf', cancer), risk.table = F, title = cancer, out.figs.dir = out.figs.dir)
    obj.sub@meta.data$Group <- tmp.cox$group
    obj.sub.high <- subset(obj.sub, Group == 'High risk')
    obj.sub.low <- subset(obj.sub, Group == 'Low risk')
        
    tumor.exprs <- colMeans(GetAssayData(obj.sub.high) %>% as.matrix %>% t)
    normal.exprs <- colMeans(GetAssayData(obj.sub.low) %>% as.matrix %>% t)
    log2(tumor.exprs / (0.01 + normal.exprs))   
}) %>% as.data.frame
```

In [170]:

```
names(log2FcCalc) <- sig.cancers
head(log2FcCalc)
```

A data.frame: 6 × 11

|  | ACC | BLCA | COAD | ESCA | HNSC | KIRP | LIHC | MESO | SARC | SKCM | UVM |
| --- | --- | --- | --- | --- | --- | --- | --- | --- | --- | --- | --- |
|  | <dbl> | <dbl> | <dbl> | <dbl> | <dbl> | <dbl> | <dbl> | <dbl> | <dbl> | <dbl> | <dbl> |
| DDX11L1 | -2.6708184 | -1.9176454 | -2.6765139 | -1.21112314 | -2.39678836 | -3.2088295 | -3.91350197 | -2.99619023 | -0.9412216 | -2.7810242 | -2.5395898 |
| RP11-34P13.7 | -1.8228427 | -1.7320678 | -0.9854273 | -1.30039179 | -2.21417603 | -1.0598186 | -0.22805256 | -2.12210401 | -3.2813101 | -0.3249161 | -2.2781604 |
| RP5-902P8.10 | -0.2332325 | 0.2379689 | 0.2842175 | -0.10200508 | -0.24962361 | 0.3335991 | 0.07828077 | -0.65806357 | -0.1705939 | -0.2802490 | -1.0331416 |
| RP4-591L5.1 | -1.2325330 | -3.1173976 | -2.4348101 | -3.32527672 | -3.86517966 | -2.4133059 | -Inf | -Inf | -0.7825438 | -3.4835092 | -3.7243534 |
| SUGT1P2 | -0.1208721 | 0.6204161 | 1.0712642 | 0.20530858 | -0.29790337 | 0.1022152 | -0.08222804 | -0.38933395 | 0.1395837 | -0.3497752 | -0.6272682 |
| AC139452.2 | 0.4003081 | 0.1403697 | -0.1169840 | 0.06853676 | -0.08240941 | 0.2578758 | 0.31908922 | -0.05663115 | 0.2304006 | -0.4348239 | 0.3468955 |

In [165]:

```
ctl.score <- list(CTL = c('CD8A', 'CD8B', 'GZMA', 'GZMB', 'PRF1'))
```

In [171]:

```
anno <- read.table('../0.data/gencode.gene.info.v22.tsv', sep = '\t', header = T) %>% subset(., gene_type == 'protein_coding')
head(anno)
com.genes <- intersect(rownames(tcga.obj.sub),anno$gene_name)
length(com.genes)
```

A data.frame: 6 × 12

|  | gene\_id | gene\_name | seqname | start | end | strand | gene\_type | gene\_status | havana\_gene | full\_length | exon\_length | exon\_num |
| --- | --- | --- | --- | --- | --- | --- | --- | --- | --- | --- | --- | --- |
|  | <chr> | <chr> | <chr> | <int> | <int> | <chr> | <chr> | <chr> | <chr> | <int> | <int> | <int> |
| 7 | ENSG00000206557.5 | TRIM71 | chr3 | 32818018 | 32897826 | + | protein\_coding | KNOWN | OTTHUMG00000155778.3 | 79809 | 8685 | 4 |
| 8 | ENSG00000183813.6 | CCR4 | chr3 | 32951574 | 32956349 | + | protein\_coding | KNOWN | OTTHUMG00000130752.2 | 4776 | 3095 | 2 |
| 9 | ENSG00000170266.14 | GLB1 | chr3 | 32996608 | 33097230 | - | protein\_coding | KNOWN | OTTHUMG00000155781.6 | 100623 | 4364 | 123 |
| 12 | ENSG00000188167.7 | TMPPE | chr3 | 33090421 | 33096801 | - | protein\_coding | KNOWN | OTTHUMG00000155779.1 | 6381 | 3966 | 4 |
| 14 | ENSG00000170275.13 | CRTAP | chr3 | 33113979 | 33147773 | + | protein\_coding | KNOWN | OTTHUMG00000130746.3 | 33795 | 6695 | 18 |
| 17 | ENSG00000173705.7 | SUSD5 | chr3 | 33150042 | 33219215 | - | protein\_coding | KNOWN | OTTHUMG00000155829.2 | 69174 | 5008 | 9 |

19459

In [172]:

```
log2FcCalc <- log2FcCalc[com.genes, ]
```

In [173]:

```
dim(log2FcCalc)
```

1. 19459
2. 11

In [272]:

```
order.tar <- log2FcCalc[, 'BLCA']
names(order.tar) <- rownames(log2FcCalc)
order.tar <- order.tar[!is.na(order.tar)]
order.tar <- order.tar[order.tar != -Inf]
order.tar <- sort(order.tar, decreasing = T)
```

In [273]:

```
markers.sub <- subset(markers, Immunity == 'Adaptive')$Metagene
length(markers.sub)
```

431

In [274]:

```
options(repr.plot.width = 10, repr.plot.height = 4)
p1 <- plotEnrichment(markers.sub, order.tar, ticksSize  = 1) + theme_bw(base_size = 20) + ylab('Enrichment score') + xlab('Rank') + ggtitle('BLCA') + theme(plot.title = element_text(hjust = 0.5))
p1
```

In [275]:

```
order.tar <- log2FcCalc[, 'UVM']
names(order.tar) <- rownames(log2FcCalc)
order.tar <- order.tar[!is.na(order.tar)]
order.tar <- order.tar[order.tar != -Inf]
order.tar <- sort(order.tar, decreasing = T)
```

In [276]:

```
options(repr.plot.width = 10, repr.plot.height = 4)
p2 <- plotEnrichment(markers.sub, order.tar, ticksSize  = 1) + theme_bw(base_size = 20) + ylab('Enrichment score') + xlab('Rank') + ggtitle('UVM') + theme(plot.title = element_text(hjust = 0.5))
p2
```

In [279]:

```
order.tar <- log2FcCalc[, 'ACC']
names(order.tar) <- rownames(log2FcCalc)
order.tar <- order.tar[!is.na(order.tar)]
order.tar <- order.tar[order.tar != -Inf]
order.tar <- sort(order.tar, decreasing = T)
p3 <- plotEnrichment(markers.sub, order.tar, ticksSize  = 1) + theme_bw(base_size = 20) + ylab('Enrichment score') + xlab('Rank') + ggtitle('ACC') + theme(plot.title = element_text(hjust = 0.5))
p3
```

In [282]:

```
options(repr.plot.width = 10, repr.plot.height = 8)
ggarrange(p3, p1, p2, nrow = 3)
ggsave(file.path(out.figs.dir, 'gsea.immune.pdf'), width = 8, height = 10)
```

In [257]:

```
plotEnrichment <- function (pathway, stats, gseaParam = 1, ticksSize = 0.2) 
{
    rnk <- rank(-stats)
    ord <- order(rnk)
    statsAdj <- stats[ord]
    statsAdj <- sign(statsAdj) * (abs(statsAdj)^gseaParam)
    statsAdj <- statsAdj/max(abs(statsAdj))
    pathway <- unname(as.vector(na.omit(match(pathway, names(statsAdj)))))
    pathway <- sort(pathway)
    gseaRes <- calcGseaStat(statsAdj, selectedStats = pathway, 
        returnAllExtremes = TRUE)
    bottoms <- gseaRes$bottoms
    tops <- gseaRes$tops
    n <- length(statsAdj)
    xs <- as.vector(rbind(pathway - 1, pathway))
    ys <- as.vector(rbind(bottoms, tops))
    toPlot <- data.frame(x = c(0, xs, n + 1), y = c(0, ys, 0))
    diff <- (max(tops) - min(bottoms))/8
    x = y = NULL
    g <- ggplot(toPlot, aes(x = x, y = y)) + geom_point(color = "green", 
        size = 0.5) + geom_hline(yintercept = max(tops), colour = "red", 
        linetype = "dashed", size = 1) + geom_hline(yintercept = min(bottoms), 
        colour = "red", linetype = "dashed", size = 1.0) + geom_hline(yintercept = 0, 
        colour = "black", size = 1.0) + geom_line(color = "green", size = 1.5) + theme_bw() + 
        geom_segment(data = data.frame(x = pathway), mapping = aes(x = x, 
            y = -diff/2, xend = x, yend = diff/2), size = ticksSize) + 
        theme(panel.border = element_blank(), panel.grid.minor = element_blank()) + 
        labs(x = "rank", y = "enrichment score")
    g
}
```

In [1370]:

```
sessionInfo()
```

```
R version 4.1.0 (2021-05-18)
Platform: x86_64-w64-mingw32/x64 (64-bit)
Running under: Windows 10 x64 (build 22000)

Matrix products: default

locale:
[1] LC_COLLATE=Chinese (Simplified)_China.936 
[2] LC_CTYPE=Chinese (Simplified)_China.936   
[3] LC_MONETARY=Chinese (Simplified)_China.936
[4] LC_NUMERIC=C                              
[5] LC_TIME=Chinese (Simplified)_China.936    

attached base packages:
[1] grid      stats     graphics  grDevices utils     datasets  methods  
[8] base     

other attached packages:
 [1] preprocessCore_1.54.0       survival_3.2-11            
 [3] survminer_0.4.9             ggpubr_0.4.0               
 [5] forestplot_1.10.1           checkmate_2.0.0            
 [7] magrittr_2.0.1              survivalROC_1.0.3          
 [9] limma_3.48.1                glmnet_4.1-2               
[11] ConsensusClusterPlus_1.56.0 readxl_1.3.1               
[13] metafor_3.0-2               Matrix_1.3-3               
[15] ggplot2_3.3.5               GSVA_1.40.1                
[17] SeuratObject_4.0.2          Seurat_4.0.3               
[19] dplyr_1.0.7                

loaded via a namespace (and not attached):
  [1] pbdZMQ_0.3-5                scattermore_0.7            
  [3] exactRankTests_0.8-32       lavaan_0.6-9               
  [5] GSEA_1.2                    tidyr_1.1.3                
  [7] bit64_4.0.5                 knitr_1.33                 
  [9] irlba_2.3.3                 DelayedArray_0.18.0        
 [11] data.table_1.14.0           rpart_4.1-15               
 [13] doParallel_1.0.16           KEGGREST_1.32.0            
 [15] RCurl_1.98-1.3              generics_0.1.0             
 [17] BiocGenerics_0.38.0         ScaledMatrix_1.0.0         
 [19] callr_3.7.0                 cowplot_1.1.1              
 [21] usethis_2.0.1               RSQLite_2.2.7              
 [23] RANN_2.6.1                  future_1.21.0              
 [25] bit_4.0.4                   xml2_1.3.2                 
 [27] spatstat.data_2.1-0         httpuv_1.6.1               
 [29] SummarizedExperiment_1.22.0 amap_0.8-18                
 [31] xfun_0.24                   hms_1.1.0                  
 [33] evaluate_0.14               promises_1.2.0.1           
 [35] DEoptimR_1.0-9              fansi_0.5.0                
 [37] caTools_1.18.2              km.ci_0.5-2                
 [39] igraph_1.2.6                DBI_1.1.1                  
 [41] tmvnsim_1.0-2               htmlwidgets_1.5.3          
 [43] apcluster_1.4.8             spatstat.geom_2.2-2        
 [45] stats4_4.1.0                purrr_0.3.4                
 [47] ellipsis_0.3.2              corrplot_0.90              
 [49] backports_1.2.1             markdown_1.1               
 [51] pbivnorm_0.6.0              annotate_1.70.0            
 [53] deldir_0.2-10               sparseMatrixStats_1.4.0    
 [55] MatrixGenerics_1.4.0        vctrs_0.3.8                
 [57] SingleCellExperiment_1.14.1 Biobase_2.52.0             
 [59] remotes_2.4.0               ROCR_1.0-11                
 [61] abind_1.4-5                 cachem_1.0.5               
 [63] withr_2.4.2                 robustbase_0.93-8          
 [65] sctransform_0.3.2           prettyunits_1.1.1          
 [67] fdrtool_1.2.16              mclust_5.4.7               
 [69] goftest_1.2-2               mnormt_2.0.2               
 [71] cluster_2.1.2               IRdisplay_1.0              
 [73] lazyeval_0.2.2              crayon_1.4.1               
 [75] pkgconfig_2.0.3             labeling_0.4.2             
 [77] GenomeInfoDb_1.28.1         pkgload_1.2.1              
 [79] nlme_3.1-152                devtools_2.4.2             
 [81] nnet_7.3-16                 rlang_0.4.11               
 [83] globals_0.14.0              lifecycle_1.0.0            
 [85] miniUI_0.1.1.1              biwt_1.0                   
 [87] mathjaxr_1.4-0              rsvd_1.0.5                 
 [89] rprojroot_2.0.2             cellranger_1.1.0           
 [91] polyclip_1.10-0             matrixStats_0.59.0         
 [93] lmtest_0.9-38               graph_1.70.0               
 [95] IRkernel_1.2.0.9000         KMsurv_0.1-5               
 [97] carData_3.0-4               Rhdf5lib_1.14.2            
 [99] zoo_1.8-9                   base64enc_0.1-3            
[101] processx_3.5.2              pheatmap_1.0.12            
[103] ggridges_0.5.3              png_0.1-7                  
[105] viridisLite_0.4.0           bitops_1.0-7               
[107] KernSmooth_2.23-20          rhdf5filters_1.4.0         
[109] Biostrings_2.60.1           blob_1.2.2                 
[111] DelayedMatrixStats_1.14.0   shape_1.4.6                
[113] maxstat_0.7-25              stringr_1.4.0              
[115] parallelly_1.27.0           jpeg_0.1-8.1               
[117] rstatix_0.7.0               S4Vectors_0.30.0           
[119] ggsignif_0.6.2              beachmat_2.8.0             
[121] scales_1.1.1                memoise_2.0.0              
[123] GSEABase_1.54.0             plyr_1.8.6                 
[125] ica_1.0-2                   gplots_3.1.1               
[127] zlibbioc_1.38.0             compiler_4.1.0             
[129] RColorBrewer_1.1-2          rrcov_1.5-5                
[131] fitdistrplus_1.1-5          cli_3.0.1                  
[133] XVector_0.32.0              listenv_0.8.0              
[135] ps_1.6.0                    patchwork_1.1.1            
[137] pbapply_1.4-3               htmlTable_2.2.1            
[139] Formula_1.2-4               MASS_7.3-54                
[141] mgcv_1.8-35                 tidyselect_1.1.1           
[143] stringi_1.6.2               forcats_0.5.1              
[145] BiocSingular_1.8.1          latticeExtra_0.6-29        
[147] ggrepel_0.9.1               survMisc_0.5.5             
[149] tools_4.1.0                 future.apply_1.7.0         
[151] parallel_4.1.0              rio_0.5.27                 
[153] rstudioapi_0.13             uuid_0.1-4                 
[155] foreach_1.5.1               foreign_0.8-81             
[157] gridExtra_2.3               farver_2.1.0               
[159] Rtsne_0.15                  digest_0.6.27              
[161] ggtext_0.1.1                shiny_1.6.0                
[163] gridtext_0.1.4              Rcpp_1.0.7                 
[165] GenomicRanges_1.44.0        car_3.0-11                 
[167] broom_0.7.8                 later_1.2.0                
[169] RcppAnnoy_0.0.18            httr_1.4.2                 
[171] AnnotationDbi_1.54.1        psych_2.1.6                
[173] colorspace_2.0-2            fs_1.5.0                   
[175] XML_3.99-0.6                tensor_1.5                 
[177] reticulate_1.20             IRanges_2.26.0             
[179] splines_4.1.0               uwot_0.1.10                
[181] spatstat.utils_2.2-0        sessioninfo_1.1.1          
[183] plotly_4.9.4.1              xtable_1.8-4               
[185] jsonlite_1.7.2              corpcor_1.6.9              
[187] glasso_1.11                 testthat_3.0.4             
[189] R6_2.5.0                    Hmisc_4.5-0                
[191] pillar_1.6.1                htmltools_0.5.1.1          
[193] mime_0.11                   glue_1.4.2                 
[195] fastmap_1.1.0               BiocParallel_1.26.1        
[197] class_7.3-19                codetools_0.2-18           
[199] pkgbuild_1.2.0              pcaPP_1.9-74               
[201] mvtnorm_1.1-2               utf8_1.2.1                 
[203] lattice_0.20-44             spatstat.sparse_2.0-0      
[205] tibble_3.1.2                curl_4.3.2                 
[207] leiden_0.3.8                gtools_3.9.2               
[209] kohonen_3.0.10              cogena_1.26.0              
[211] zip_2.2.0                   openxlsx_4.2.4             
[213] qgraph_1.6.9                repr_1.1.3                 
[215] desc_1.3.0                  munsell_0.5.0              
[217] fastcluster_1.2.3           rhdf5_2.36.0               
[219] GenomeInfoDbData_1.2.6      iterators_1.0.13           
[221] HDF5Array_1.20.0            haven_2.4.1                
[223] reshape2_1.4.4              gtable_0.3.0               
[225] spatstat.core_2.3-0
```

In [ ]:

```

```

In [ ]:

```

```
